# Alkene Isomerization Catalyzed by a Mn(I) Bisphosphine Borohydride Complex

**DOI:** 10.1021/acscatal.4c03364

**Published:** 2024-08-17

**Authors:** Ines Blaha, Stefan Weber, Robin Dülger, Luis F. Veiros, Karl Kirchner

**Affiliations:** † Institute of Applied Synthetic Chemistry, TU Wien, Getreidemarkt 9/163-AC, A-1060 Wien, Austria; ‡ Centro de Química Estrutural, Institute of Molecular Sciences, Departamento de Engenharia Química, Instituto Superior Técnico, Universidade de Lisboa, Av. Rovisco Pais, 1049 001 Lisboa, Portugal

**Keywords:** isomerization, manganese, borohydride, alkenes, DFT calculations

## Abstract

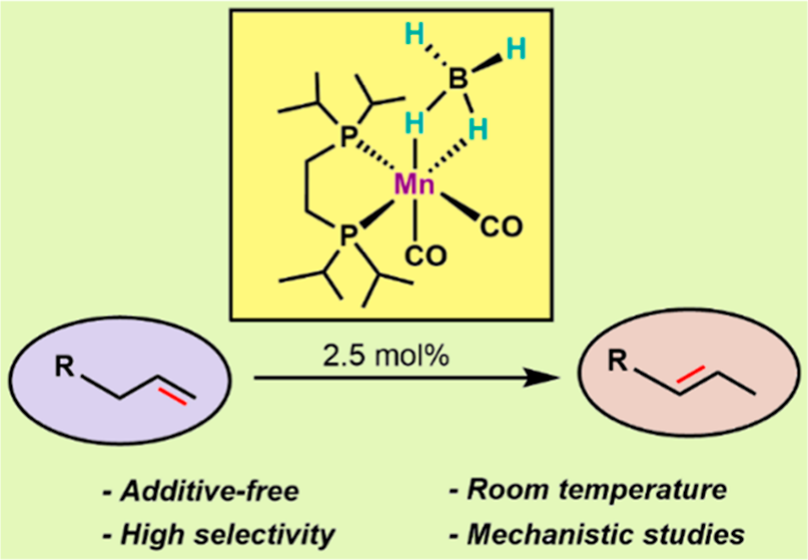

An additive-free manganese-catalyzed isomerization of terminal alkenes to internal alkenes is described. This reaction is implementing an inexpensive nonprecious metal catalyst. The most efficient catalyst is the borohydride complex *cis*-[Mn(dippe)(CO)_2_(κ^2^-BH_4_)]. This catalyst operates at room temperature, with a catalyst loading of 2.5 mol %. A variety of terminal alkenes is effectively and selectively transformed into the respective internal *E*-alkenes. Preliminary results show chain-walking isomerization at an elevated temperature. Mechanistic studies were carried out, including stoichiometric reactions and in situ NMR analysis. These experiments are flanked by computational studies. Based on these, the catalytic process is initiated by the liberation of “BH_3_” as a THF adduct. The catalytic process is initiated by double bond insertion into an M-H species, leading to an alkyl metal intermediate, followed by β-hydride elimination at the opposite position to afford the isomerization product.

## Introduction

**Figure 1:**
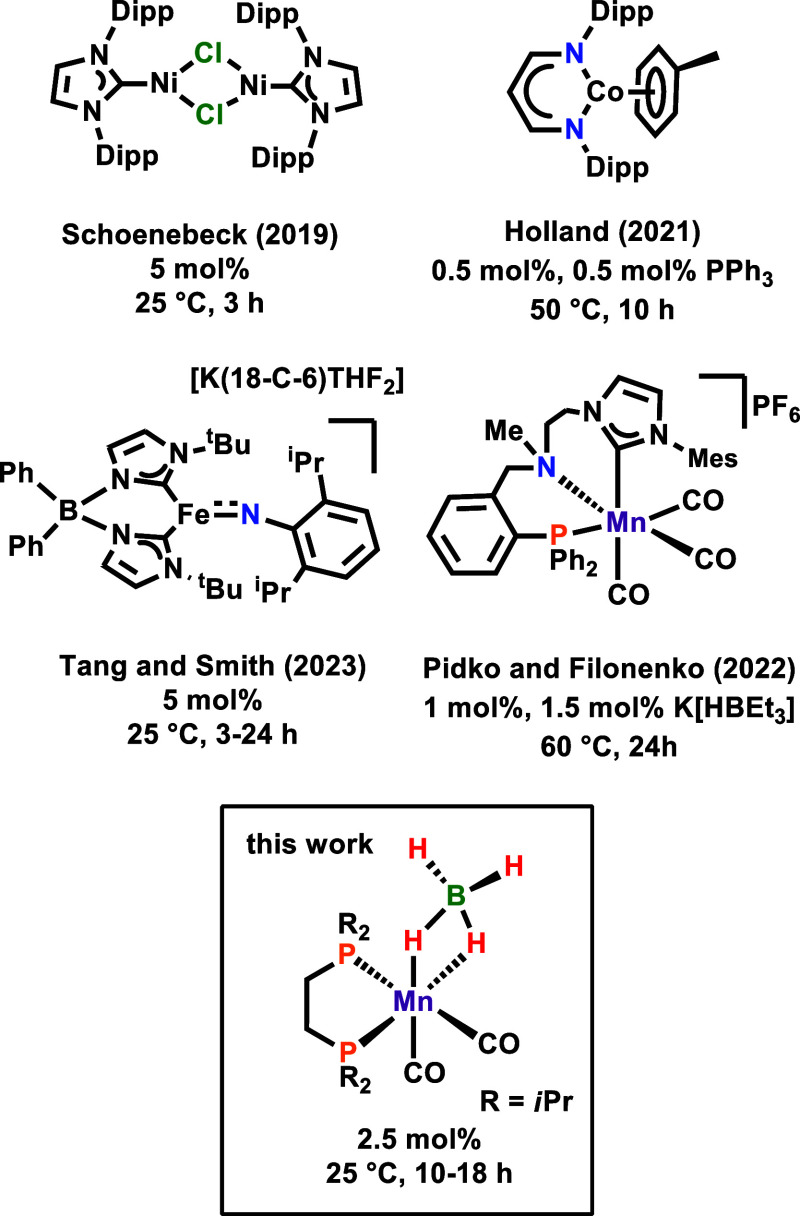
Selected examples of base metal olefin isomerization precatalysts.

Isomerization and translocation of double bonds inside a molecule are essential steps in a variety of catalytic processes. Monoisomerization and especially chain-walking reactions enable the functionalization of a great variety of organic frameworks.^1^ The compounds thus obtained are widely used as fragrances, agrochemicals, and intermediates in the pharmaceutical industry.^2^ The field of transpositional isomerization catalysis has long been dominated by complexes based on precious metals, such as rhodium,^3^ iridium,^4^ ruthenium,^5^ and palladium.^6^ However, representatives of base metal catalysts in this field have been fathomed.^7^ The early development of nickel-^8^ and cobalt-based^9^ isomerization catalysts as well as industrial application of the latter^10^ represents some of the most established examples. Over the last years, base metal catalysts based on nickel,^11^ cobalt,^12^ and iron^13^ have emerged in the field of alkene isomerization.^7,14^ Selected base metal catalysts for the isomerization of alkenes are depicted in Figure 1.

As manganese is concerned, to the best of our knowledge, there is only one literature example described by Pidko and Filonenko.^15^ The authors utilized a cationic Mn(I) CNP pincer carbonyl precatalyst which, in the presence of K[HBEt_3_], was able to isomerize terminal carbon double bonds at 60 °C.

We have recently demonstrated the broad application of manganese(I) complexes based on *fac*-[Mn(dippe)(CO)_3_(CH_2_CH_2_CH_3_)] (dippe = 1,2-bis(di-*iso*-propylphosphino)ethane) for hydrogenation and hydrofunctionalization reactions.^16,17^ Based on our recent findings, we describe here the activity of *fac*-[Mn(dippe)(CO)_3_(CH_2_CH_2_CH_3_)] (**Mn1**),^17a^*fac*-[Mn(dippe)(CO)_3_(H)] (**Mn2**),^18^*cis*-[Mn(dippe)(CO)_2_(κ^2^-HBpin)] (**Mn3**),^17c^ and *cis*-[Mn(dippe)(CO)_2_(κ^2^-BH_4_)] (**Mn4**) as precatalysts for the selective isomerization of terminal alkenes to internal alkenes. A plausible reaction mechanism based on detailed experimental and theoretical studies is presented.

## Results and Discussion

The new Mn(I) borohydride complex **Mn4** was prepared in 79% yield by the treatment of **Mn1** with NH_3_BH_3_ at 50 °C for 3.5 h. It was fully characterized by ^1^H, ^13^C{^1^H}, and ^31^P{^1^H} NMR and IR spectroscopy and high-resolution mass spectrometry (HR-MS). The catalytic performance of **Mn4** and the known Mn(I) complexes **Mn1–Mn3** were then investigated for the isomerization of 1-allyl-4-fluorobenzene as a model substrate in THF as a solvent. Selected optimization experiments are depicted in Table 1. Although **Mn1** effectively catalyzes a variety of hydrofunctionalizations,^16^ it turned out to be catalytically inactive for isomerization purposes. Likewise, hydride complex **Mn2** was found to be inactive for the desired transformation. With **Mn3** bearing the σ–B–H-bound HBpin ligand, full conversion to the internal alkene was achieved at 60 °C. However, lowering the temperature to 25 °C resulted in no catalytic activity. **Mn4** afforded high conversion and yield with very good selectivity toward the *E*-isomer even at room temperature within 10 h. Notably, no additives were required to activate either **Mn3** or **Mn4**. In other solvents such as CH_2_Cl_2_ and toluene, **Mn4** was considerably less active and lower yields were achieved, whereas in DMSO, acetone, and MeOH, no reaction took place at all (Table 1, entries 8–12). Finally, in the absence of a catalyst, no reaction took place.

**Table 1:**
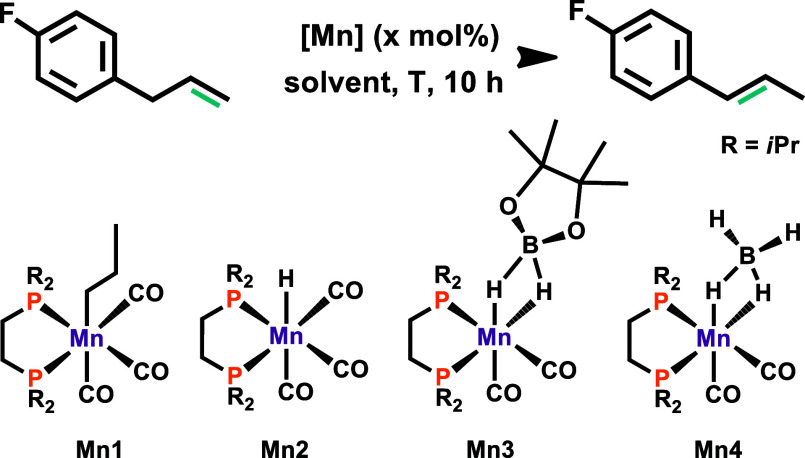
Optimization Reactions for the Manganese-Catalyzed Transposition of 1-Allyl-4-fluorobenzene^a^

entry	**[Mn]** (mol %)	solvent	temp (°C)	conversion (%)	*E*/*Z* ratio
1	**Mn1** (3)	THF	60		
2	**Mn2** (3)	THF	60		
3	**Mn3** (3)	THF	60	>99	94:6
4	**Mn4** (3)	THF	60	>99	99:1
5	**Mn3** (3)	THF	25		
6	**Mn4** (2.5)	THF	25	>99	99:1
7	**Mn4** (1)	THF	25	8	91:9
8	**Mn4** (2.5)	CH_2_Cl_2_	25	60	91:9
9	**Mn4** (2.5)	toluene	25	42	95:5
10	**Mn4** (2.5)	DMSO	25		
11	**Mn4** (2.5)	acetone	25		
12	**Mn4** (2.5)	MeOH	25		

a Reaction conditions: 1-allyl-4-fluorobenzene (0.5 mmol), **[Mn]**, and 1,4-dioxane as an internal standard (0.13 mmol) in the respective solvent (0.5 mL) for 10 h. Conversion and the *E*/*Z* ratio were determined by ^1^H NMR spectroscopy.

Based on the optimized reaction conditions, the substrate scope and limitations were investigated. In order to achieve sufficient conversion for a broad variety of substrates, the reaction time was extended to 18 h. Starting with aromatic substrates, the desired 1,2-isomerized compound was obtained in high yields. Allylbenzene derivatives featuring strong electron-donating groups (e.g., −OTMS) to weak electron-donating groups (e.g., alkyl moieties) or electron-withdrawing groups (e.g., single or multiple fluorine atoms) were all successfully converted to the desired alkenes with excellent *E*-selectivity. Halides, ethers, and trimethylsilylethers (Table 2, **1a**, **3a**, and **4a**) were well tolerated. A good performance on annulated systems is demonstrated by the isomerization of 1-allylnaphthalene (**6a**). The introduction of an *ortho*-methyl group led to a minor drop in selectivity (**8a**). A drastic decrease in selectivity was noted for mesityl substrate **11a** (Table 2). Although an excellent yield was upheld, a strong interference of the methyl groups can be presumed from the *E*/*Z* ratio being decreased to about 3:2. Heterocycles such as thiophene and furan moieties were well tolerated (Table 2, **14a** and **15a**). However, it should be noted that 40 °C was required for these heteroaromatic systems.

**Table 2:**
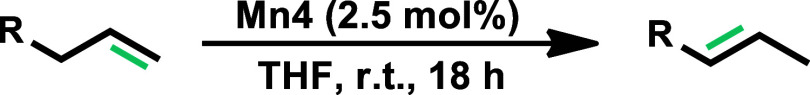

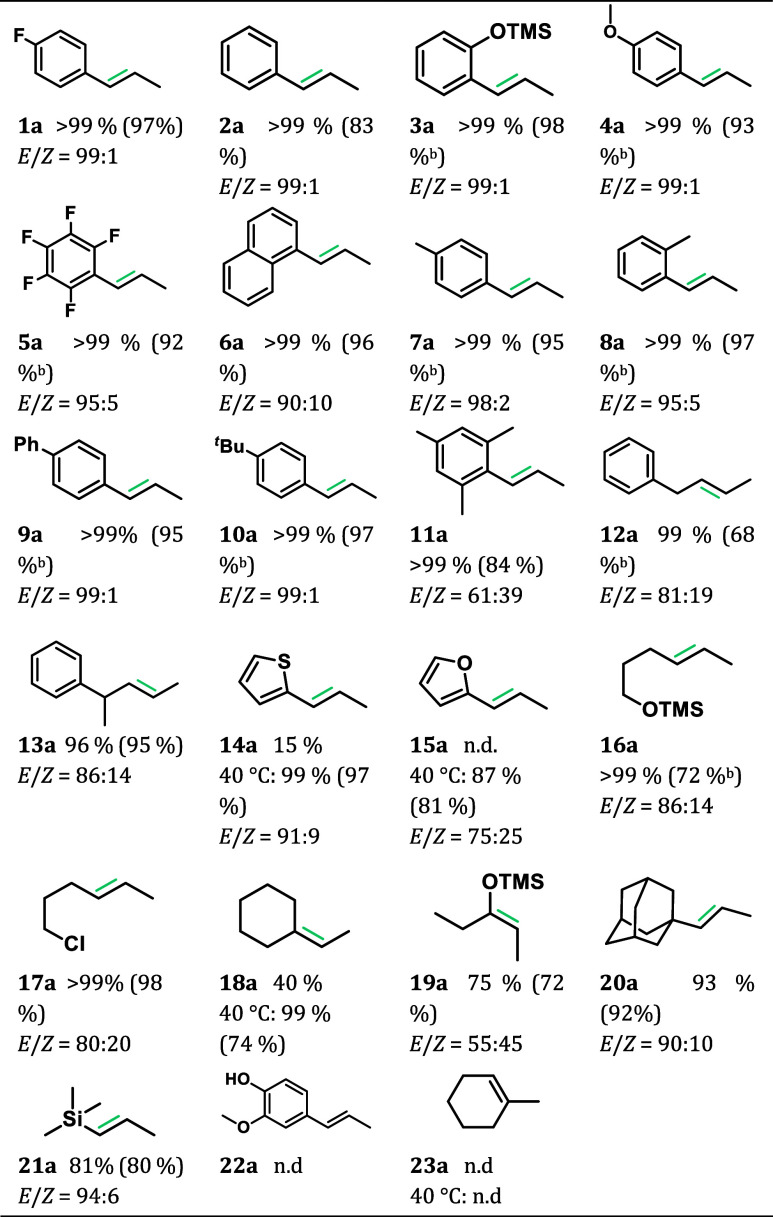
Catalytic 1,2-Transposition Reactions of Alkenes by **Mn4**^a^

a Reaction conditions: alkene substrate (0.5 mmol), **Mn4** (2.5 mol %), and 1,4-dioxane as an internal standard (0.13 mmol) in THF-*d*_8_ (0.5 mL) at 25 °C for 18 h. Conversion, yield, and the *E*/*Z* ratio were determined by gas chromatography–MS (GC–MS) and ^1^H NMR spectroscopy. Results represent an average of two runs.

b Isolated yield. n.d. = not detected.

While high reactivity was achieved for aliphatic systems, a drop in selectivity was observed (Table 2, **16a** and **17a**). This effect increased to a mere 55:45 *E*/*Z* ratio for trimethyl(pent-1-en-3-yloxy)silane (Table 2, **19a**). Gratifyingly, more bulky olefins such as allyladamantane and allyltrimethylsilane underwent facile isomerization to afford **20a** and **21a** in high yields (Table 2). In comparison to the model substrate 1-allyl-4-fluorobenzene, a slight decrease in catalytic performance was noted, but high *E/Z* ratios were achieved. The conversion of vinylcyclohexane to the trisubstituted olefin ethylidenecyclohexane (Table 2, **18a**) was explored. At room temperature, a conversion of 40% was not exceeded even with an extended reaction time of 36 h. However, heating to 40 °C for 18 h afforded **18a** in 99% yield. No conversion could be observed for unprotected phenols, such as eugenol (Table 2, **22a**).

Encouraged by these findings, we envisioned the application of **Mn4** for the gram-scale synthesis of *E*-4-(prop-1-en-1-yl)-1,1′-biphenyl from 4-allyl-1,1′-biphenyl (Table 1, **9a**). Satisfyingly, an isolated yield of 86% could be achieved, while a high product selectivity of 99:1 in favor of the *E*-isomer was upheld.

**Scheme 1:**
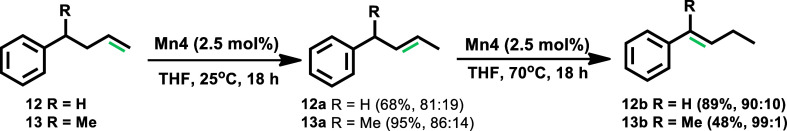
Preliminary Studies on Chain-Walking Isomerization Catalyzed by **Mn4**

Preliminary studies of long-distance isomerization (chain walking) were conducted by increasing the reaction temperature. At room temperature, olefins **12** and **13** were converted into **12a** and **13a**, respectively, in 68 and 95% yields with *E*/*Z* ratios of 81:19 and 86:14 (Scheme 1). Upon addition of a new batch of catalyst at 70 °C, these olefins were further converted into the conjugated analogues **12b** and **13b** in 89 and 48% yields, respectively, with *E/Z* ratios of 90:10 and 99:1. It should be noted that in the case of the nonbranched terminal alkene **12**, partial chain walking occurred already at room temperature, accounting for the lower yield of **12a**. For the sterically more demanding **13a**, the second transposition step is significantly more difficult even at a reaction temperature of 70 °C, leading to a lower yield of **13b**. The formation of **12b** and **13b** from **12** and **13**, respectively, was also achieved in one step with similar yields and *E/Z* ratios upon performing the reaction at 70 °C for 18 h. It has to be mentioned that in the case of the aliphatic substrates **16** and **17**, no chain walking was observed.

Having established the scope of the isomerization procedure, we turned our attention toward the investigation of the reaction mechanism. Over the last decades, different mechanisms for olefin isomerization by transition metals have been proposed.^7,13b,19^ Among those, the alkyl and radical mechanisms are the most prevalent. The alkyl mechanism involves the insertion of an olefin into a metal hydride, followed by β-hydride elimination to furnish the product. Following a similar sequence, the radical mechanism is based on the transfer of hydrogen radicals. In contrast to the previously mentioned pathways, the π-allyl mechanism proceeds exclusively in an intramolecular fashion through a 1,3-hydrogen shift.

**Scheme 2:**
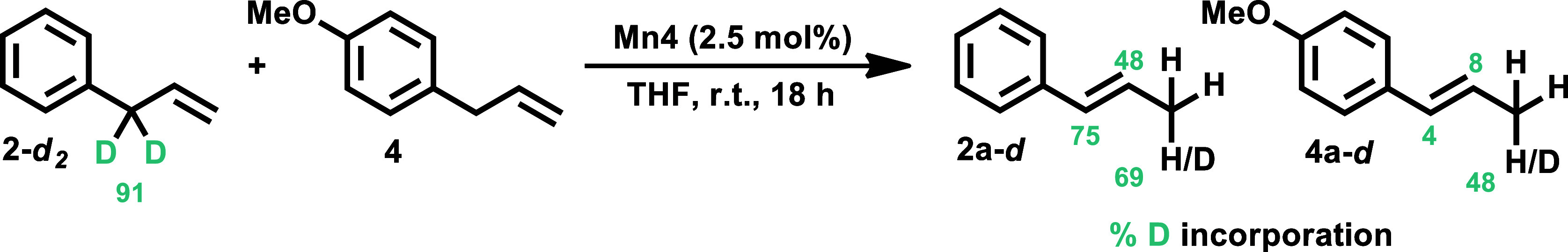
Deuterium Crossover Labeling Experiment with **Mn4**

A well-established method to distinguish the possible pathways described above is the crossover labeling experiment.^15^ We performed a crossover isomerization of allylbenzene-*d*_2_ (**2**-*d*_2_) and nondeuterated 4-allylanisole (**4**) (Scheme 2). An intramolecular hydrogen shift involved in the allyl mechanism would confine deuterium to the previously labeled substrate **2**-*d*_2_. However, deuterium scrambling was found to extend to 4-allylanisole, yielding partially deuterated (*E*)-anethole-*d* (**4a**-*d*). Furthermore, a distribution of deuterium throughout the product propene chains was observed. Due to these findings, the involvement of an π-allyl mechanism in the isomerization catalyzed by **Mn4** was excluded.

To investigate the potential involvement of a radical mechanism, we performed an experiment with TEMPO as a radical trap. The presence of TEMPO is presumed to inhibit isomerization via a radical pathway. However, the presence of the radical trap did not affect the isomerization of compound **1** (Table 3, entry 1). No products of a reaction between TEMPO and a substrate radical could be detected. Therefore, we deem the involvement of a radical mechanism unlikely. Further mechanistic investigations focused on the hydride functionality, accompanied by an easily accessible coordination site. In order to test this assumption, pyridine and PMe_3_ were used as the additives.

**Table 3:**
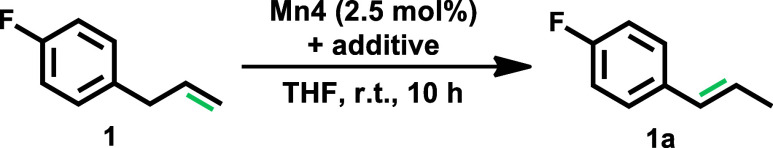
Isomerization Experiments with Additives^a^

entry	additive (mol %)	solvent	yield (%)	*E*/*Z* ratio
1	TEMPO (12.5)	THF	97	99:1
2	pyridine (12.5)	THF	77	96:4
3	PMe_3_ (12.5)	THF		

a Reaction conditions: 1-allyl-4-fluorobenzene (0.5 mmol), **Mn4** (2.5 mol %), additive (12.5 mol %), and 1,4-dioxane as an internal standard (0.13 mmol) in THF-*d*_8_ (0.5 mL) at 25 °C for 10 h. Yield and the *E*/*Z* ratio were determined by ^19^F and ^1^H NMR spectroscopy.

**Scheme 3:**
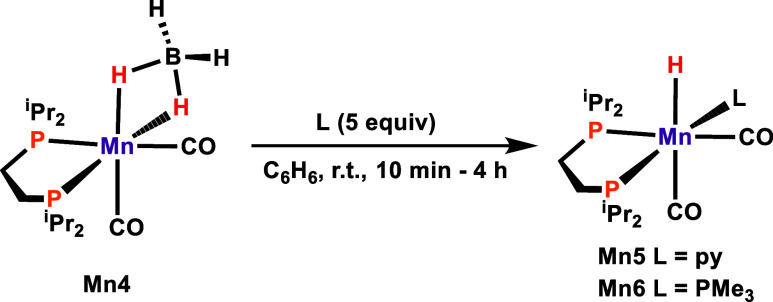
Syntheses of Hydride Complexes **Mn5** and **Mn6**

The addition of pyridine (Table 3, entry 2) resulted in a significant decrease in the reactivity of **Mn4**. The isomerization of allylbenzene resulted in a yield of 77%, while in the presence of PMe_3_, no catalytic activity was observed (Table 3, entry 3). A deactivation by the strongly coordinating pyridine and PMe_3_ ligands is in line with the presence of a vacant coordination site during catalysis. Monitoring of the experiments involving pyridine and PMe_3_ by ^31^P{^1^H} NMR spectroscopy revealed the formation of new hydride species giving rise to signals at −4.52 (t, *J*_HP_ = 54.8 Hz) and −9.00 (q, *J*_HP_ = 49.3 Hz) ppm, respectively. These species are assigned to complexes **Mn5** and **Mn6**, as shown in Scheme 3. While **Mn5** could not be isolated in pure form, **Mn6** could be isolated in 87% yield. Both compounds were fully characterized by NMR spectroscopy and HR-MS (Supporting Information).

Attempts to in situ generate a coordinatively unsaturated hydride complex from **Mn1** and H_2_ or NaBH_4_/MeOH did not result in productive catalysis.

The above-mentioned findings strongly suggest an inner-sphere mechanism with a catalyst activation by loss of “BH_3_” as the rate-determining step. Deuterium labeling and radical trap experiments are consistent with a hydride mechanism operating via olefin insertion and β-hydride elimination, which is indeed supported by density functional theory (DFT) calculations (vide infra).

**Figure 2:**
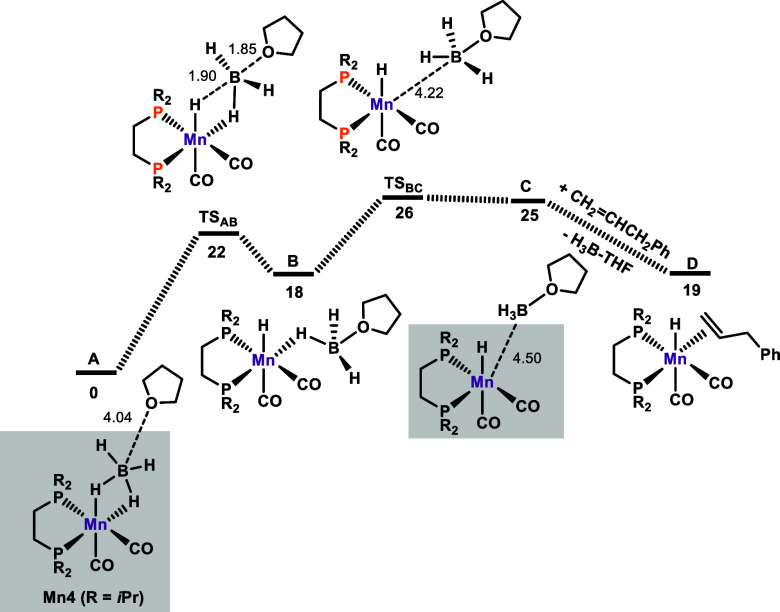
Free energy profile calculated for the activation of the precatalyst by the formation of a BH_3_·THF adduct. Free energies (kcal/mol) are referred to *fac*-[Mn(dippe)(CO)_2_(κ^2^-BH_4_)] (**Mn4**) (**A** in the calculation in the form of **Mn4**·THF).

**Figure 3:**
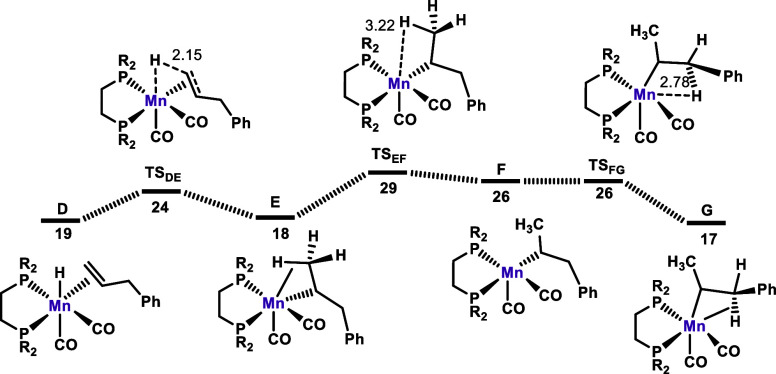
Free energy profile calculated for the isomerization of allylbenzene. Free energies (kcal/mol) are referred to *fac*-[Mn(dippe)(CO)_2_(κ^2^-BH_4_)] (**Mn4**) (**A** in the calculation in the form of **Mn4**·THF).

**Figure 4:**
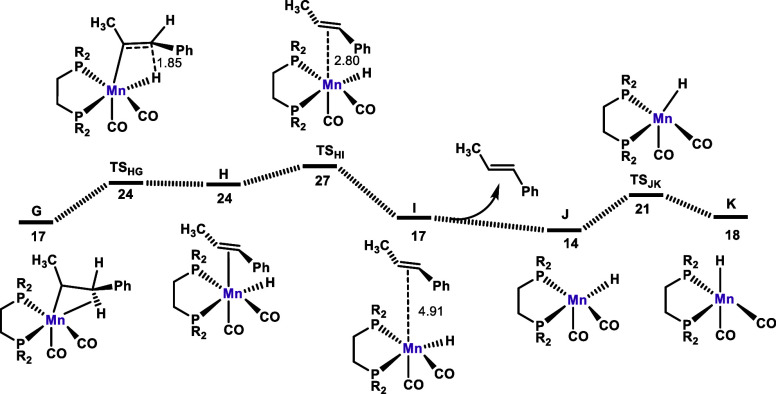
Free energy profile calculated for the isomerization of allylbenzene to form *E*-prop-1-en-1-ylbenzene. Free energies (kcal/mol) are referred to *fac*-[Mn(dippe)(CO)_2_(κ^2^-BH_4_)] (**Mn4**) (**A** in the calculation in the form of **Mn4**·THF).

**Scheme 4:**
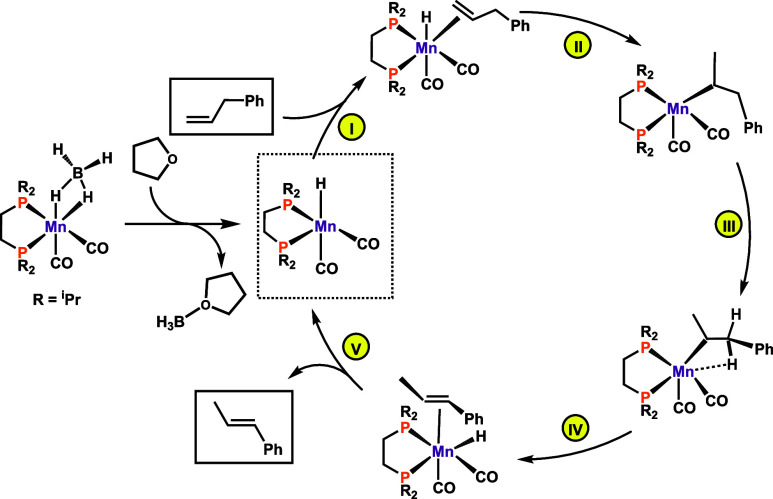
Simplified Catalytic Cycle for the Isomerization of Allylbenzene in THF

The reaction mechanism was explored in detail by means of DFT calculations,^20^ with allylbenzene taken as a model substrate and **Mn4** (**A** in the calculations as a THF adduct) as a precatalyst in THF as a solvent. The resulting free energy profiles are presented in Figures 2–4, while Scheme 4 depicts a summary of the catalytic cycle. The free energy profile for the initiation process, where the active catalyst is formed, is depicted in Figure 2. The first step is the addition of a THF molecule in **A** to the B-atom of the BH_4_ ligand, thereby forming **B** containing a κ^1^-H-coordinated BH_3_·THF adduct. This first step requires a high barrier of 22 kcal/mol and is endergonic with Δ*G* = 18 kcal/mol. This is in line with in situ NMR spectroscopy during catalysis, where such a species could not be detected. The dominant species throughout the reaction was found to be **Mn4** even upon full conversion. Dissociation of BH_3_·THF affords the catalytically active species **C** ([Mn(dippe)(CO)_2_H]) in an endergonic step (Δ*G* = 7 kcal/mol) with a barrier of 8 kcal/mol (see also Scheme 4).

Addition of allylbenzene affords complex **D** bearing a η^2^-bound CH_2_=CHCH_2_Ph ligand. In the next step of the reaction, the hydride migrates to the terminal olefin C atom, resulting in an alkyl complex **E** stabilized by a C–H agostic interaction with the terminal C–H bond (Figure 3). This is a very facile step with a barrier of merely 5 kcal/mol and a free energy balance of Δ*G* = 1 kcal/mol. In the following steps, the agostic interaction is cleaved to yield **F**, which is then further stabilized by forming an agostic interaction with the C–H bond adjacent to the phenyl substituent to give intermediate **G**. These processes are essentially thermoneutral and have a barrier of 11 kcal/mol.

Complex **G** undergoes β-elimination to form complex **H** featuring the η^2^-coordinated product olefin (Figure 4). The barrier is only 7 kcal/mol with a free energy balance of Δ*G* = 7 kcal/mol with respect to **G**. In the last step of the mechanism, *E*-CH_3_CH=CHPh (**2a**) is liberated from **H** to yield the coordinatively unsaturated hydride complex **J**. This complex undergoes facile isomerization to afford the catalytically active species **K**, thereby closing the catalytic cycle (see Scheme 4). It has to be noted that this intermediate is essentially identical to **C** but without the loosely bound BH_3_·THF adduct obtained in the initiating step (Figure 2). Closing the cycle from **K** back to **D** with the addition of a fresh allylbenzene molecule [**K** + CH_3_CH=CHPh (**2a**) → **D**] has a free energy balance of Δ*G* = −5 kcal/mol. In summary, initiation is an endergonic (Δ*G* = 19 kcal/mol) process with a barrier of 26 kcal/mol, from **A** to **TS**_**BC**_, while the catalytic cycle has a barrier of Δ*G*^⧧^ = 11 kcal/mol, from **E** to **TS**_**EF**_.

Formation of the *Z*-isomer of the product was also addressed by DFT mechanistic studies (see Supporting Information). The process involves a C–H agostic exchange in intermediate **G**, followed by β-elimination and the loss of the corresponding product *Z*-CH_3_CH=CHPh. The process has a barrier of 14 kcal/mol measured from **G** to the β-hydride elimination transition state **TS**_**LM**_, which is thus 4 kcal/mol less favorable than the formation of the *E* isomer. This is in good agreement with the experimental observations.

## Conclusions

In sum, we have introduced an efficient protocol for the transposition of terminal alkenes catalyzed by the Mn(I) borohydride complex *fac*-[Mn(dippe)(CO)_2_(κ^2^-BH_4_)]. The reported procedure operates at room temperature with a catalyst loading of 2.5 mol % and no additives. High reactivities and excellent *E*-selectivities were achieved for the allylbenzene derivatives. While maintaining good reactivity, the selectivity was slightly diminished for aliphatic systems. Increasing the steric bulk in these substrates re-established high *E*-selectivity. The protocol was also applied for gram-scale synthesis with 4-allyl-1,1′-biphenyl. Preliminary studies also showed a temperature-dependent chain-walking process leading to a sequential migration of the double bond. Deuterium labeling studies were carried out to gain insights into the reaction mechanism. The influence of additives such as pyridine and PMe_3_ on the reactivity of *fac*-[Mn(dippe)(CO)_2_(κ^2^-BH_4_)] was investigated, ultimately leading to the detection of hydride species. Both additives lead to deactivation by the strongly coordinating ligands, being in line with the requirement of a vacant coordination site during catalysis. DFT calculations indeed disclosed a typical inner sphere mechanism with all reacting fragments coordinated to the metal. The reaction proceeds via a metal hydride mechanism involving hydride insertion of the terminal C=C bond of the olefin, resulting in an alkyl complex. Consecutive β-elimination of the internal C–H bond adjacent to the substituent yields the isomerization product. Notably, formation of the *E*-isomer is favored both kinetically and thermodynamically.

## Materials and Methods

### General Procedure for Catalytic Reactions

Inside an argon flushed glovebox, an NMR tube was charged with a solution of **Mn4** (0.0049 g, 0.013 mmol, 2.5 mol %) in THF-*d*_8_ (0.500 mL) and 1,4-dioxane (0.011 mL, 0.13 mmol) as an internal standard. The alkene substrate (0.50 mmol) was added, and the NMR tube was left to stand at room temperature. Experiments at elevated temperatures were conducted by placing the NMR tube in a preheated oil bath. The progress of the reaction was monitored by ^1^H NMR. After the indicated time, the reaction was quenched by exposure to air. GC–MS analysis was performed using butylbenzene as an internal standard. Isolated products were obtained by filtration over silica in PE and careful evaporation of the volatiles.

## Supporting Information Available

Synthetic procedures; ^1^H, ^13^C{^1^H}, and ^31^P{H} NMR spectra of all compounds; and complete computational details (PDF)
Cartesian coordinates for DFT-optimized structures (XYZ)

## Supplementary Material

cs4c03364_si_001.pdf

cs4c03364_si_002.xyz
