# Supplementary material for: Alkene Isomerization Catalyzed by a Mn(I) Bisphosphine Borohydride Complex
Source: ACS Catal. 2024 Aug 17;14(17):13174–80. doi: 10.1021/acscatal.4c03364 (PMC11385370; doi:10.1021/acscatal.4c03364)
Supplement: Supplementary file 1 — cs4c03364_si_001.pdf [file cs4c03364_si_001.pdf]

Alkene Isomerization Catalyzed by a Mn(I) Bisphosphine Borohydride Complex

Ines Blaha,[†] Stefan Weber,[*,†] Robin Dülger,[†] Luis F. Veiros,[$] Karl Kirchner[*,†]

[†] Institute of Applied Synthetic Chemistry, TU Wien, Getreidemarkt 9/163-AC, A-1060 Wien, Austria.
[$] Centro de Química Estrutural, Institute of Molecular Sciences, Departamento de Engenharia Química, Instituto Superior Técnico, Universidade de Lisboa, Av. Rovisco Pais, 1049 001 Lisboa, Portugal

*Corresponding authors: karl.kirchner@tuwien.ac.at, stefan.e163.weber@tuwien.ac.at

Supporting Information

Table of Content

1 Experimental Section 2
2 Characterization of Organic Products 9
3 Mechanistic Studies 14
4 Computational Details 21
5 Spectra 23
6 References 57

1 Experimental Section

1.1 General Information

All manipulations were performed under an inert atmosphere of argon using standard Schlenk techniques or an MBraun glovebox. The solvents were purified according to standard procedures. Complexes *fac*-[Mn(dippe)(CO)$_3$(CH$_2$CH$_2$CH$_3$)] (dippe = 1,2-bis(di-*iso*-propylphosphino)ethane) (**Mn1**), *fac*-[Mn(dippe)(CO)$_3$(H)] (**Mn2**) and *cis*-[Mn(dippe)(CO)$_2$(κ^2-HBpin)] (**Mn3**) were synthesized according to literature procedure.[1–3] Non commercially available allyl substrates were obtained by modified literature procedures.[4–8] Deuterated solvents were purchased from Eurisotop and dried over 3 Å molecular sieves. Allylic substrates were obtained from commercial sources or prepared according to modified literature procedures. ^{1}H, ^{11}B, ^{13}C{^{1}H}, ^{19}F and ^{31}P{^{1}H} NMR spectra were recorded on Bruker AVANCE-250, AVANCE-400 or AVANCE-600 spectrometers. ^{1}H and ^{13}C{^{1}H} NMR spectra were referenced internally to residual protio-solvent and solvent resonances are reported relative to tetramethylsilane (δ = 0 ppm). ^{31}P{^{1}H} and ^{11}B NMR spectra were referenced externally to H_3PO_4 (85%) (δ = 0 ppm) and $BF_3 \cdot Et_2O$ (15% in $CDCl_3$) (δ = 0 ppm), respectively.

GC-MS analysis was conducted on a ISQ LT Single Quadrupole MS (Thermo Fisher) directly interfaced to a TRACE 1300 Gas Chromatographic system (Thermo Fisher), using a Rxi-5Sil MS (30 m, 0.25 mm ID) cross-bonded dimethyl Polysiloxane capillary column.

High-resolution accurate mass spectra were recorded on an Agilent 6545 QTOF equipped with an Agilent MMI ion source (Agilent Technologies, Santa Clara, CA, USA) which can be operated in mixed ESI and APCI mode. Measured accurate mass data for confirming calculated elemental compositions were within ±3 ppm accuracy.

1.2 General Procedure for Catalytic Reactions

Monoisomerization Reactions

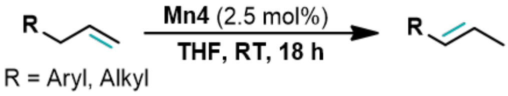

Inside an argon flushed glovebox an NMR tube was charged with solution of **Mn4** (0.0049 g, 0.013 mmol, 2.5 mol%) in THF-*d$_8$* (0.500 mL) and dioxane (0.011 mL, 0.13 mmol) as internal standard. The alkene substrate (0.50 mmol) was added and the NMR tube was left to stand at room temperature. Experiments at elevated temperatures were conducted by placing the NMR tube in a preheated oil bath. The progress of the reaction was monitored by ^{1}H NMR and upon completion the reaction was quenched by exposure to air. GC-MS analysis was performed using butylbenzene as internal standard. Isolated products were obtained by filtration over silica in PE and careful evaporation of the volatiles. *Note:* For all catalytic experiments < 5 % of reduced alkane product was detected.

Consecutive Chain-Walking Experiments

For the consecutive chain-walking experiments the foregoing monoisomerization was first performed as described above. Instead of quenching the reaction by exposure to air, the solution was passed through a syringe filter inside the glovebox. A fresh batch of complex **Mn4** (0.0049 g, 0.013 mmol) in THF-*d$_8$* (0.150 mL) was added. The NMR tube was placed in an oil

bath that was preheated to 70 °C. After 18 h the sample was again analyzed by NMR. After quenching by exposure to air, GC-MS analysis was performed.

Optimizations

Inside an argon flushed glovebox an NMR tube was charged with solution of **Mn4** (0.0049 g, 0.013 mmol, 2.5 mol%) in the respective solvent (0.500 mL) and dioxane (0.011 mL, 0.13 mmol) as internal standard. The alkene substrate (0.50 mmol) was added and the NMR tube was left to stand at room temperature. Experiments at elevated temperatures were conducted by placing the NMR tube in a preheated oil bath. Conversion and *E*/*Z* ratio were determined by GC-MS and/or NMR spectroscopy (^{1}H, ^{19}F).

1.3 Synthesis of Non-Commercial Reagents

Synthesis of Complexes

Synthesis of *cis*-[Mn(dippe)(CO)$_2$(κ^2-BH$_4$)] (Mn4). Inside an argon-flushed glovebox a Schlenk flask was charged with NH_3BH_3 (0.167 g, 5.40 mmol, 24 equiv.). **Mn1**[1] (0.100 g, 0.23 mmol) was dissolved in THF (7 mL) and transferred into the Schlenk flask. The flask was heated in an oil bath at 50 °C and was constantly allowed to ventilate during heating. After 3.5 h the reaction mixture was taken to dryness and the residual orange solid was extracted with a mixture of toluene and pentane (1:1, 7 x 4 mL). The solvent was removed in vacuo and the crude product was washed with pentane (2 mL) to obtain a bright orange powder (0.069 g, 79 %). ^{1}H NMR (600 MHz, C_6D_6) δ 6.01 (br, 1H, terminal B-H), 5.08 (br, 1H, terminal B-H), 2.44 (br, 1H), 2.08 (br, 1H), 1.79 (br, 1H), 1.58 – 1.47 (m, 2H), 1.38 – 1.17 (m, 4H), 1.11 – 1.04 (m, 6H), 0.92 – 0.77 (m, 17 H), -11.35 (br, 1H, Mn-H), -12.98 (br, 1H, Mn-H). $^{13}C\{^{1}H\}$ NMR (151 MHz, C_6D_6) δ 29.5 (d, $J_{C\text{-}P}$ = 24.8 Hz), 27.5 (d, $J_{C\text{-}P}$ = 19.3 Hz), 26.8 (d, $J_{C\text{-}P}$ = 20.0 Hz), 24.6 (dd, $J_{C\text{-}P}$ = 18.4, 14.7 Hz), 21.9 (d, $J_{C\text{-}P}$ = 11.4 Hz), 21.0 (dd, $J_{C\text{-}P}$ = 20.2, 14.0 Hz), 20.6, 19.1, 19.1, 19.1, 19.0, 18.8, 18.6, 17.7 (CO not observed). $^{31}P\{^{1}H\}$ NMR (243 MHz,C_6D_6) δ 121.8, 86.8. $^{11}B\{^{1}H\}$ NMR (128 MHz, C_6D_6) δ 28.0. ATR-IR (cm^{-1}): 1944 (ν_{CO}), 1868 (ν_{CO}). HRMS (TOF ESI+): *m/z* calculated for $C_{16}H_{36}BMnNaO_2P_2$ $[M+Na]^+$: 411.1556, found 411.1560. *Note: Due to long-term thermal instability, no elemental analysis was conducted.*

Synthesis of *cis*-[Mn(dippe)(CO)$_2$(pyr)H] (Mn5). Inside an argon flushed glove box an 8 mL glass vial was charged with a solution of **Mn4** (0.010 g, 0.03 mmol, 1 equiv.) in C_6D_6 (1 mL). Pyridine (0.014 mL, 0.17 mmol, 5 equiv.) was added and the orange solution was stirred for 15 minutes before being transferred into an NMR tube for analysis. ^{1}H NMR (600 MHz, C_6D_6) δ 9.17 (d, *J* = 5.2 Hz, 2H, pyr(2,6)), 8.14 (d, *J* = 4.8 Hz, 1H, pyr(4)), 6.58 – 6.50 (m, 2H, pyr(3,5)), 2.04 (m, 2H), 1.87 (hept, *J* = 8.1 Hz, 2H), 1.45 (dd, *J* = 13.7, 7.0 Hz, 3H), 1.32 (dd, *J* = 14.0, 7.1 Hz, 5H), 1.22 (ddd, *J* = 25.1, 12.5, 7.0 Hz, 7H), 1.14 – 1.05 (m, 6H), 0.92 (dd, *J* = 10.8, 7.0 Hz, 4H), 0.80 (dd, *J* = 13.1, 7.1 Hz, 3H), -4.52 (m, 1H). $^{13}C\{^{1}H\}$ NMR (151 MHz, C_6D_6) δ 158.7, 147.3, 122.5, 31.2 (d, $J_{C\text{-}P}$ = 19.8 Hz), 28.4 (d, $J_{C\text{-}P}$ = 10.0 Hz) 28.1 (d, $J_{C\text{-}P}$ = 23.4 Hz), 24.8 (m), 21.5 (dd, $J_{C\text{-}P}$ = 21.8, 15.4 Hz), 20.3, 19.4 ($J_{C\text{-}P}$ = 2.6 Hz), 19.0, 18.9, 18.8 (d, $J_{C\text{-}P}$ = 4.5 Hz), 18.7 (d, $J_{C\text{-}P}$ = 3.0 Hz), 18.5, 18.4 (CO not observed). $^{31}P\{^{1}H\}$ NMR (243 MHz, C_6D_6) δ 128.5, 97.4. ATR-IR (cm^{-1}): 1881 (ν_{CO}), 1807 (ν_{CO}). HRMS (TOF ESI+): *m/z* calculated for $C_{21}H_{38}MnNO_2P_2$ $[M]^+$: 453.1752, found 453.1756. *Note: Due to long-term thermal instability, no elemental analysis was conducted.*

Synthesis of *cis*-[Mn(dippe)(CO)$_2$(PMe$_3$)H] (Mn6). Inside an argon flushed glovebox an 8 mL glass vial was charged with a solution of **Mn4** (0.010 g, 0.03 mmol, 1 equiv.) in toluene (1 mL). Addition of PMe$_3$ (0.013 mL, 0.13 mmol, 5 equiv.) led to instant decoloring of the clear, orange solution. The reaction was stirred for 10 minutes before all volatiles were removed in vacuo. The crude product was washed with cold pentane (0.5 mL) to yield a white solid (0.010 g, 87 %). ^{1}H NMR (600 MHz, C$_6$D$_6$) δ 2.12 (hept, J = 7.5 Hz, 2H), 1.96 – 1.87 (m, 1H), 1.69 – 1.63 (m, 1H), 1.39 (d, J = 7.3 Hz, 9H), 1.37 – 1.31 (m, 4H), 1.24 (ddd, J = 19.9, 13.9, 7.0 Hz, 8H), 1.12 – 0.99 (m, 10H), 0.98 – 0.93 (m, 3H), 0.91 – 0.84 (m, 3H), -9.00 (m, 1H). ^{13}C{^{1}H} NMR (151 MHz, C$_6$D$_6$) δ 30.6 (d, $J_{C\text{-}P}$ = 20.0 Hz), 28.7 (d, $J_{C\text{-}P}$ = 12.7 Hz), 28.2 (d, $J_{C\text{-}P}$ = 23.6 Hz), 26.7 (d, $J_{C\text{-}P}$ = 17.3), 24.5 (d, $J_{C\text{-}P}$ = 24.5 Hz), 23.1 (m), 22.3 (m), 19.9, 19.9, 19.4 (d, $J_{C\text{-}P}$ = 6.6 Hz), 19.0, 18.9, 18.7, 18.5, 17.7 (d, $J_{C\text{-}P}$ = 5.4 Hz) (CO not observed). ^{31}P{^{1}H} NMR (243 MHz, C$_6$D$_6$) δ 126.4, 110.2, 28.5. ATR-IR (cm^{-1}): 1889 (ν_{CO}), 1828 (ν_{CO}). HRMS (TOF ESI+): *m/z* calculated for C$_{19}$H$_{42}$MnO$_2$P$_3$ [M]$^+$: 450.1772, found 450.1775. *Note: Due to long-term thermal instability, no elemental analysis was conducted.*

Synthesis of Substrates

Grignard Reactions

Allylbenzene analogues were synthesized according to modified literature procedure.[4]

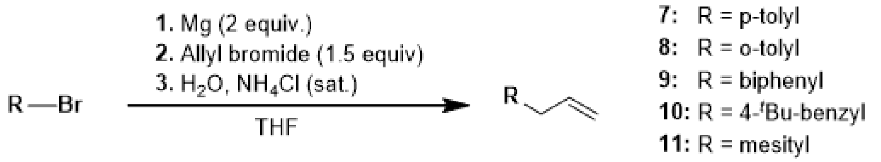

Mg (2.0 equiv) was placed in a flame-dried 250 mL three-necked flask equipped with a reflux condenser and a dropping funnel. The turnings were layered with anhydrous THF (10 mL) and a portion of the respective aryl bromide (approx. 0.33 equiv) was added under stirring. When a color change indicated the reaction onset, a solution of the remaining aryl bromide (0.66 equiv) in THF (40 mL) was added slowly via dropping funnel. After complete addition the dropping funnel was flushed with THF (10 mL) and the reaction was heated to reflux (1 – 3 hours). It was left to cool before the solution was decanted into a Schlenk flask and cooled in an ice bath. Allyl bromide (1.5 equiv) was added slowly via septum. The solution was allowed to warm to room temperature and subsequently stirred at room temperature overnight. The reaction was quenched with water and saturated aqueous NH$_4$Cl solution. The phases were separated and the aqueous phase was extracted with Et$_2$O (6 x 60 mL). The combined organic phases were dried over Na$_2$SO$_4$ and the solvent was removed in vacuo. Purification details and characterization are given individually for each substrate.

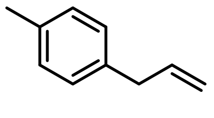

1-Allyl-4-methylbenzene (7). This compound was synthesized following the protocol described above using 4-bromo toluene (6.15 g, 36.0 mmol). The product was purified by distillation (bp 96 °C at 60 mbar) and obtained as colorless oil (2.63 g, 55 %). NMR data matches values previously reported.[4]

^{1}H NMR (250 MHz, CDCl$_3$) δ 7.22 – 7.04 (m, 4H), 6.02 (ddt, J = 13.5, 10.0, 6.8 Hz, 1H), 5.21 – 5.00 (m, 2H), 3.38 (d, J = 6.7 Hz, 2H), 2.34 (s, 3H).

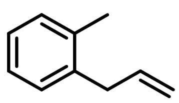

1-Allyl-2-methylbenzene (8). This compound was synthesized following the protocol described above using 2-bromo toluene (5.00 g, 29.2 mmol). The product was purified by distillation (bp 94 °C at 60 mbar) and obtained as colorless oil (2.23 g, 58 %). NMR data matches values previously reported.[7]

^{1}H NMR (400 MHz, CD_3CN) δ 7.14 (d, *J* = 1.2 Hz, 4H), 5.97 (ddt, *J* = 16.6, 10.2, 6.4 Hz, 1H), 5.15 – 4.91 (m, 2H), 3.38 (dt, *J* = 6.4, 1.4 Hz, 2H), 2.28 (s, 3H).

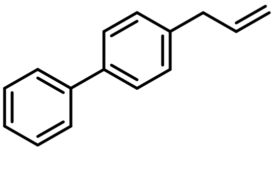

4-Allyl-1,1'-biphenyl (9). This compound was synthesized following the protocol described above using 4-bromo biphenyl (9.80 g, 42.0 mmol). The product was purified by column chromatography (silica, eluent: PE/Et_2O = 9:1) and obtained as white solid (4.35 g, 53 %). NMR data matches values previously reported.[9]

^{1}H NMR (250 MHz, DMSO-d_6) δ 7.71 – 7.54 (m, 4H), 7.50 – 7.14 (m, 5H), 5.98 (ddt, *J* = 16.8, 9.9, 6.8 Hz, 1H), 5.19 – 5.00 (m, 2H), 3.39 (d, *J* = 6.7 Hz, 2H).

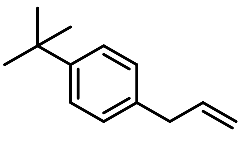

1-Allyl-4-(^{t}Bu)benzene (10). This compound was synthesized following the protocol described above using 1-bromo-4-(^{t}Bu)benzene (6.39 g, 30.0 mmol). The product was purified by distillation (bp 55 °C at 1 mbar) and obtained as clear, colorless liquid (3.13 g, 60 %). NMR data matches values previously reported.[10]

^{1}H NMR (400 MHz, CD_3CN) δ 7.40 – 7.31 (m, 2H), 7.18 – 7.10 (m, 2H), 5.98 (ddt, *J* = 16.9, 10.0, 6.8 Hz, 1H), 5.10 (dq, *J* = 17.0, 2.1 Hz, 1H), 5.04 (ddt, *J* = 10.0, 2.2, 1.2 Hz, 1H),, 3.36 (d, *J* = 6.9 Hz, 2H), 1.31 (d, J = 2.5 Hz, 9H).

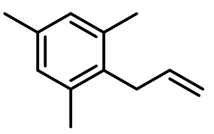

2-Allyl-1,3,5-trimethylbenzene (11). This compound was synthesized following the protocol described above using 2-bromo mesitylene (6.00 g, 30.9 mmol). The product was purified by distillation (bp 129 °C at 60 mbar) and obtained as clear, colorless liquid (3.71 g, 77 %). NMR data matches values previously reported.[7]

^{1}H NMR (600 MHz, CD_3CN) δ 6.84 (s, 2H), 5.91 (ddt, *J* = 17.1, 10.1, 5.8 Hz, 1H), 4.97 (dq, *J* = 10.1, 1.8 Hz, 1H), 4.85 (dq, *J* = 17.1, 1.9 Hz, 1H), 3.36 (dt, *J* = 5.7, 1.7 Hz, 2H), 2.23 (d, *J* = 4.6 Hz, 9H).

1-Allyladamantane (20). This compound was synthesized following a modified literature procedure.[5]

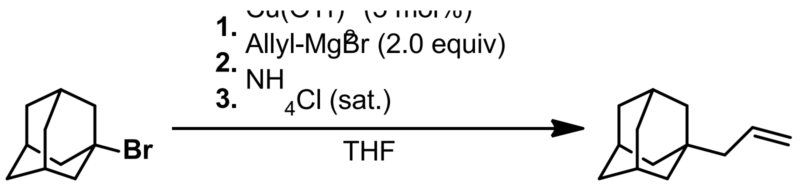

In a Schlenk flask $Cu(OTf)_2$ (0.136 g, 0.4 mmol, 5 mol%) was dissolved in anhydrous THF (25 mL). A solution of 1-bromoadamantane (1.61 g, 7.5 mmol, 1 equiv.) in THF (8 mL) was added and the flask was cooled in an ice bath. Allylmagnesium bromide (1.0 M solution in Et_2O, 15.0 mmol, 2 equiv.) was added dropwise under cooling. After the addition was completed, the formed grey suspension was stirred at room temperature for 3 h. The reaction was quenched by pouring it onto a saturated aqueous NH_4Cl solution (70 mL). The clear,

biphasic mixture was separated and the aqueous layer was extracted with PE (4 x 70 mL). The combined organic phases were dried over Na_2SO_4 and concentrated in vacuo. The product was purified by column chromatography (silica, eluent: PE) and obtained as a colorless oil (0.722 g, 79 %). NMR data matches values previously reported.[11]
^{1}H NMR (400 MHz, $CDCl_3$) δ 5.83 (ddt, *J* = 17.6, 10.2, 7.5 Hz, 1H), 5.07 – 4.92 (m, 2H), 1.95 (m, 3H), 1.83 (d, *J* = 7.5 Hz, 2H), 1.80 – 1.56 (m, 6H), 1.49 (d, *J* = 2.5 Hz, 6H).

Trimethylsilyl Ether Protection of Alcohols

Trimethylsilyl ethers were synthesized according to modified literature procedure.[6]

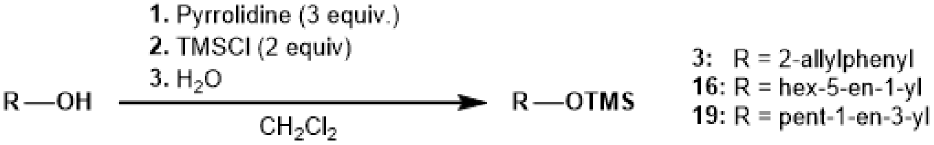

In a Schlenk flask the alcohol (1 equiv.) was dissolved in anhydrous CH_2Cl_2 (5 mL). Pyrrolidine (3 equiv.) was added and the clear solution was stirred at room temperature for 15 minutes. TMSCl (2 equiv.) was added dropwise *via* septum and the mixture was stirred at room temperature for 20 h. The reaction was quenched with water (5 mL), the phases were separated and the aqueous phase was extracted with CH_2Cl_2 (4 x 5 mL). The combined organic phases were dried over Na_2SO_4 and concentrated in vacuo. Purification details and characterization are given individually for each substrate.

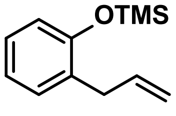

(2-Allylphenoxy)trimethylsilane (3). This compound was synthesized following the protocol described above using 2-allylphenol (0.46 g, 3.5 mmol). The product was purified by column chromatography (silica, eluent: PE/Et_2O = 4:1) and obtained as colorless liquid (0.25 g, 35 %). NMR data matches values previously reported.[12]
^{1}H NMR (250 MHz, DMSO-d_6) δ 7.11 (t, *J* = 7.3 Hz, 2H), 6.96 – 6.76 (m, 2H), 6.04 – 5.77 (m, 1H), 5.09 – 4.94 (m, 2H), 3.28 (d, *J* = 6.6 Hz, 2H), 0.24 (s, 9H).

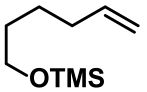

(Hex-5-en-1-yloxy)trimethylsilane (16). This compound was synthesized following the protocol described above using hex-5-en-1-ol (1.74 g, 17.4 mmol). The product was purified by column chromatography (silica, eluent: PE/EE = 3:1) and obtained as colorless liquid (0.34 g, 11 %). NMR data matches values previously reported.[13]
^{1}H NMR (600 MHz, C_6D_6) δ 5.75 (ddt, *J* = 16.9, 10.2, 6.7 Hz, 1H), 5.01 (dq, *J* = 17.1, 1.7 Hz, 1H), 4.97 (ddt, J = 10.1, 2.3, 1.3 Hz, 1H), 3.48 (t, J = 6.3 Hz, 2H), 2.00 – 1.93 (m, 2H), 1.49 (dt, *J* = 8.9, 6.3 Hz, 2H), 1.43 – 1.36 (m, 2H), 0.10 (s, 9H).

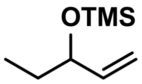

Trimethyl(pent-1-en-3-yloxy)silane (19). This compound was synthesized following the protocol described above using 1-penten-3-ol (0.45 g, 5.2 mmol). The product was purified by column chromatography (silica, eluent: PE/Et_2O = 4:1) and obtained as colorless liquid (0.29 g, 34 %). NMR data matches values previously reported.[14]
^{1}H NMR (250 MHz, DMSO-d_6) δ 5.75 (dd, *J* = 10.4, 5.9 Hz, 1H), 5.13 (dt, *J* = 16.9, 1.5 Hz, 1H), 5.02 (dt, *J* = 10.6, 1.6 Hz, 1H), 4.10 – 3.95 (m, 1H), 1.41 (pd, *J* = 7.3, 2.7 Hz, 2H), 0.81 (t, *J* = 7.4 Hz, 3H), 0.07 (s, 9H).

Wittig Reactions

Wittig reactions were performed according to literature procedure.[7]

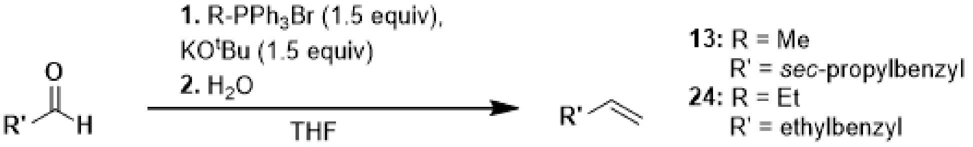

In a flame-dried three-necked 250 mL flask the Wittig salt ($MePPh_3Br$ or $EtPPh_3Br$, 1.5 equiv.) was suspended in anhydrous THF (60 mL). Under ice bath cooling pre-dried KO^tBu (1.5 equiv.) was added and the bright yellow/orange suspension was stirred for 30 minutes. Then, the aldehyde (1 equiv.) was added and the decolored suspension was left to thaw. The reaction was stirred for 18 h and monitored by TLC. Upon completion it was quenched with water, the phases were separated and the aqueous phase was extracted with Et_2O (4 x 60 mL). The combined organic phases were dried over Na_2SO_4 and concentrated in vacuo. Purification details and characterization are given individually for each substrate.

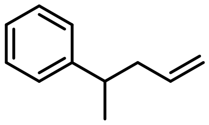

Pent-4-en-2-ylbenzene (13). This compound was synthesized following the protocol described above using 3-phenylbutanal (4.45 g, 30 mmol, 1 equiv.). The product was purified by distillation (bp 110 °C at 90 mbar) and obtained as colorless liquid (1.13 g, 26 %). NMR data matches values previously reported.[15]
^{1}H NMR (400 MHz, THF-*d*$_8$) δ 7.39 – 6.99 (m, 5H), 5.70 (ddt, *J* = 17.0, 10.1, 7.0 Hz, 0H), 4.97 (dq, *J* = 17.1, 1.7 Hz, 1H), 4.92 (d, *J* = 10.1 Hz, 0H), 2.76 (h, *J* = 7.0 Hz, 0H), 2.42 – 2.33 (m, 1H), 2.32 – 2.22 (m, 1H), 1.23 (d, *J* = 7.0 Hz, 3H).

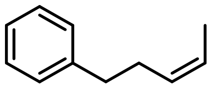

(*Z*)-pent-3-en-1-ylbenzene (24). This compound was synthesized following the protocol described above using hydrocinnamaldehyde (4.03 g, 30.0 mmol). The product was purified by distillation (bp 121 °C at 70 mbar) and obtained as colorless liquid (0.97 g, 22 %). It was obtained as a mixture of isomers (*E*/*Z* = 13:87). NMR data is reported for the main isomer and matches values previously reported.[7]
^{1}H NMR (250 MHz, CD_3CN) δ 7.37 – 7.12 (m, 5H), 5.58 – 5.34 (m, 2H), 2.67 (t, *J* = 7.6 Hz, 2H), 2.48 – 2.25 (m, 2H), 1.56 (dt, *J* = 5.0, 0.9 Hz, 3H).

Miscellaneous

2-Allylfuran

The synthesis of 2-allylfuran **(15)** was performed according to literature procedure.[8]

*Allylbenzene-2,2-d_2 (**2-d_2**)*

The synthesis of **2-d_2** was performed according to literature procedure.[16]

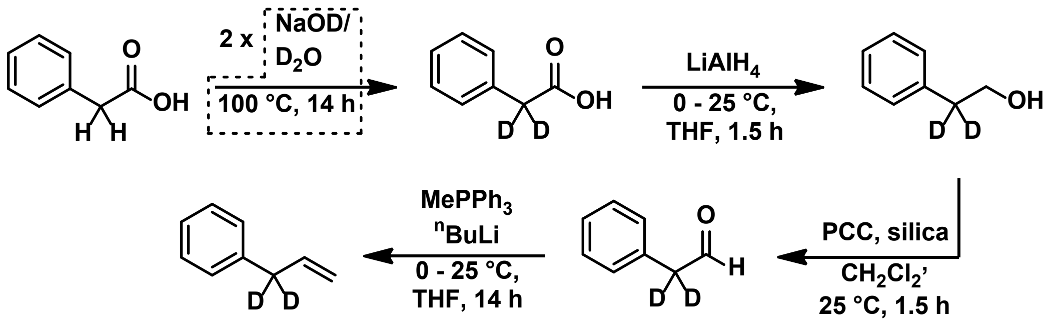

In a 20 mL microwave vial equipped with a stirring bar, phenylacetic acid (1.70 g, 12.5 mmol, 1.0 equiv.) was suspended in D_2O (2.5 mL). NaOD (30 w% in D_2O, 2.5 mL) was added, the vial was sealed and heated to 100 °C overnight. After cooling to room temperature the vial was opened, the content was acidified with 6 M HCl and extracted with CH_2Cl_2 (4 x 10 mL). The combined organic phases were dried over Na_2SO_4 and the solvent was removed in vacuo to yield a white solid. The procedure was repeated once to obtain phenylacetic acid-2,2-d_2 (1.61 g, 93 %) with a deuteration grade of 91 %. ^1H NMR (250 MHz, $CDCl_3$) δ 11.72 (br, 1H), 7.43 – 7.22 (m, 5H). ^2H NMR (38 MHz, $CHCl_3$) δ 3.62.

In a 250 mL three-necked flask $LiAlH_4$ (1.09 g, 28.7 mmol, 1.2 equiv.) was suspended in anhydrous THF (40 mL) and cooled in an ice bath. A solution of phenylacetic acid-2,2-d_2 (3.31 g, 24.0 mmol, 1.0 equiv.) in anhydrous THF (30 mL) was added dropwise. After complete addition the suspension was left to thaw and stirred at room temperature for 1 h. Excess $LiAlH_4$ was quenched with MeOH and water and the suspension was filtered to remove Li and Al salts. The salts were thoroughly washed with EtOAc and the aqueous filtrate was extracted with more EtOAc (3 x 50 mL). The combined organic phases were washed with brine (3 x 25 mL) and dried over Na_2SO_4. The solvent was removed in vacuo to yield phenylethanol-2,2-d_2 as pale yellow liquid (2.64 g, 89 %). ^1H NMR (250 MHz, $CDCl_3$) δ 7.42 – 7.15 (m, 5H), 3.82 (s, 2H), 1.74 (s, 1H). ^2H NMR (38 MHz, $CDCl_3$) δ 2.86.

In a Schlenk flask PCC (4.61 g, 21.4 mmol, 2.1 equiv.) was kept under vacuum for 10 minutes before pre-dried silica (7.0 g) and anhydrous CH_2Cl_2 (50 mL) was added. Phenylethanol-2,2-d_2 (1.27 g, 10.2 mmol, 1 equiv.) was added and the dark brown suspension was stirred for 1.5 h at room temperature. Et_2O (80 mL) was added and the mixture was filtered through a pad of pre-dried silica. Crude phenylacetaldehyde-2,2-d_2 was obtained as a viscous, pale yellow liquid (1.03 g, 83 %) that was used for the next step without further purification. ^1H NMR (250 MHz, $CDCl_3$) δ 9.76 (s, 1H), 7.55 – 6.97 (m, 5H). ^2H NMR (38 MHz, $CDCl_3$) δ 3.68.

In a flame-dried Schlenk flask $MePPh_3Br$ (2.78 g, 7.77 mmol, 1.2 equiv.) was suspended in anhydrous THF (15 mL) and cooled in an ice bath. ^nBuLi (1.6 M in hexane, 9.33 mmol, 1.4

equiv.) was added and the bright red solution was stirred for 20 minutes under cooling before phenylacetaldehyde-2,2-d_2 (0.79 g, 6.48 mmol, 1.0 equiv.) was added. After cooling for another 30 minutes the reaction was left to thaw and stirred at room temperature overnight. The pale yellow reaction was quenched by exposure to air and subsequently water and extracted with Et_2O (4 x 30 mL). The combined organic phases were dried over Na_2SO_4 and the total volume was reduced to about a third in vacuo. Allylbenzene-2,2-d_2 was obtained as a colorless liquid (0.13 g, 16 %) by distillation under reduced pressure (60 mbar). ^{1}H NMR (250 MHz, THF-d_8) δ 7.29 – 7.08 (m, 5H), 5.94 (dd, J = 17.0, 10.0 Hz, 1H), 5.11 – 4.96 (m, 2H). ^{2}H NMR (38 MHz, THF) δ 3.35.

2 Characterization of Organic Products

Note: Analytic data is reported for the main product of isomerization reactions. Yields and E/Z ratios were determined spectroscopically by ^{1}H NMR using 1,4-dioxane as standard and are given in parenthesis.

2.1 Products of One-Bond Isomerization

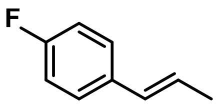

(*E*)-1-Fluoro-4-(prop-1-en-1-yl)benzene (1a) (97 %, E/Z = 99:1). The product was prepared following the general procedure for isomerization reactions described in section 1.2. NMR data matches values previously reported.[17]

^{1}H NMR (400 MHz, THF-d_8) δ 7.36 – 7.25 (m, 2H), 7.03 – 6.91 (m, 2H), 6.36 (dd, J = 15.8, 1.5 Hz, 1H), 6.16 (dq, J = 15.7, 6.6 Hz, 1H), 1.82 (dd, J = 6.6, 1.6 Hz, 2H). ^{19}F NMR (377 MHz, THF-d_8) δ -117.05.

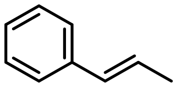

(*E*)-Prop-1-en-1-ylbenzene (2a) (97 %, E/Z = 99:1). The product was prepared following the general procedure for isomerization reactions described in section 1.2. NMR data matches values previously reported.[18]

^{1}H NMR (400 MHz, THF-d_8) δ 7.30 (d, J = 7.4 Hz, 2H), 7.23 (t, J = 7.6 Hz, 2H), 7.12 (t, J = 7.3 Hz, 1H), 6.46 – 6.34 (m, 1H), 6.23 (dq, J = 15.7, 6.5 Hz, 1H), 1.84 (dd, J = 6.5, 1.6 Hz, 3H).

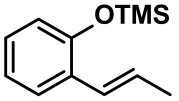

(*E*)-(2-Allylphenoxy)trimethylsilane (3a) (Isolated yield: 98 %, pale yellow oil, E/Z = 99:1). The product was prepared following the general procedure for isomerization reactions described in section 1.2. NMR data matches values previously reported.[16]

^{1}H NMR (600 MHz, $CDCl_3$) δ 7.43 (dd, J = 7.7, 1.5 Hz, 1H), 7.10 (td, J = 7.8, 1.6 Hz, 1H), 6.94 (t, J = 7.3 Hz, 1H), 6.81 (dd, J = 8.0, 0.9 Hz, 1H), 6.67 (dd, J = 15.9, 1.6 Hz, 1H), 6.22 (dd, J = 15.9, 6.6 Hz, 1H), 1.92 (dd, J = 6.6, 1.7 Hz, 3H), 0.30 (s, 9H). ^{13}C{^{1}H} NMR (151 MHz, $CDCl_3$) δ 152.2, 129.5, 127.6, 126.4, 126.3, 125.9, 119.9, 19.0, 0.5.

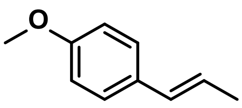

(*E*)-Anethole (4a) (Isolated yield: 93 %, colorless oil, E/Z = 99:1). The product was prepared following the general procedure for isomerization reactions described in section 1.2. NMR data matches values previously reported.[17]

^{1}H NMR (400 MHz, THF-d_8) δ 7.22 (d, J = 8.6 Hz, 2H), 6.80 (d, J = 8.6 Hz, 2H), 6.32 (d, J = 15.7 Hz, 1H), 6.13 – 5.99 (m, 1H), 3.71 (s, 3H), 1.81 (dd, J = 6.7, 1.2 Hz, 3H). ^{13}C{^{1}H} NMR (151 MHz, $CDCl_3$) δ 158.7, 130.9, 130.4, 126.9, 123.5, 114.0, 55.3, 18.5.

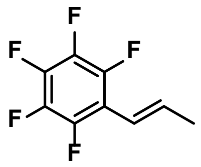

1,2,3,4,5-Pentafluoro-6-(prop-1-en-1-yl)benzene (5a) (Isolated yield: 92 %, pale yellow oil, E/Z = 95:5). The product was prepared following the general procedure for isomerization reactions described in section 1.2. NMR data matches values previously reported.[15]

^{1}H NMR (250 MHz, THF-d_8) δ 6.61 (dq, J = 19.7, 6.4 Hz, 1H), 6.33 (d, J = 16.3 Hz, 1H), 1.96 (d, J = 6.0 Hz, 3H). ^{19}F NMR (565 MHz, $CDCl_3$) δ - 144.2 (dd, J = 21.5, 7.7 Hz), -158.3 (t, J = 20.7 Hz), -163.7 (td, J = 21.3, 7.8 Hz). ^{13}C{^{1}H} NMR (151 MHz, $CDCl_3$) δ 145.5 (ddt, J_{C-F} = 11.8, 8.2, 3.6 Hz), 143.8 (tt, J_{C-F} = 8.2, 4.1 Hz), 140.3 (tt, J_{C-F} = 13.6, 4.5 Hz), 138.8 – 138.4 (m), 137.2 – 136.8 (m), 136.3 – 136.0 (m), 115.5, 112.9 – 112.3 (m).

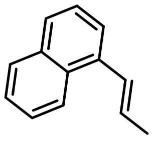

(*E*)-1-(prop-1-en-1-yl)naphthalene (6a) (96 %, E/Z = 90:10). The product was prepared following the general procedure for isomerization reactions described in section 1.2. NMR data matches values previously reported.[7]

^{1}H NMR (400 MHz, THF-d_8) δ 8.13 (d, J = 7.7 Hz, 1H), 7.87 – 7.77 (m, 1H), 7.71 (d, J = 8.2 Hz, 1H), 7.53 (d, J = 7.1 Hz, 1H), 7.44 (td, J = 6.3, 3.1 Hz, 2H), 7.40 – 7.35 (m, 1H), 7.18 (d, J = 15.5 Hz, 1H), 6.31 – 6.18 (m, 1H), 1.96 (dd, J = 6.6, 1.6 Hz, 3H).

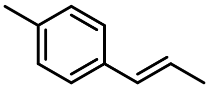

(*E*)-1-Methyl-4-(prop-1-en-1-yl)benzene (7a) (Isolated yield: 95%, colorless oil, E/Z = 98:2). The product was prepared following the general procedure for isomerization reactions described in section 1.2. NMR data matches values previously reported.[7]

^{1}H NMR (600 MHz, THF-d_8) δ 7.19 (d, J = 8.0 Hz, 2H), 7.05 (d, J = 7.9 Hz, 2H), 6.38 – 6.31 (m, 1H), 6.16 (dd, J = 15.7, 6.6 Hz, 1H), 2.27 (s, 3H), 1.83 (dd, J = 6.6, 1.5 Hz, 3H). ^{13}C{^{1}H} NMR (151 MHz, $CDCl_3$) δ 136.5, 135.3, 131.0, 129.3, 125.8, 124.7, 21.2, 18.6.

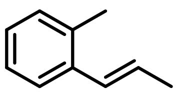

(*E*)-1-Methyl-2-(prop-1-en-1-yl)benzene (8a) (Isolated yield: 97%, colorless oil E/Z = 95:5). The product was prepared following the general procedure for isomerization reactions described in section 1.2. NMR data matches values previously reported.[7]

^{1}H NMR (400 MHz, THF-d_8) δ 7.40 – 7.33 (m, 1H), 7.13 – 6.99 (m, 4H), 6.62 (dd, J = 15.7, 1.4 Hz, 1H), 6.09 (dq, J = 15.7, 6.6 Hz, 1H), 2.29 (s, 3H), 1.87 (dd, J = 6.6, 1.6 Hz, 3H).

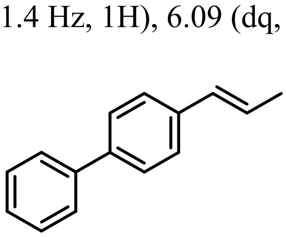

(*E*)-4-(Prop-1-en-1-yl)-1,1'-biphenyl (9a) (Isolated yield: 95 %, white solid, E/Z = 98:2). The product was prepared following the general procedure for isomerization reactions described in section 1.2. NMR data matches values previously reported.[19]

^{1}H NMR (600 MHz, $CDCl_3$) δ 7.55 (dt, J = 8.1, 1.5 Hz, 2H), 7.52 – 7.46 (m, 2H), 7.42 – 7.32 (m, 4H), 7.32 – 7.25 (m, 1H), 6.40 (dd, J = 15.8, 1.6 Hz, 1H), 6.24 (dq, J = 15.7, 6.6 Hz, 1H), 1.86 (dd, J = 6.6, 1.7 Hz, 3H). ^{13}C{^{1}H} NMR (151 MHz, $CDCl_3$) δ 140.9, 139.5, 137.1, 130.7, 128.8, 127.2, 127.2, 127.0, 126.3, 125.9, 18.7.

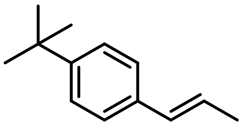

(*E*)-1-(tert-butyl)-4-(prop-1-en-1-yl)benzene (10a) (Isolated yield: 97 %, colorless oil, E/Z = 99:1). The product was prepared following the general procedure for isomerization reactions described in section 1.2. NMR data matches values previously reported.[7]

^{1}H NMR (400 MHz, THF- d_8) δ 7.32 – 7.27 (m, 2H), 7.26 – 7.21 (m, 2H), 6.37 (dd, *J* = 15.7, 1.6 Hz, 1H), 6.18 (dq, *J* = 15.7, 6.5 Hz, 1H), 1.83 (dd, *J* = 6.5, 1.7 Hz, 4H), 1.30 (s, 9H). ^{13}C{^{1}H} NMR (151 MHz, $CDCl_3$) δ 149.8, 135.3, 130.9, 125.7, 125.5, 125.0, 34.6, 31.5, 18.6.

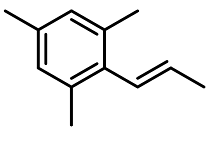

(*E*)-1,3,5-trimethyl-2-(prop-1-en-1-yl)benzene (11a) (99%, E/Z = 61:39). The product was prepared following the general procedure for isomerization reactions described in section 1.2. It was obtained as inseparable mixture of *E*/*Z* isomers. NMR data matches values previously reported.[7]

^{1}H NMR (600 MHz, THF-d_8) δ 6.82 (s, 1H), 6.78 (s, 1H), 6.33 (d, *J* = 16.0 Hz, 1H), 5.64 (dq, *J* = 16.0, 6.5 Hz, 1H), 2.23 (s, 2H), 2.22 (s, 5H), 1.88 (dd, *J* = 6.5, 1.4 Hz, 4H).

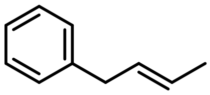

(*E*)-But-2-en-1-ylbenzene (12a) (Isolated yield: 68 %, pale yellow oil, E/Z = 81:19). The product was prepared following the general procedure for isomerization reactions described in section 1.2. It was obtained as a mixture of *E*/*Z* isomers, starting material and chain-walking product **(*E*)-1-Buten-1-ylbenzene (11b)**. NMR data matches values previously reported.[20]

^{1}H NMR (600 MHz, THF-d_8) δ 7.22 (t, *J* = 7.5 Hz, 2H), 7.18 – 7.08 (m, 3H), 5.62 – 5.53 (m, 1H), 5.49 (dd, *J* = 15.1, 6.3 Hz, 1H), 3.29 (d, *J* = 6.6 Hz, 2H), 1.66 (dd, *J* = 6.2, 1.2 Hz, 1H). ^{13}C{^{1}H} NMR (151 MHz, $CDCl_3$) δ 141.2, 130.1, 128.6, 128.4, 126.4, 126.0, 39.2, 18.0.

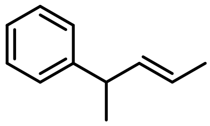

(*E*)-Pent-3-en-2-ylbenzene (13a) (96 %, E/Z = 90:10). The product was prepared following the general procedure for isomerization reactions described in section 1.2. NMR data of the main product matches values previously reported.[15]

^{1}H NMR (400 MHz, THF-d_8) δ 7.27 – 7.19 (m, 2H), 7.19 – 7.15 (m, 2H), 7.11 (tt, *J* = 6.6, 1.7 Hz, 1H), 5.62 (ddq, *J* = 15.2, 6.9, 1.8 Hz, 1H), 5.51 – 5.38 (m, 1H), 3.39 (p, *J* = 6.9 Hz, 1H), 1.65 (d, *J* = 6.3 Hz, 3H), 1.31 (d, *J* = 7.1 Hz, 3H).

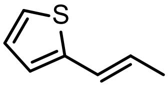

(*E*)-2-(Prop-1-en-1-yl)thiophene (14a) (98 %, *E*/*Z* = 91:9). The product was prepared following the general procedure for isomerization reactions at a temperature of 40 °C described in section 1.2. NMR data matches values previously reported.[21]

^{1}H NMR (400 MHz, THF-d_8) δ 7.11 (d, *J* = 5.0 Hz, 1H), 6.92 – 6.80 (m, 2H), 6.52 (dd, *J* = 15.6, 1.6 Hz, 1H), 6.03 (dq, *J* = 15.5, 6.7 Hz, 1H), 1.80 (dd, *J* = 6.7, 1.8 Hz, 3H).

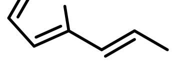

(*E*)-2-(Prop-1-en-1-yl)furan (15a) (81 %, *E*/*Z* = 75:25). The product was prepared following the general procedure for isomerization reactions at a temperature of 40 °C described in section 1.2. It was obtained as inseparable mixture of *E*/*Z* isomers, starting material. NMR data matches values previously reported.[22]

^{1}H NMR (400 MHz, THF-d_8) δ 7.33 (d, *J* = 1.6 Hz, 1H), 6.30 (dd, *J* = 3.3, 1.8 Hz, 1H), 6.22 – 6.17 (m, 1H), 6.17 – 6.05 (m, 2H), 1.81 (dd, *J* = 6.5, 1.3 Hz, 3H).

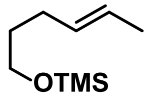

***(E)*-(Hex-4-en-1-yloxy)trimethylsilane (16a) (Isolated yield: 72 %, pale yellow oil, E/Z = 86:14).** The product was prepared following the general

procedure for isomerization reactions described in section 1.2. It was obtained as inseparable mixture of *E*/*Z* isomers, starting material and hydrogenated byproduct. NMR data matches values previously reported.[23]

^{1}H NMR (600 MHz, $CDCl_3$) δ 5.47 – 5.33 (m, 2H), 3.56 (t, *J* = 6.5 Hz, 2H), 2.04 – 1.96 (m, 2H), 1.63 (d, *J* = 4.0 Hz, 3H), 1.61 – 1.54 (m, 2H), 0.09 (s, 9H). $^{13}C\{^1H\}$ NMR (151 MHz, $CDCl_3$) δ 130.9, 125.3, 62.2, 32.6, 28.9, 18.0, -0.4.

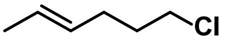

(*E*)-6-Chlorohex-2-ene (17a) (98 %, E/Z = 80:20). The product was prepared following the general procedure for isomerization reactions described in section 1.2.

^{1}H NMR (400 MHz, THF-*d*$_8$) δ 5.54 – 5.31 (m, 2H), 3.51 (t, *J* = 6.7 Hz, 2H), 2.11 (q, *J* = 7.0 Hz, 2H), 1.85 – 1.69 (m, 2H), 1.63 (d, *J* = 6.4 Hz, 3H). HRMS (TOF ESI+): *m/z* calculated for $C_6H_{12}Cl$ $[M+H]^+$: 119.0622, found 119.0619.

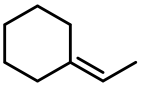

Ethylidenecyclohexane (18a) (90%). The product was prepared following the general procedure for isomerization reactions at a temperature of 40 °C described in section 1.2. NMR data matches values previously reported.[24]

^{1}H NMR (600 MHz, THF-*d*$_8$) δ 5.10 (q, *J* = 6.7 Hz, 1H), 2.16 – 2.11 (m, 2H), 2.07 – 2.02 (m, 2H), 1.53 (d, *J* = 6.6 Hz, 3H), 1.53 – 1.45 (m, 6H).

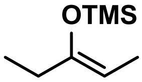

(*Z*)-Trimethyl(pent-2-en-3-yloxy)silane (19a) (72 %, E/Z = 55:45). The product was prepared following the general procedure for isomerization reactions described in section 1.2. It was obtained as inseparable mixture of *E*/*Z* isomers and starting material.[25]

^{1}H NMR (600 MHz, THF-*d*$_8$) δ 4.47 (q, *J* = 6.5 Hz, 1H), 2.06 (q, *J* = 7.5 Hz, 2H), 1.47 (d, *J* = 6.4 Hz, 3H), 1.02 – 0.95 (m, 3H), 0.17 (s, 9H).

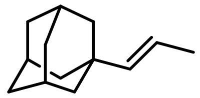

(*E*)-1-(Prop-1-en-1-yl)adamantane (20a) (92 %, *E*/*Z* = 90:10). The product was prepared following the general procedure for isomerization reactions described in section 1.2. NMR data matches values previously reported.[26]

^{1}H NMR (400 MHz, THF-*d*$_8$) δ 5.29 (s, 1H), 5.30 – 5.20 (m, 1H), 1.94 (br, 3H), 1.74 – 1.63 (m, 6H), 1.61 (d, *J* = 4.9 Hz, 3H), 1.57 (br d, *J* = 2.6 Hz, 6H).

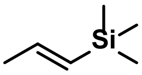

(*E*)-Trimethyl(prop-1-en-1-yl)silane (21a) (80 %, E/Z = 94:6). The product was prepared following the general procedure for isomerization reactions described in section 1.2. NMR data matches values previously reported.[27]

^{1}H NMR (400 MHz, THF-*d*$_8$) δ 6.13 – 5.98 (m, 1H), 5.69 – 5.61 (m, 1H), 1.78 (dd, *J* = 6.2, 1.4 Hz, 3H), 0.03 (s, 9H).

2.2 Products of Chain-Walking Isomerization

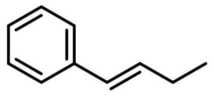

(*E*)-But-1-en-1-yl-benzene (12b) (89 %, *E*/*Z* = 90:10). The product was prepared following the general procedure for isomerization reactions described in section 1.2. To obtain a suitable ^{1}H NMR spectrum the reaction mixture was filtered through a syringe filter under argon atmosphere prior to recording. It was obtained as a mixture of starting material and monoisomerized product **(*E*)-But-2-en-1-ylbenzene (12a)**. NMR data matches values previously reported. NMR data matches values previously reported.[28]

^{1}H NMR (400 MHz, THF-d_8) δ 7.36 – 7.26 (m, 2H), 7.23 (t, J = 7.6 Hz, 2H), 7.12 (t, J = 7.3 Hz, 1H), 6.38 (d, J = 15.9 Hz, 1H), 6.27 (dt, J = 15.8, 6.3 Hz, 1H), 2.27 – 2.15 (m, 2H), 1.08 (t, J = 7.5 Hz, 3H).

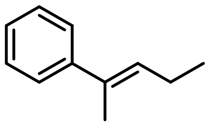

(*E*)-Pent-2-en-2-ylbenzene (13b) (44 %, *E*/*Z* = 99:1). The product was prepared following the general procedure for isomerization reactions described in section 1.2. To obtain a suitable ^{1}H NMR spectrum the reaction mixture was filtered through a syringe filter under argon atmosphere prior to recording. It was obtained as a mixture of starting material and monoisomerized product **(*E*)-Pent-3-en-2-ylbenzene (13a)**. NMR data matches values previously reported.[7]

^{1}H NMR (400 MHz, THF-d_8) δ 7.38 – 7.32 (m, 2H), 7.25 – 7.20 (m, 3H), 5.78 (tq, J = 7.2, 1.6 Hz, 1H), 2.21 (p, J = 7.3 Hz, 2H), 2.01 (s, 3H), 1.06 (t, J = 7.5 Hz, 3H).

3 Mechanistic Studies

3.1 Isomerization Experiments with Hydride Complexes

Inside an argon flushed glovebox a NMR tube was charged with solution of **[Mn]** (0.013 mmol, 2.5 mol%) in THF-d_8 (0.500 mL) and dioxane (0.011 mL, 0.13 mmol) as internal standard. Allylbenzene (0.50 mmol) was added and the NMR tube was left to stand at room temperature for 22 h. The progress of the reaction was monitored by ^{1}H NMR. After 22 h the reaction was quenched by exposure to air and GC-MS analysis was performed using butylbenzene as internal standard.

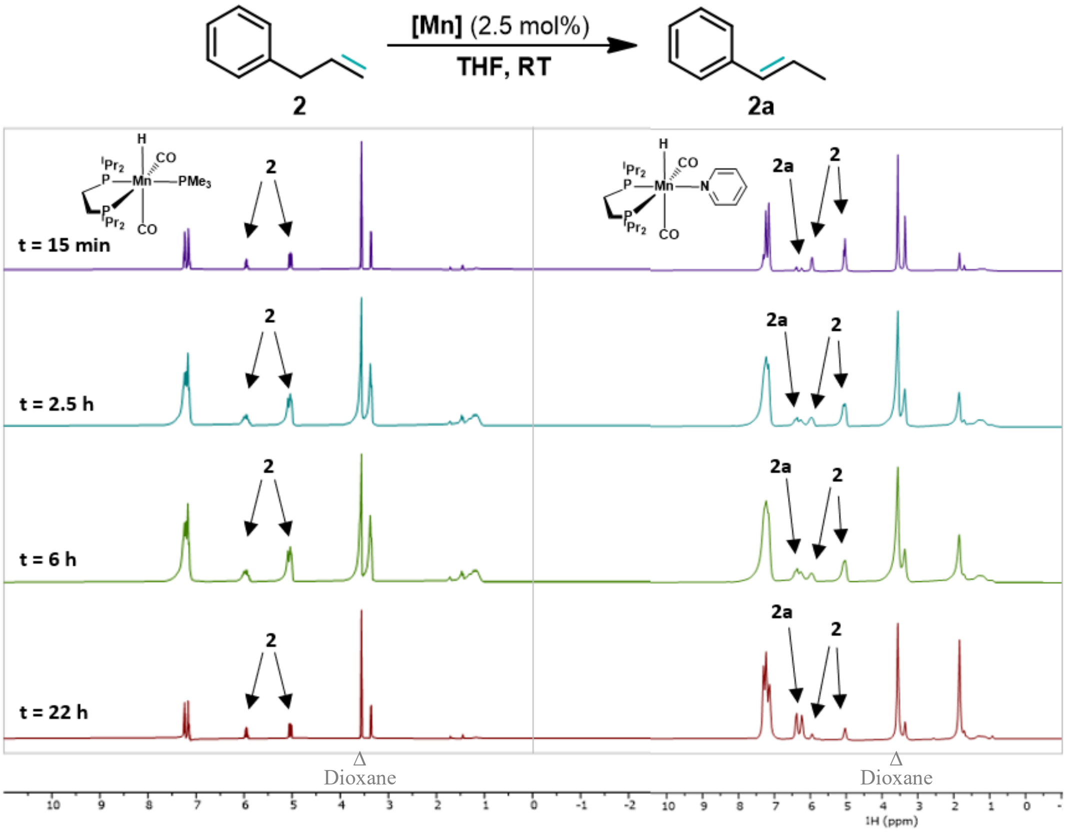

Scheme S 1. ^{1}H NMR monitoring of the isomerization reaction catalyzed by **Mn6** (left) and **Mn5** (right). Characteristic double-bond signals of the starting material allylbenzene (**2**) and the product (E)-prop-1-en-1-ylbenzene (**2a**) are highlighted.

3.2 Isomerization Monitoring

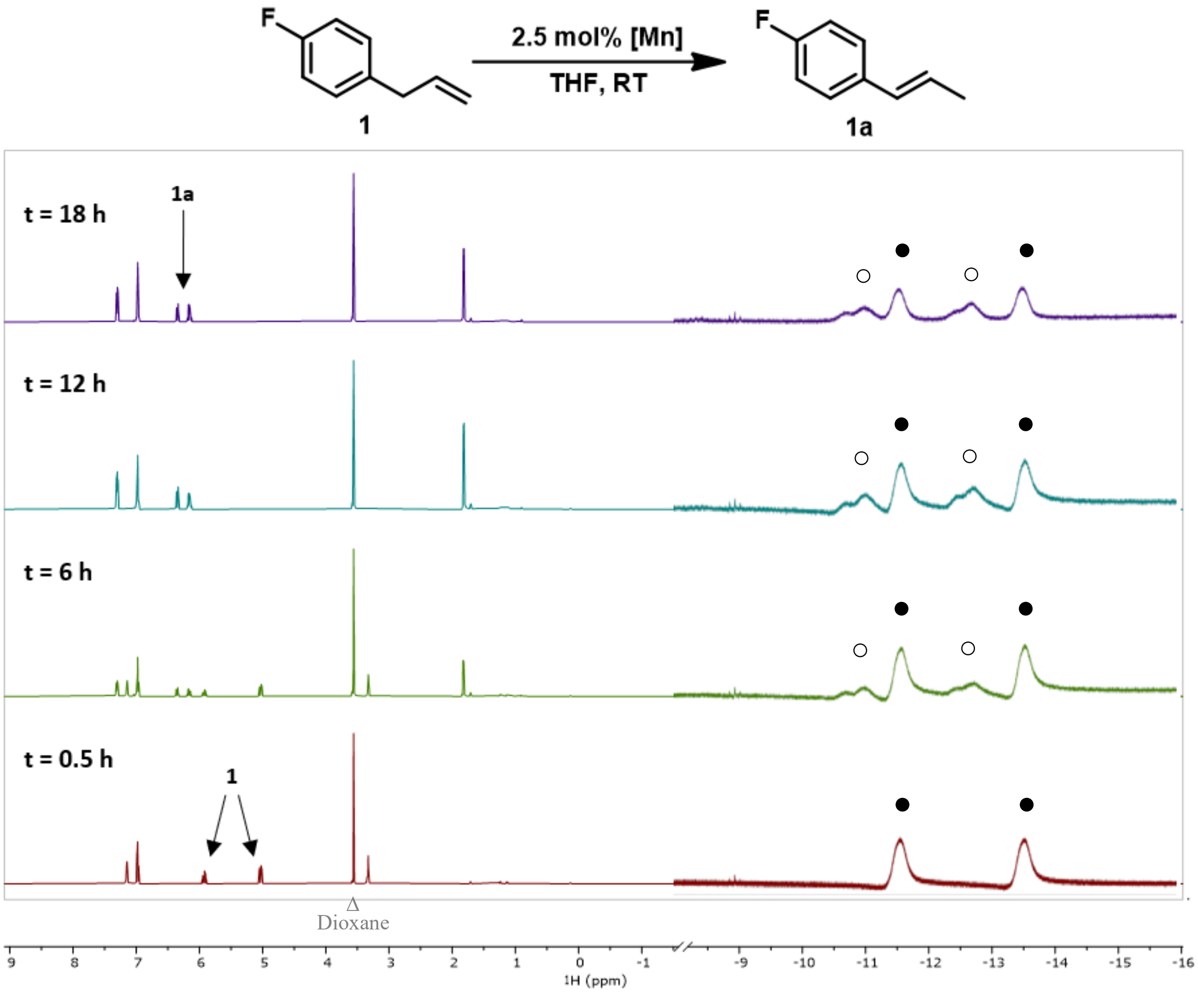

Scheme S 2. ^{1}H NMR spectra of the isomerization of 1-allyl-4-fluorobenzene (**1**) recorded after 0.5, 6, 12 and 18 h (bottom to top) at room temperature. The magnified hydride region shows the formation of a potential active species (○) alongside the hydride signals of the unchanged complex **Mn4** (●).

3.3 Alternative Activation Experiments with Mn1

Attempted activation of Mn1 with $NaBH_4$/MeOH

Inside an argon flushed glovebox an 8 mL screw cap vial was charged with a stirring bar, 4-fluoro allylbenzene (0.5 mmol), **Mn1** (2.5 mol%), $NaBH_4$ (5.0 mol%), *n*-butylbenzene (1.0 mmol, as internal standard for GC-MS). Methanol (0.5 mL) were added, the vial was quickly sealed and stirred at room temperature for 18h. The reaction was quenched by exposure to air and the sample analyzed by GC-MS). No double bond isomerization could be observed.

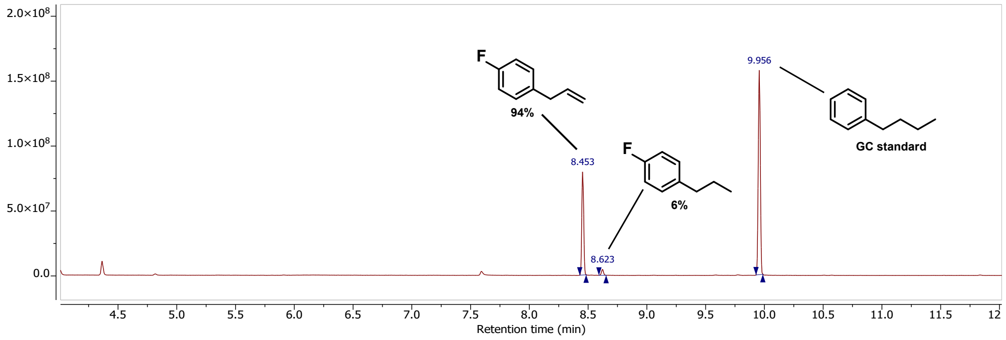

Figure S 1. GC-MS spectrum of the activation experiment conducted with **Mn1** and $NaBH_4$ in MeOH after 18 h.

Attempted activation of Mn1 with $NaBH_4$/MeOH

Inside an argon flushed glovebox a J. Young tube was charged with 4-fluoro allylbenzene (0.5 mmol), **Mn1** (2.5 mol%) and 1,4-dioxane (0.013 mmol, as internal standard). THF-*d*$_8$ (0.5 mL) was added, the tube was sealed and transferred out of the glovebox. The atmosphere was exchanged to 1 bar of hydrogen gas by three freeze-pump-thaw cycles on the Schlenk line. ^{1}H NMRs was periodically recorded. No double bond isomerization could be observed over a period of 18 hours.

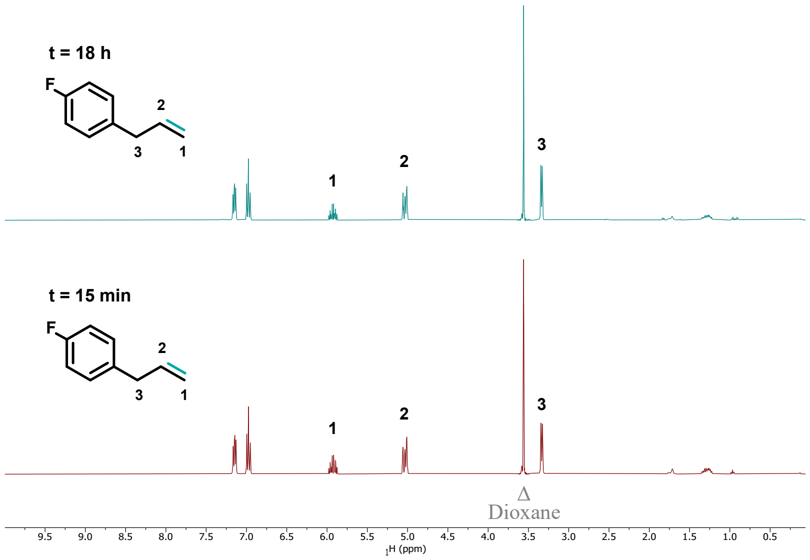

Figure S 2. Stacked ^{1}H NMR spectra of the activation experiment conducted with **Mn1** under 1 bar H_2 pressure.

3.4 Deuterium Labeling Experiment

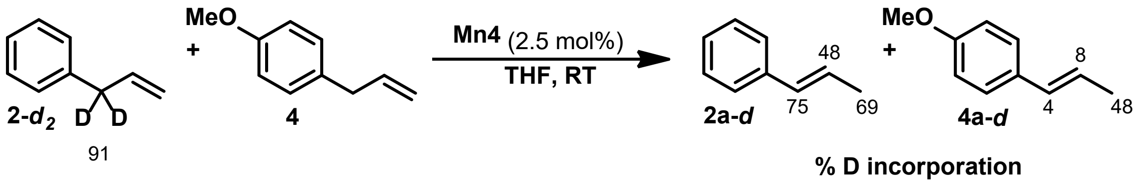

Inside an argon flushed glovebox an NMR tube was charged with solution of **Mn4** (0.0049 g, 0.013 mmol, 2.5 mol%) in THF-d_8 (0.500 mL) and dioxane (0.011 mL, 0.13 mmol) as internal standard. Allylbenzene-d_2 (0.25 mmol) and non-deuterated 4-allylanisole (0.25 mmol) were added. NMR measurements were performed after 18 h reaction time at room temperature.

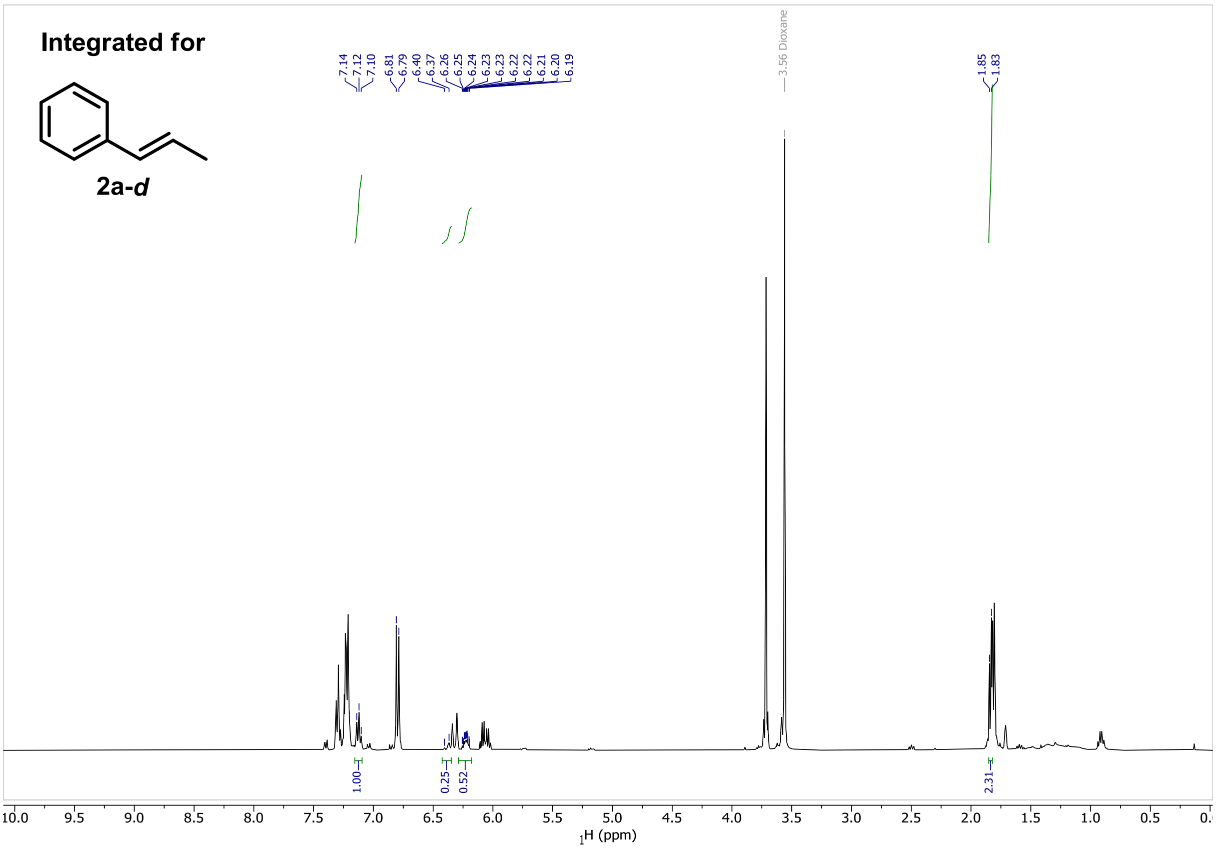

Figure S 3. ¹H NMR spectrum of the deuterium labeling experiment after 18 h with characteristic peaks assigned to (*E*)-prop-1-en-1-ylbenzene-d (**2a-d**).

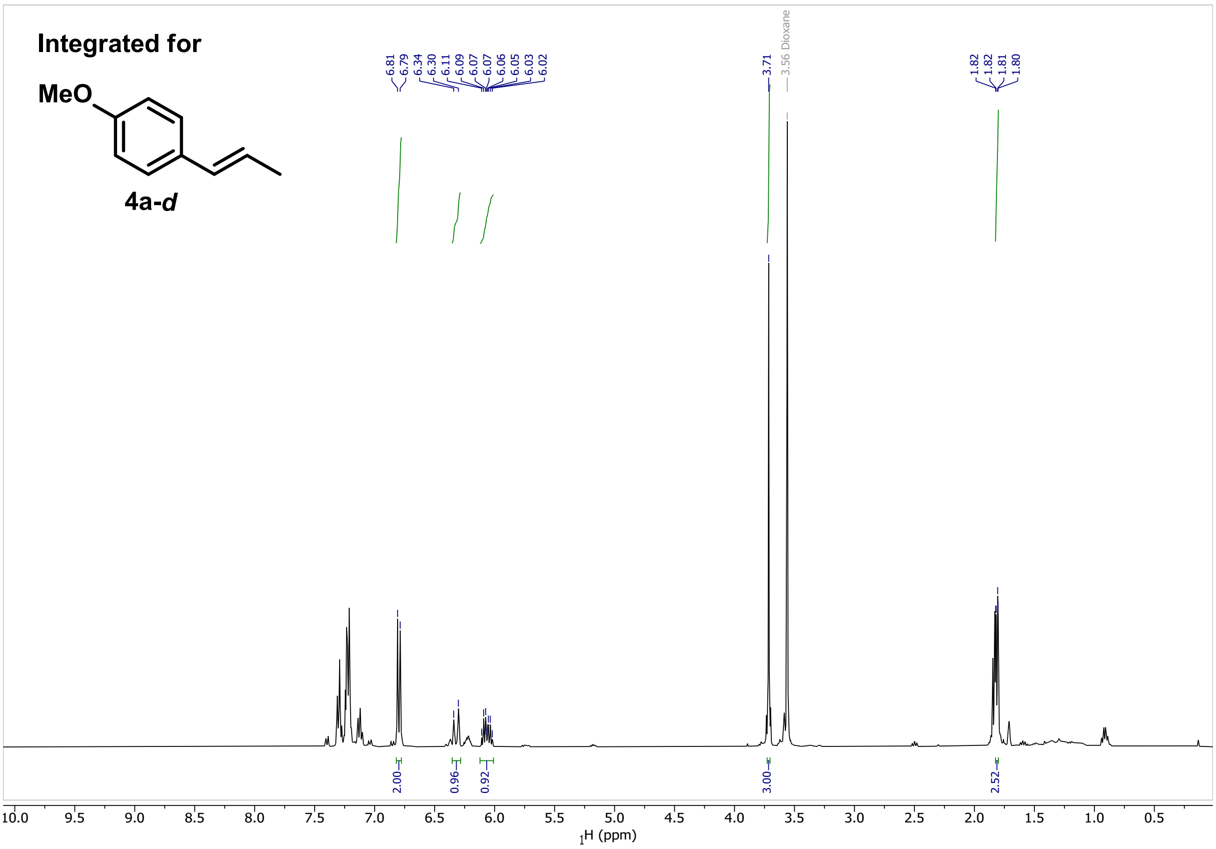

Figure S 4. ^{1}H NMR spectrum of the deuterium labeling experiment after 18 h with characteristic peaks assigned to (*E*)-anethole-d (**4a-d**).

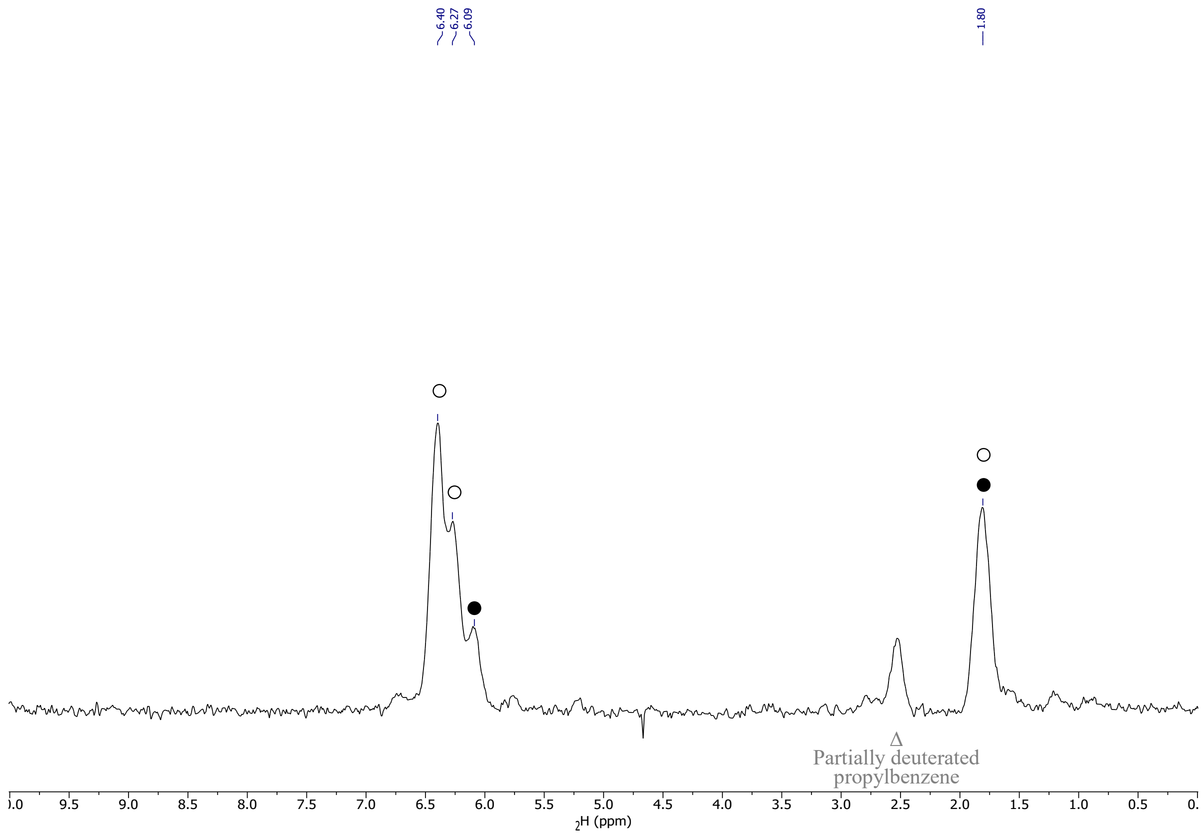

Figure S 5. ^{2}H NMR spectrum of the deuterium labeling experiment after 18 h with peaks of **2a-d** (○) and **4a-d** (●) assigned. The signal at 2.53 ppm is assigned to the deuterated benzylic position of propylbenzene obtained from the reduction of **2a-d$_2$**.

3.5 Spectra of Consecutive Chain-Walking Experiments

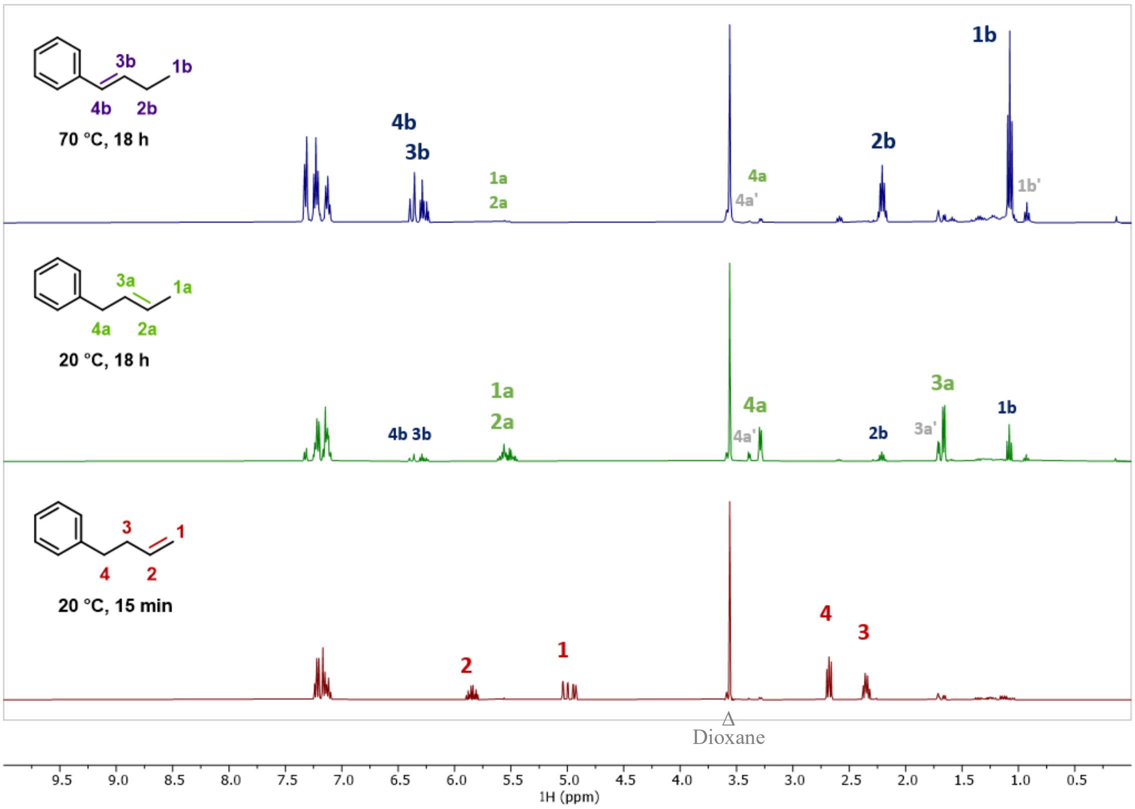

Figure S 6. Stacked ^{1}H NMR spectra of the isomerization of **12** (bottom) to the 2-alkene **12a** (middle) and subsequently to the thermodynamic product **12b**. Characteristic signals of the respective Z-isomers are marked with inverted commas.

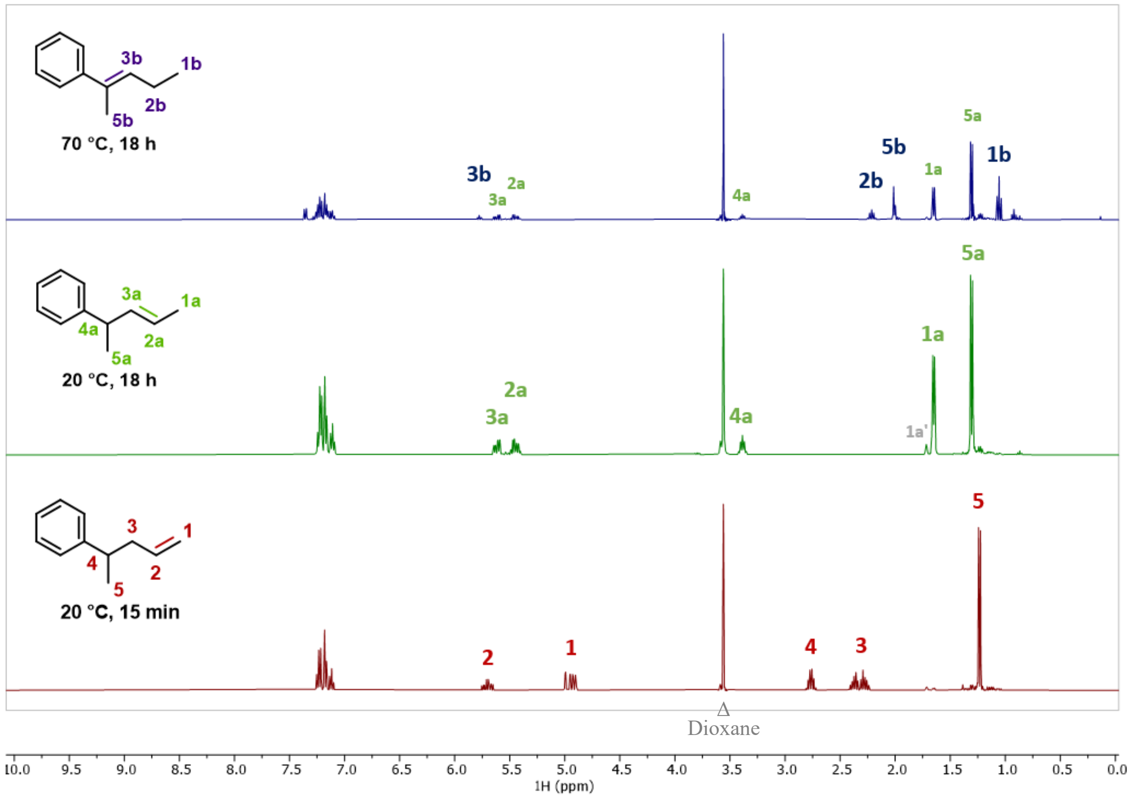

Figure S 7. Stacked ^{1}H NMR spectra of the isomerization of **13** (bottom) to the 2-alkene **13a** (middle) and subsequently to the thermodynamic product **13b**. Characteristic signals of the respective Z-isomers are marked with inverted commas.

4 Computational Details

The computational results presented have been achieved in part using the Vienna Scientific Cluster (VSC). All calculations were performed using the GAUSSIAN 09 software package[29] without symmetry constraints. The optimized geometries were obtained with the the PBE0 functional. That functional uses a hybrid generalized gradient approximation (GGA), including 25 % mixture of Hartree-Fock[30] exchange with DFT[31] exchange-correlation, given by Perdew, Burke and Ernzerhof functional (PBE).[32] The basis set used for the geometry optimizations (basis b1) consisted of the Stuttgart/Dresden ECP (SDD) basis set[33] to describe the electrons of Mn, and a standard 6-31G(d,p) basis set[34] for all other atoms. Transition state optimizations were performed with the Synchronous Transit-Guided Quasi-Newton Method (STQN) developed by Schlegel *et al*,[35] following extensive searches of the Potential Energy Surface. Frequency calculations were performed to confirm the nature of the stationary points, yielding one imaginary frequency for the transition states and none for the minima. Each transition state was further confirmed by following its vibrational mode downhill on both sides and obtaining the minima presented on the energy profiles. The electronic energies (E_{b1}) obtained at the PBE0/b1 level of theory were converted to free energy at 298.15 K and 1 atm (G_{b1}) by using zero point energy and thermal energy corrections based on structural and vibration frequency data calculated at the same level.

Single point energy calculations were performed on the geometries optimized at the PBE0/b1 level, using the M06 functional and a standard 6-311++G(d,p) basis set.[36] The M06 functional is a hybrid meta-GGA functional developed by Truhlar and Zhao,[37] and it was shown

to perform very well for the kinetics of transition metal molecules, providing a good description of weak and long range interactions.[38] Solvent effects (THF) were considered in *all* calculations (PBE0/b1 geometry optimizations included) using the Polarizable Continuum Model (PCM) initially devised by Tomasi and coworkers[39] with radii and non-electrostatic terms of the SMD solvation model, developed by Truhlar *et al.*[40] The free energy values presented (G_{b2}) were derived from the electronic energy values obtained at the M06/6-311++G(d,p)//PBE0/b1 level (E_{b2}) according to the following expression: $G_{b2} = E_{b2} + G_{b1} - E_{b1}$.

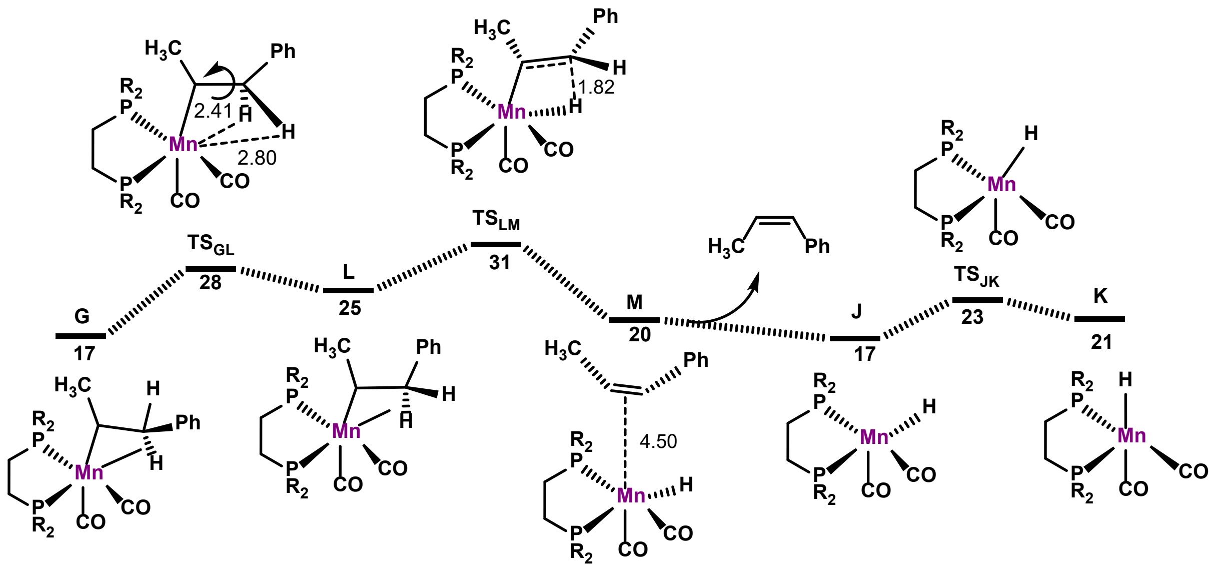

Figure S 8. Free Energy Profile Calculated for the Isomerization of Allylbenzene to Form *Z*-Prop-1-en-1-ylbenzene. Free Energies (kcal/mol) are Referred to fac-[Mn(dippe)(CO)$_2$(κ^2-BH$_4$)] (**Mn4**) (**A** in the calculation in the form of **Mn4**·CH$_2$=CHCH$_2$Ph).

5 Spectra

5.1 Spectra of Complexes

Spectra of Mn4

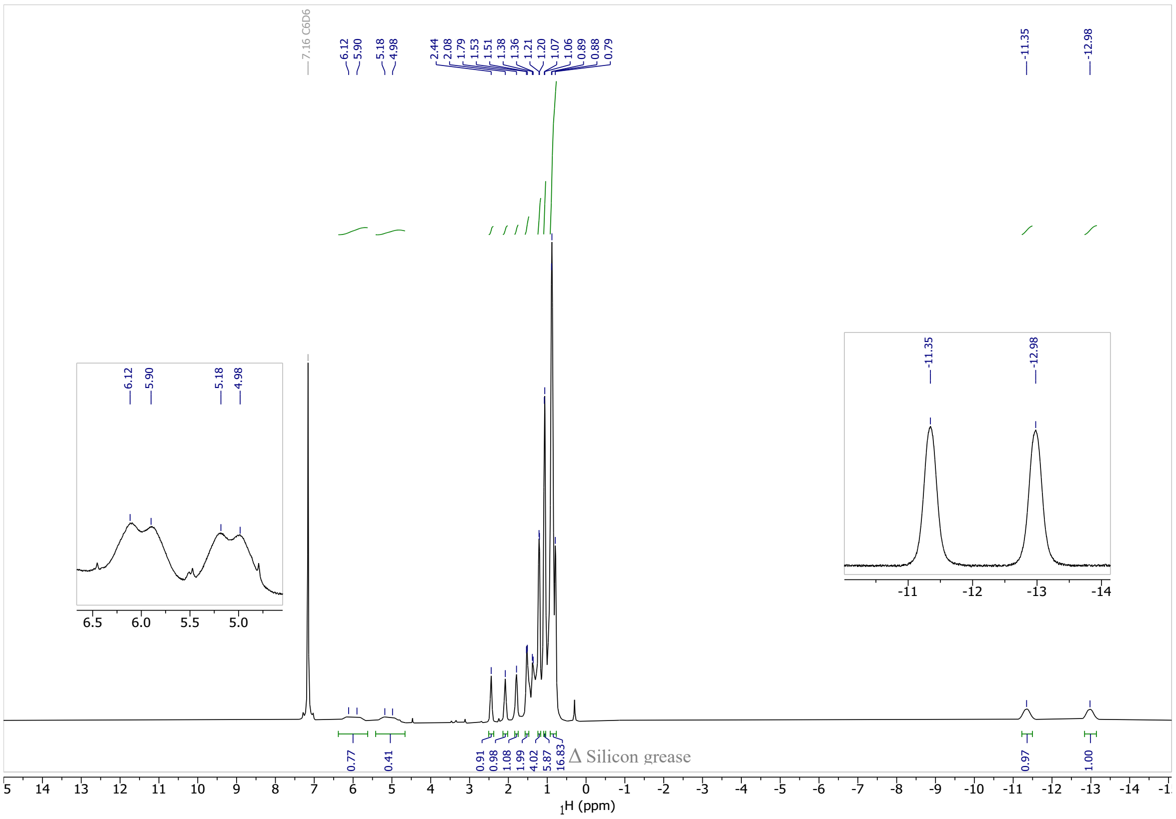

Figure S 9. ^{1}H NMR spectrum of **Mn4**. Inset: Detailed view of the boron-bound hydrogen atoms.

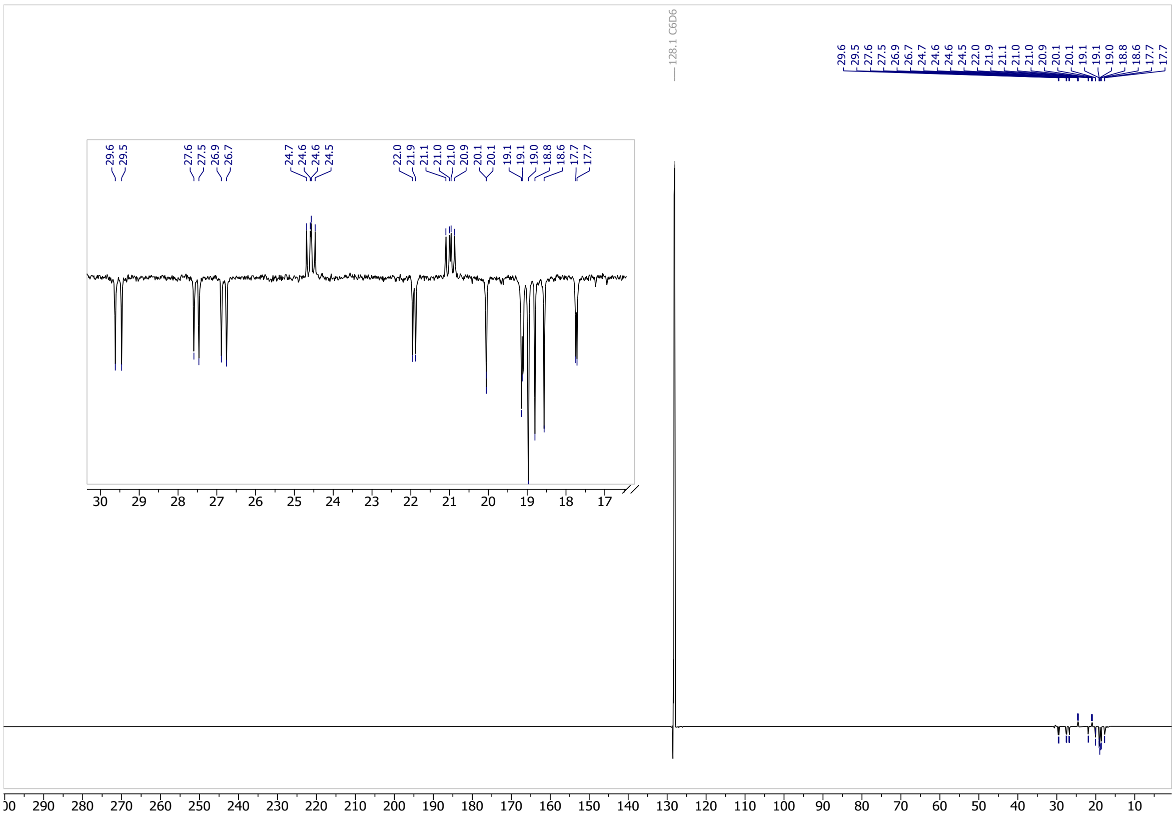

Figure S 10. $^{13}C\{^1H\}$ APT NMR spectrum of **Mn4**.

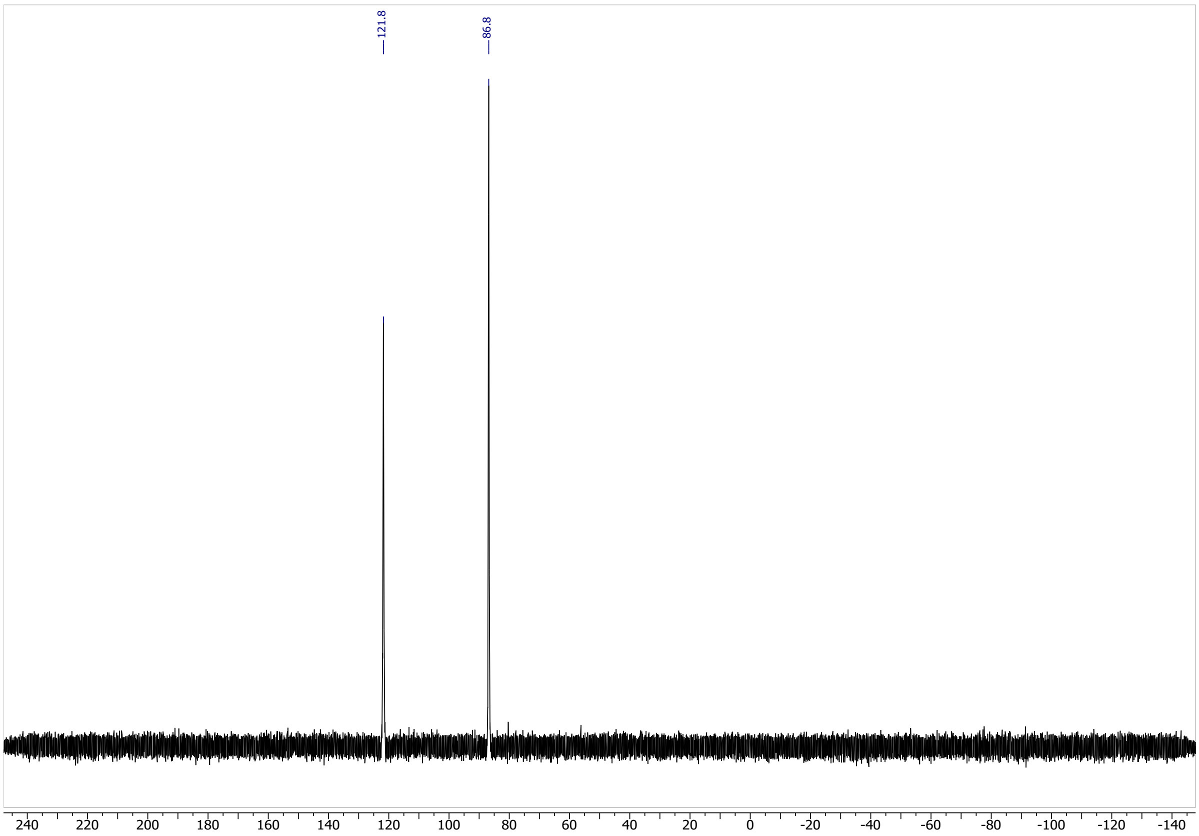

Figure S 11. $^{31}P\{^1H\}$ NMR spectrum of **Mn4**.

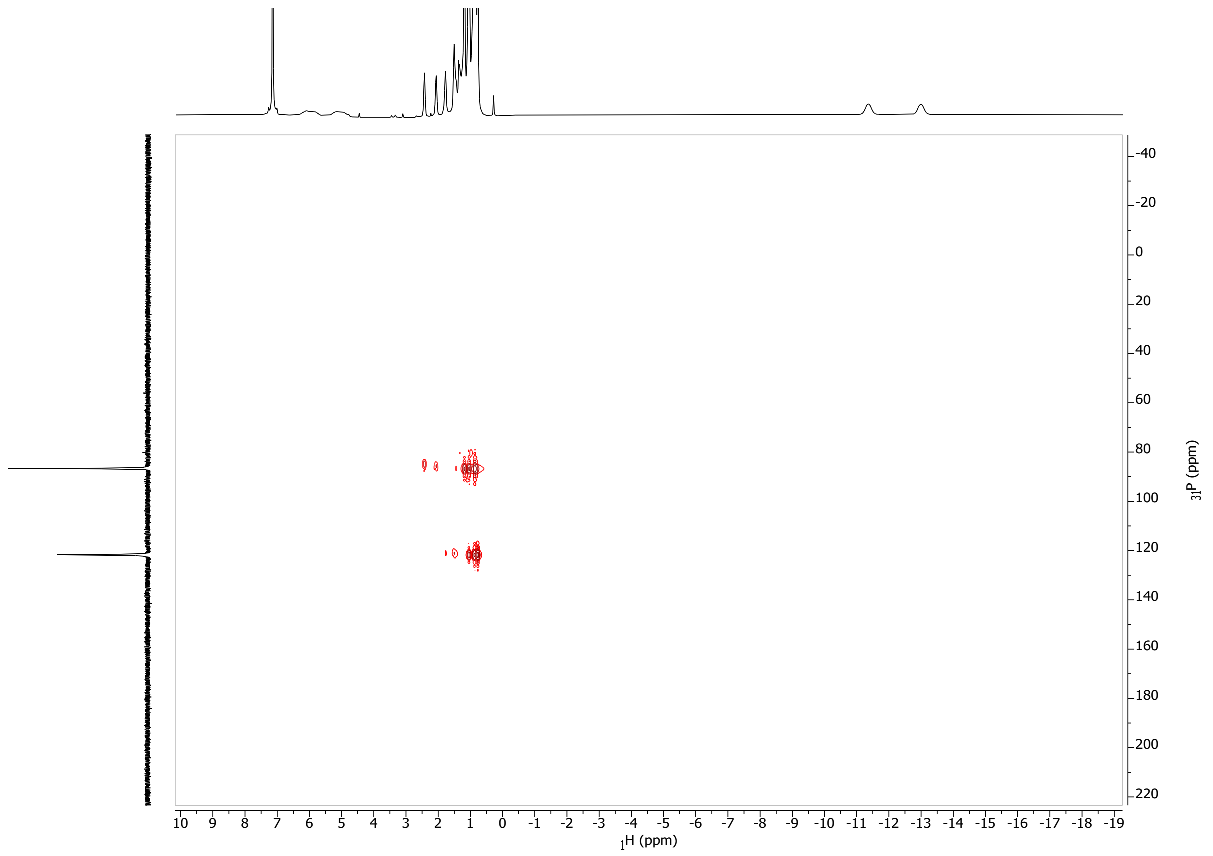

Figure S 12. $^{1}H/^{31}P$ HMBC NMR spectrum of **Mn4**.

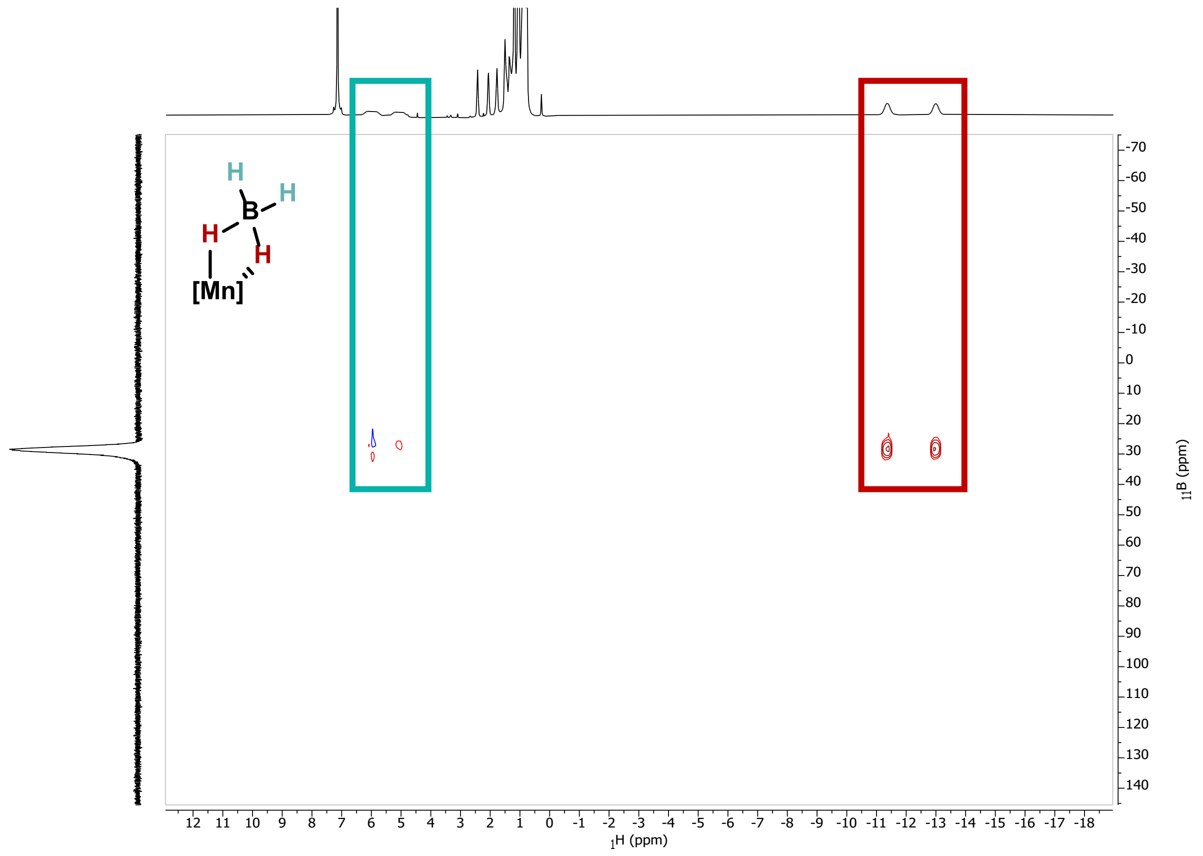

Figure S 13. $^{1}H/^{11}B$ HSQC NMR spectrum of **Mn4**.

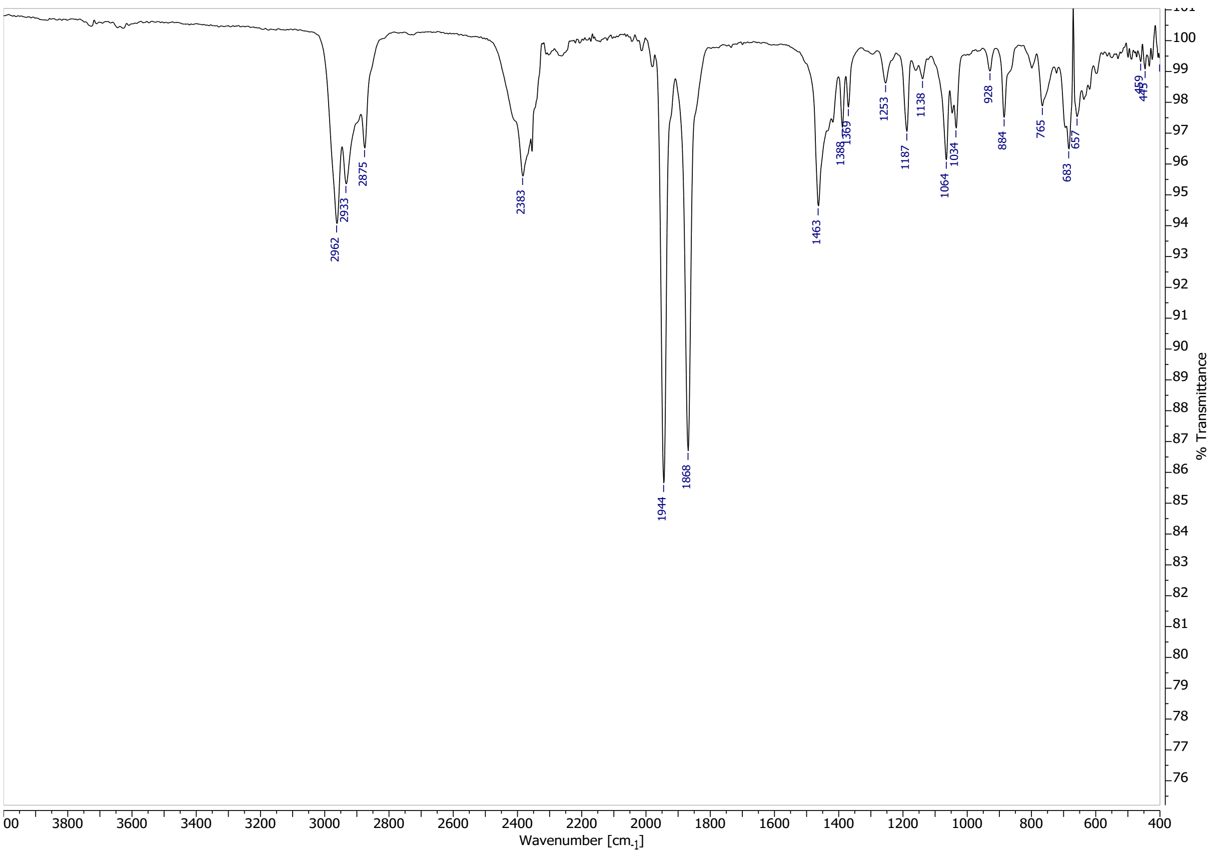

Figure S 14. ATR-IR spectrum of **Mn4**.

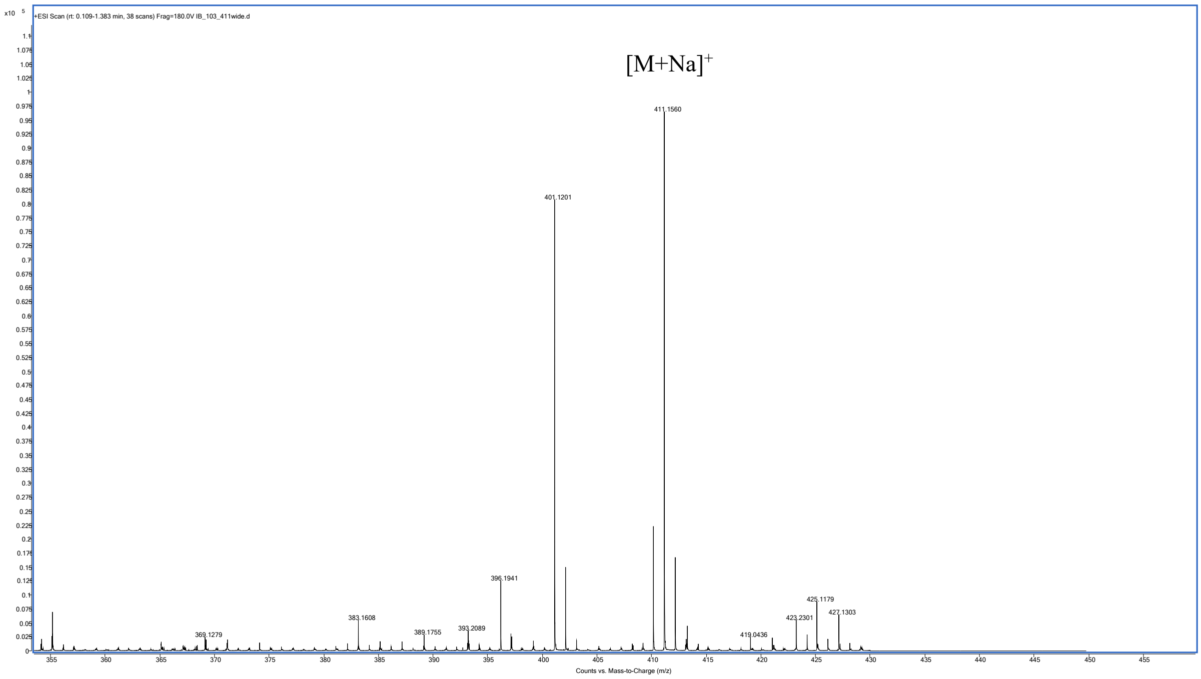

Figure S 15. High Resolution Mass Spectrum of **Mn4**.

Spectra of Mn5

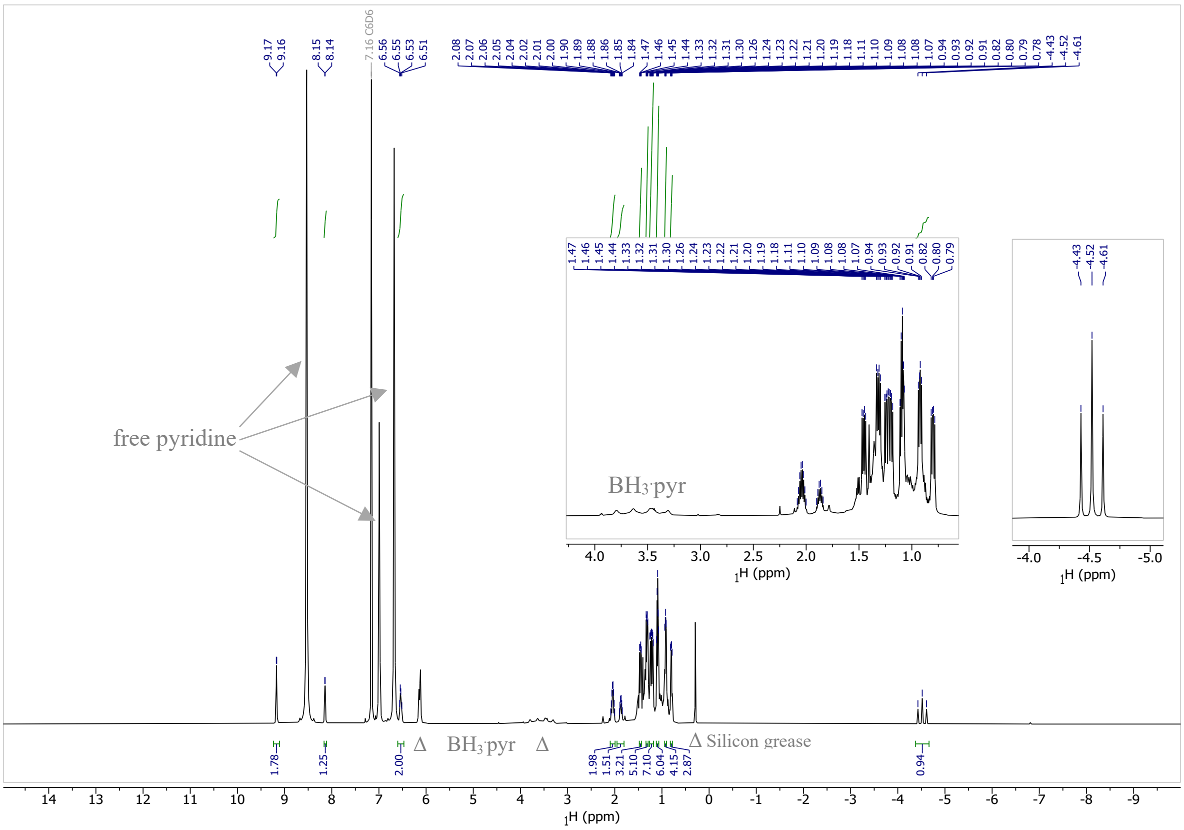

Figure S 16.^{1}H NMR spectrum of **Mn5** (other BH_3·pyr signals indetectable due to signal overlap). Inlet: Detailed view of the aliphatic and hydride region.

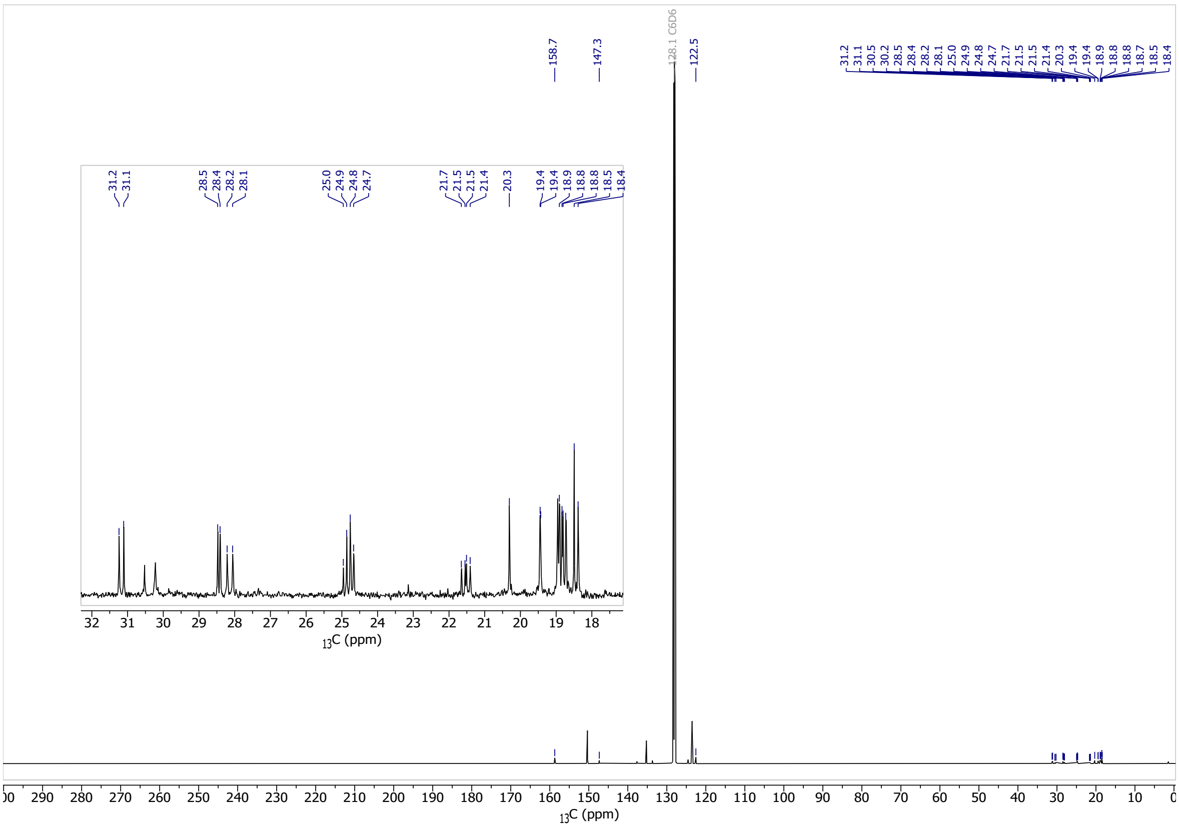

Figure S 17. $^{13}C\{^{1}H\}$ NMR spectrum of **Mn5**. Inlet: Detailed view of the aliphatic region.

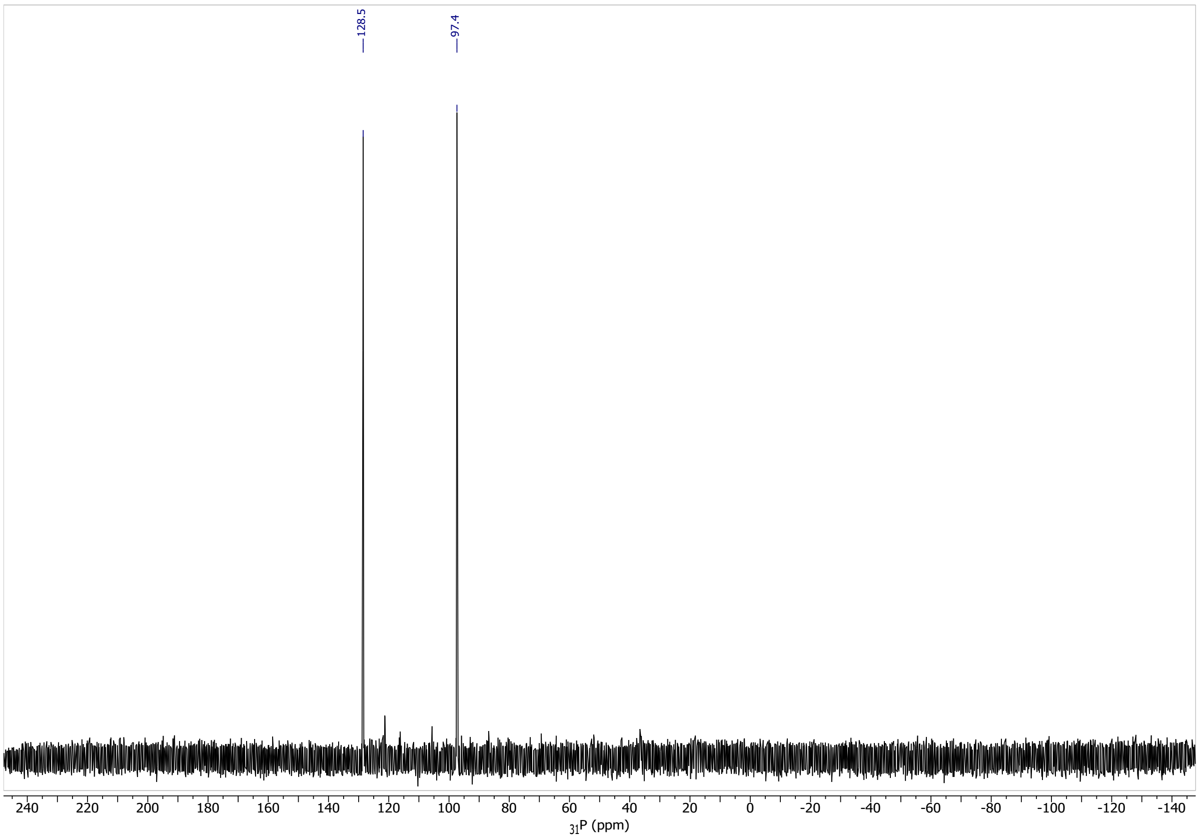

Figure S 18. $^{31}P\{^{1}H\}$ NMR spectrum of **Mn5**.

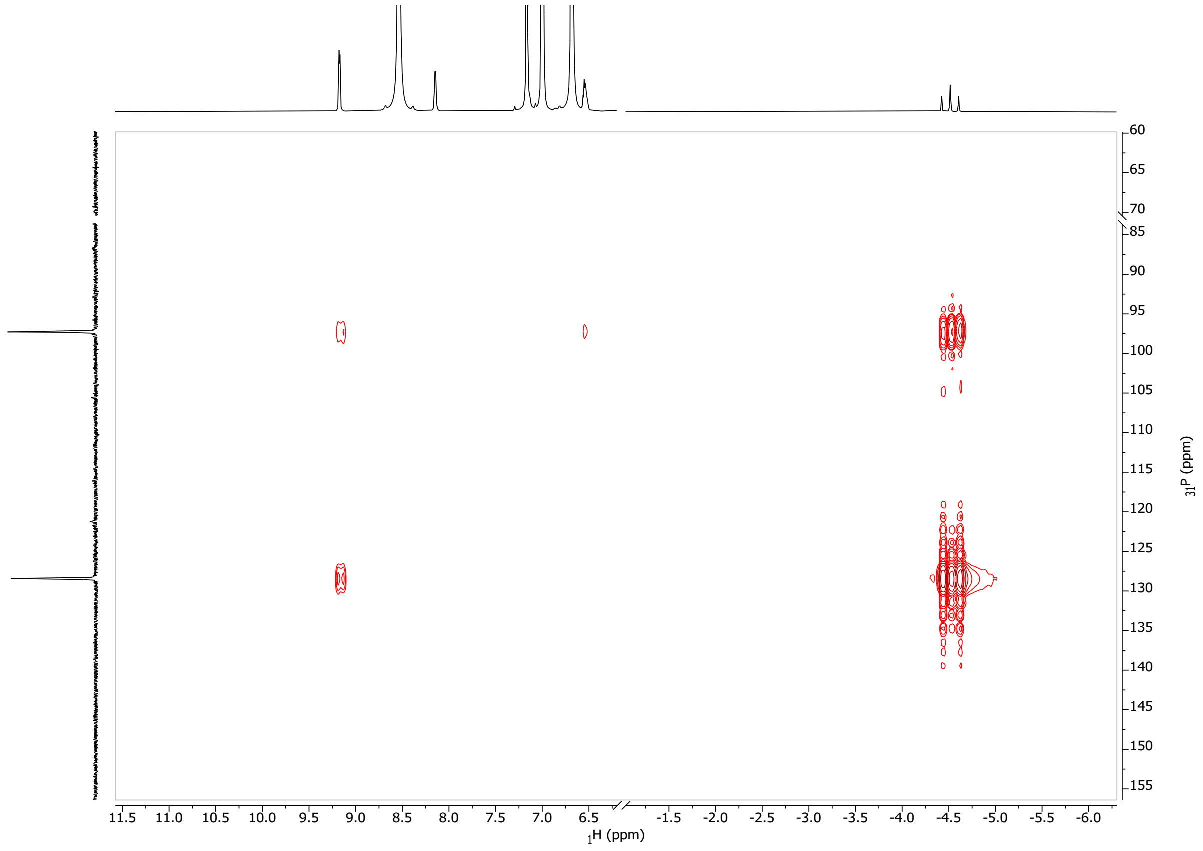

Figure S 19. $^{1}H/^{31}P$ HMBC NMR spectrum of **Mn5** (aromatic and hydride region).

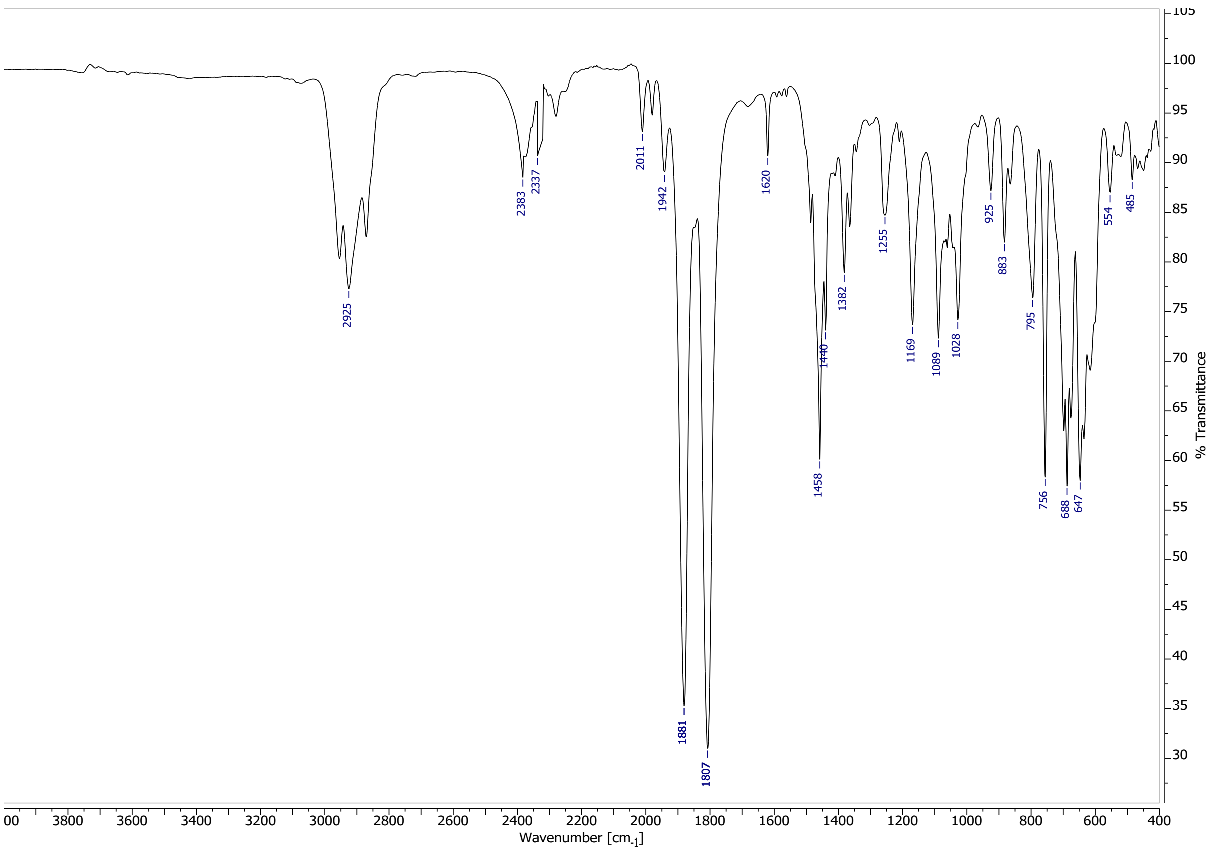

Figure S 20. ATR-IR spectrum of **Mn5**.

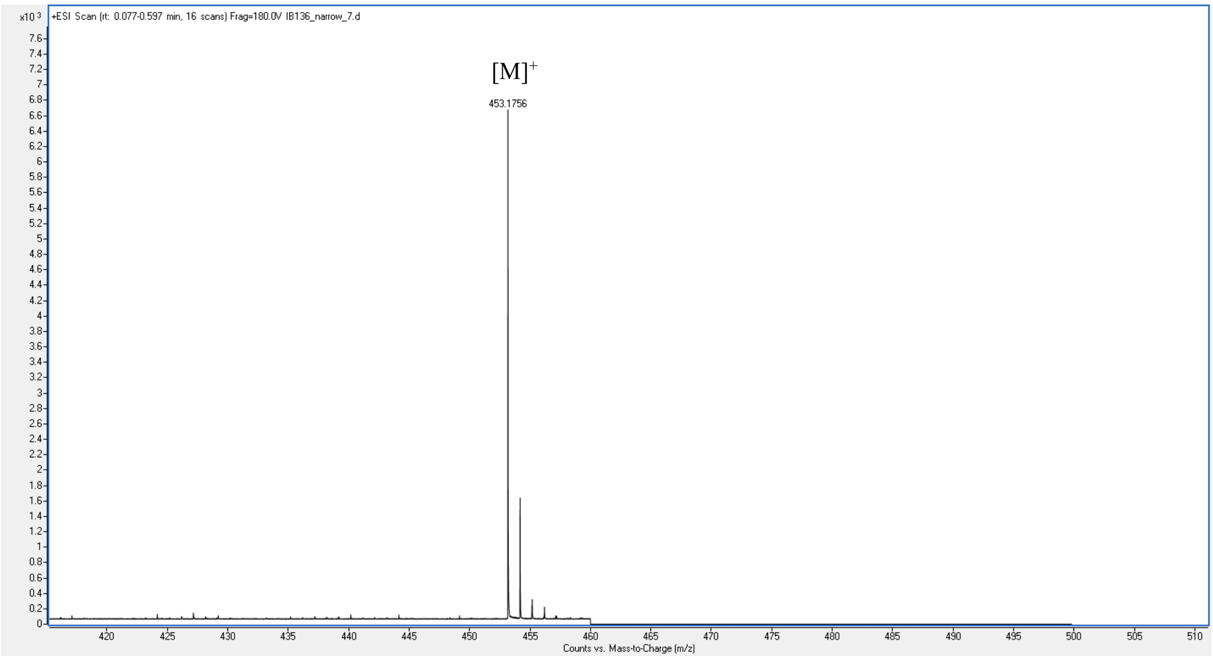

Figure S 21. High Resolution Mass Spectrum of **Mn5**.

Spectra of Mn6

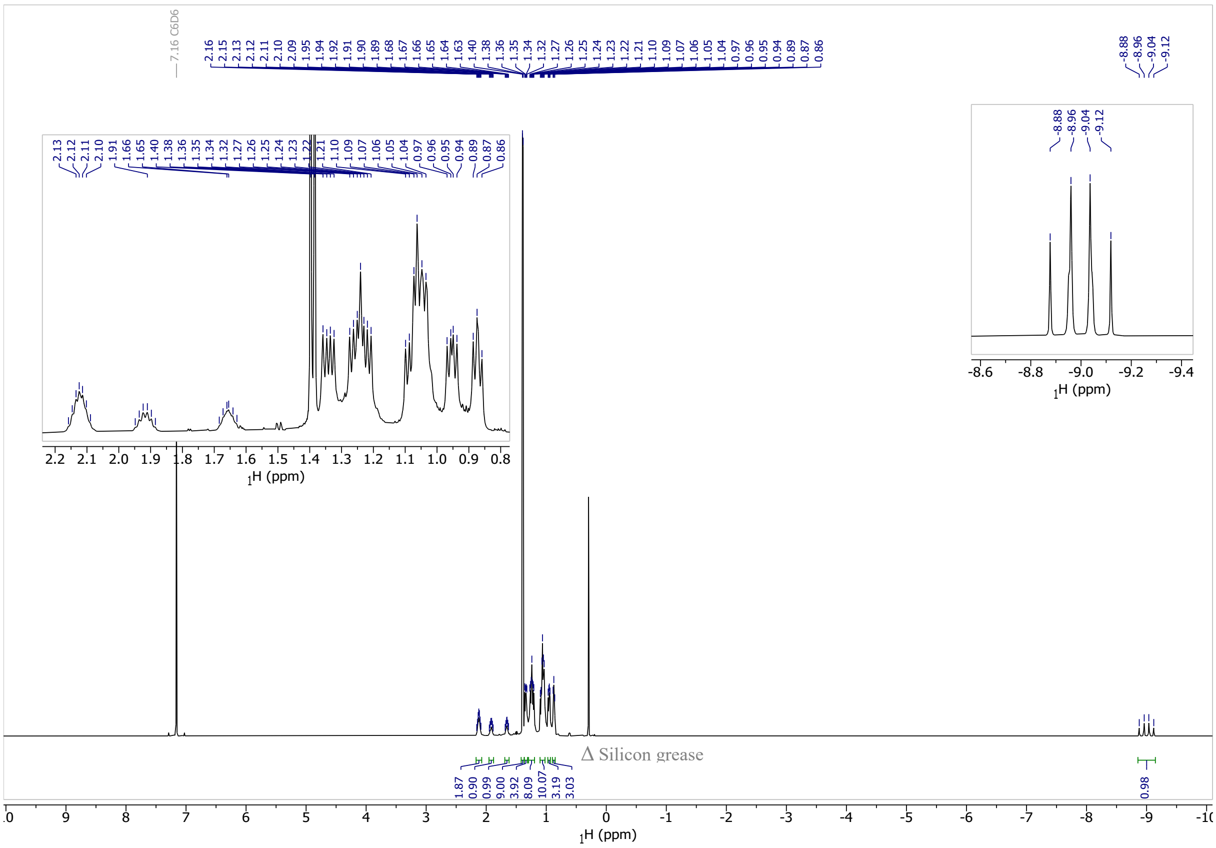

Figure S 22. ^{1}H NMR spectrum of **Mn6**.

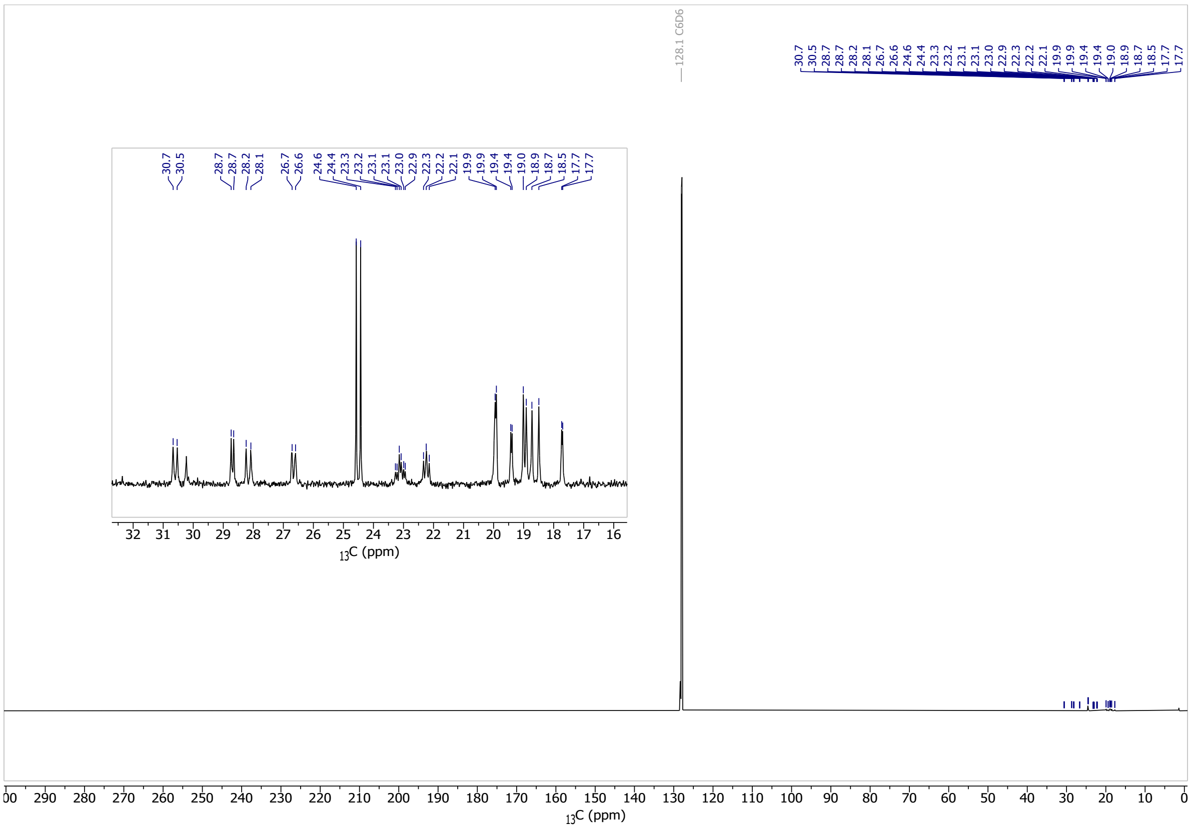

Figure S 23. $^{13}C\{^{1}H\}$ NMR spectrum of **Mn6**.

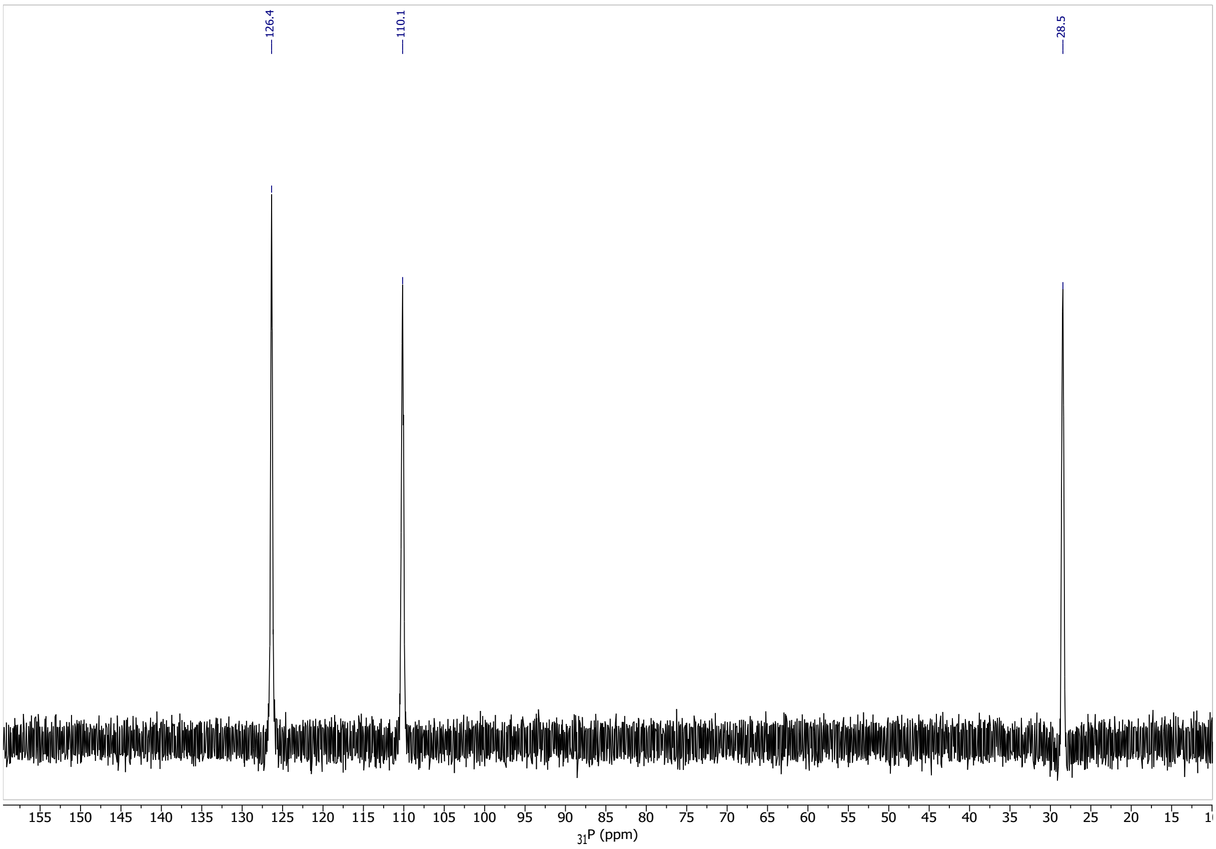

Figure S 24. $^{31}P\{^{1}H\}$ NMR spectrum of **Mn6**.

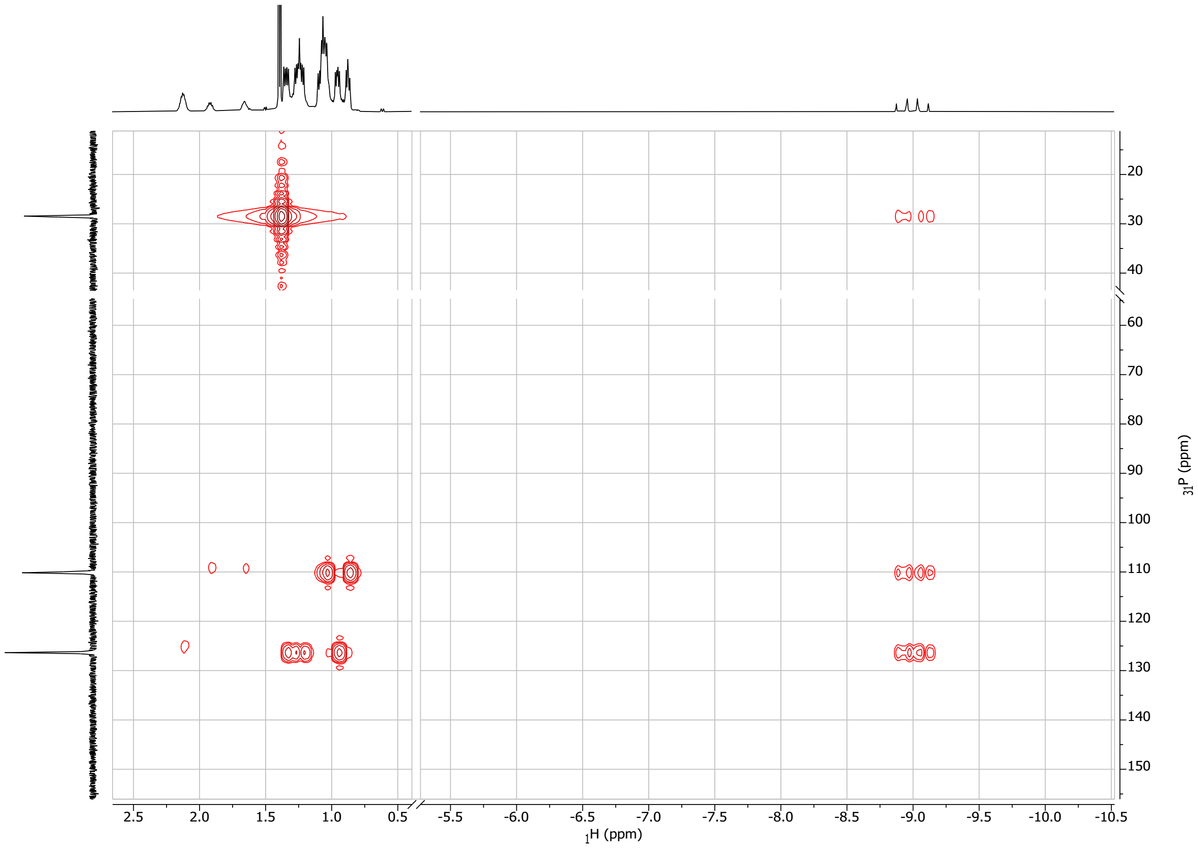

Figure S 25. $^{1}H/^{31}P$ HMBC NMR spectrum of **Mn6**.

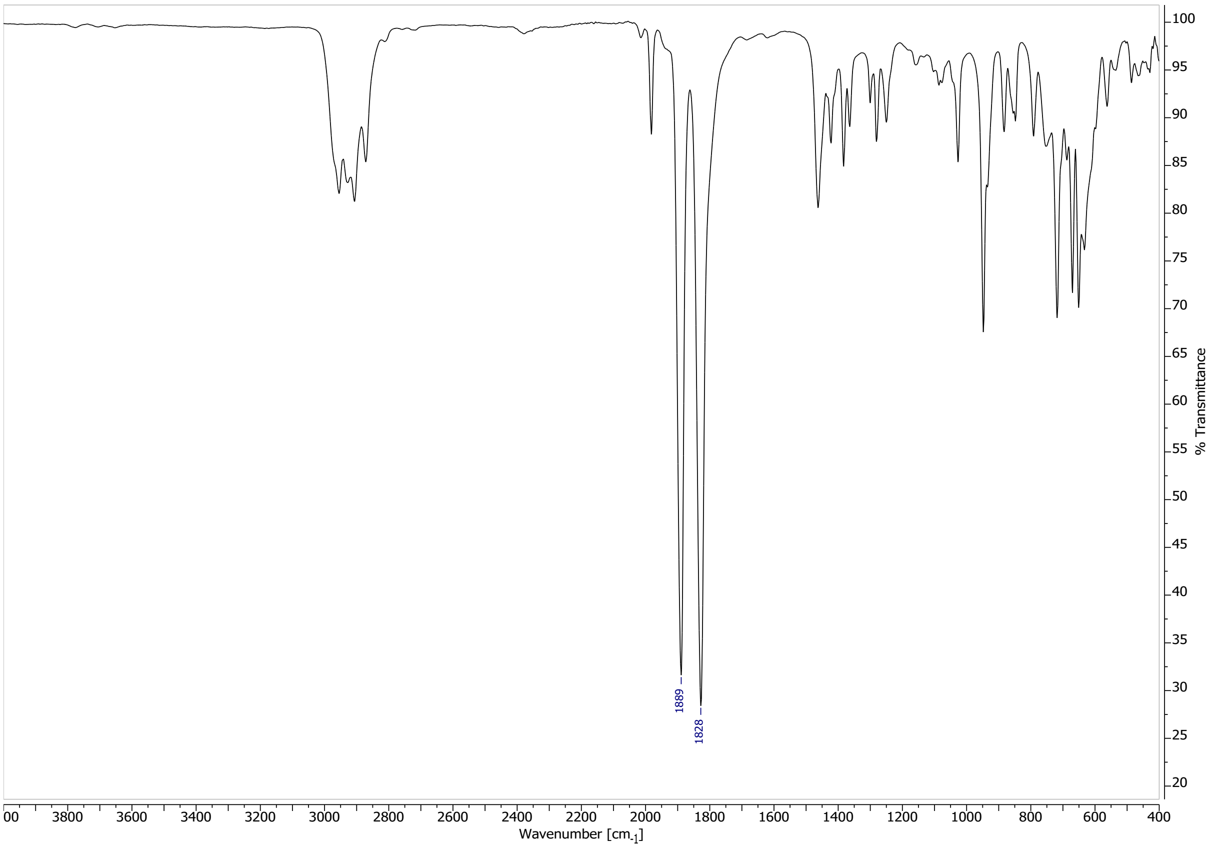

Figure S 26. ATR-IR spectrum of **Mn6**.

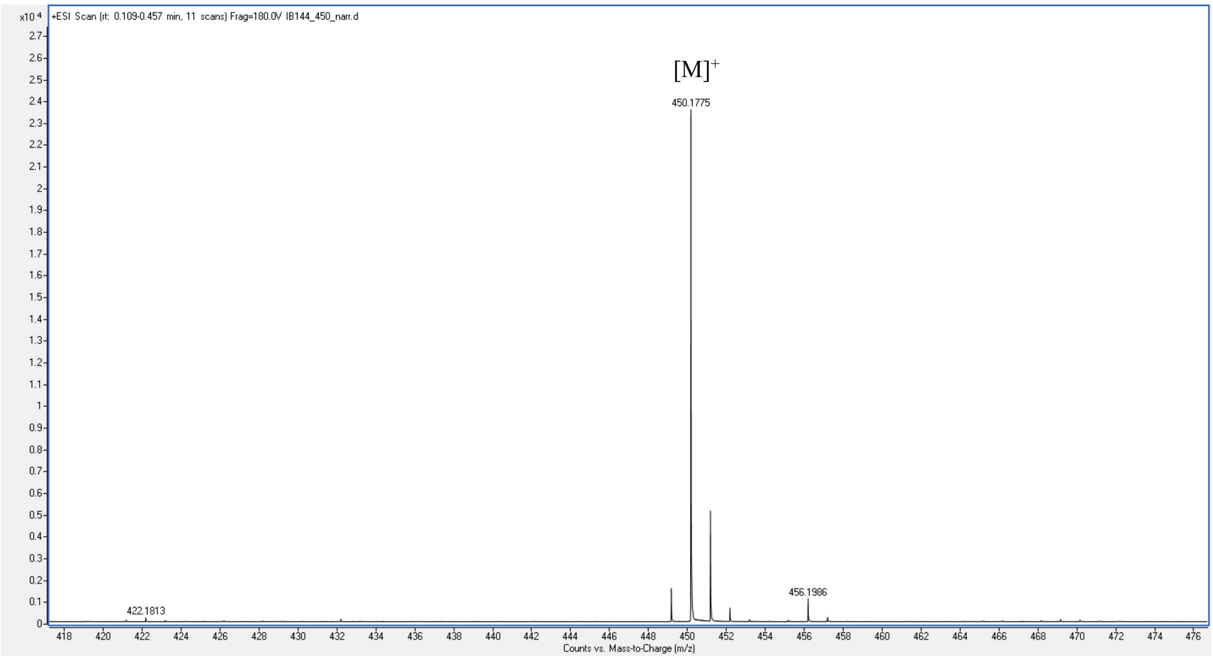

Figure S 27. High Resolution Mass Spectrum of **Mn6**.

5.2 NMR Spectra of Synthesized Substrates

1-Allyl-2-methylbenzene (8)

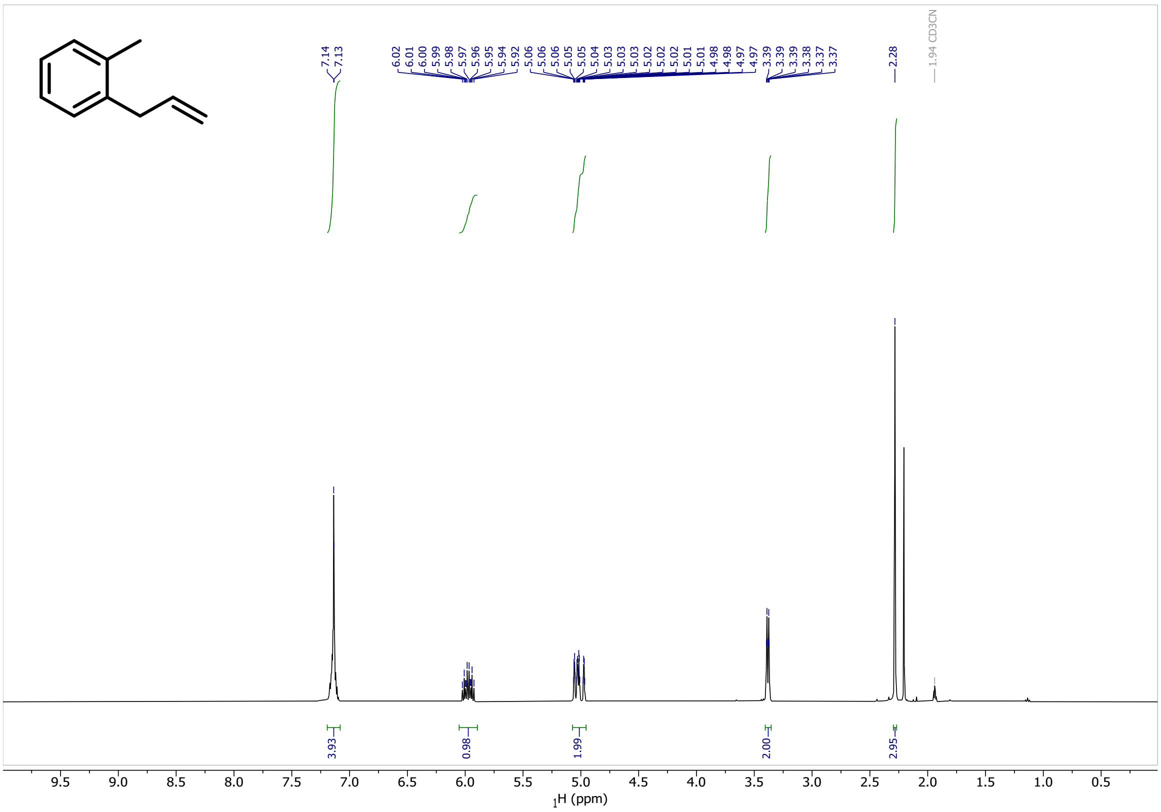

Figure S 28. ^{1}H NMR spectrum of synthesized substrate **8**.

4-Allyl-1,1'-biphenyl (9)

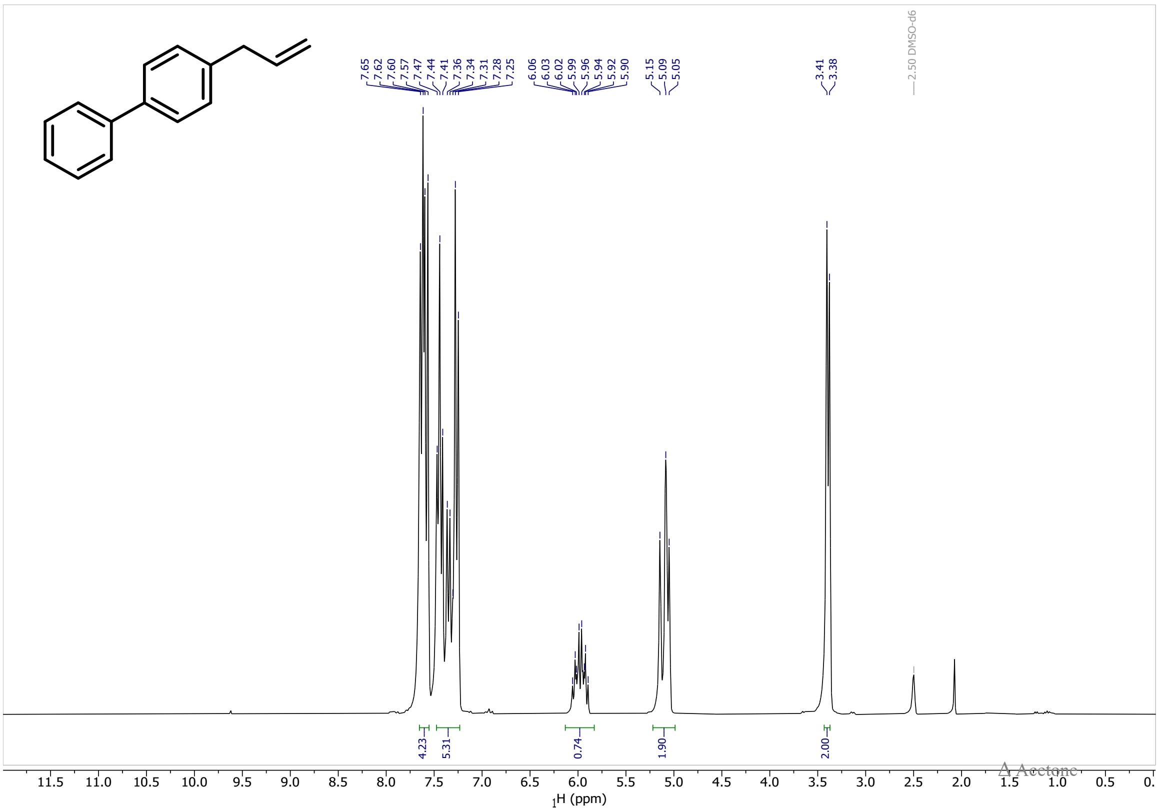

Figure S 29. ^{1}H NMR spectrum of synthesized substrate **9**.

1-Allyl-4-methylbenzene (7)

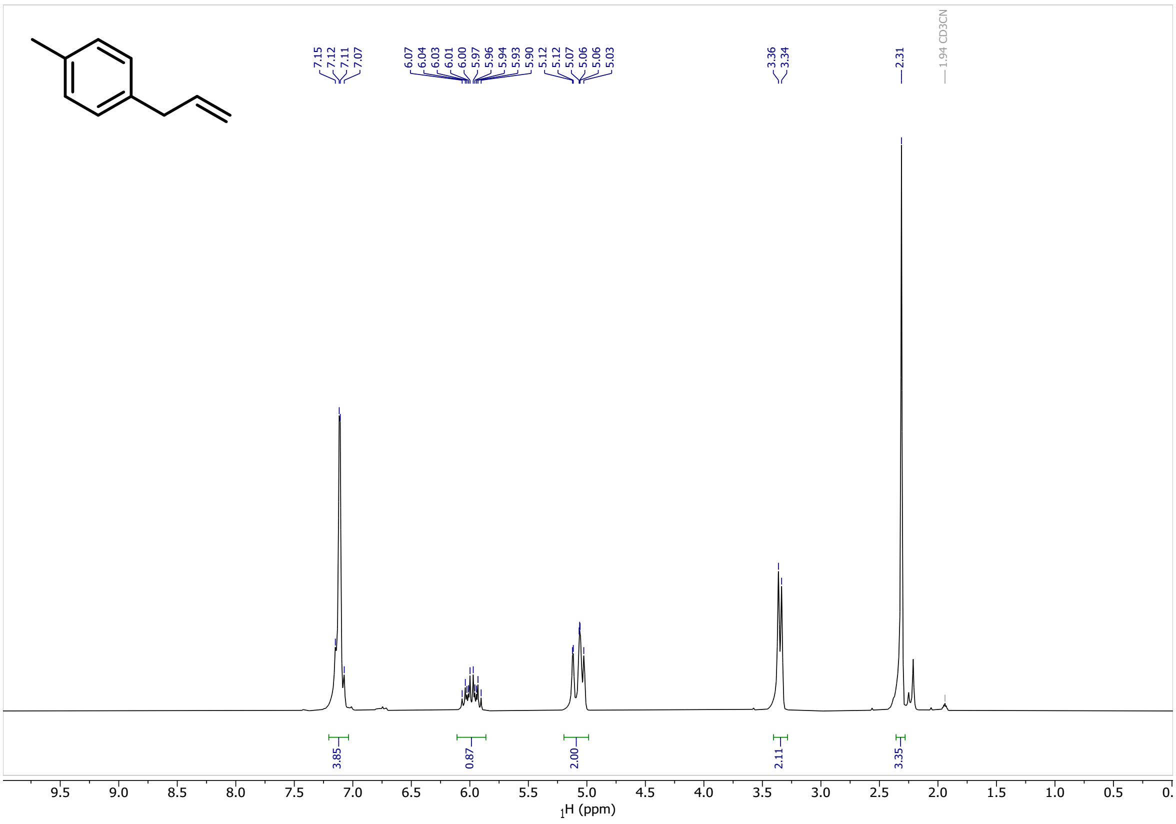

Figure S 30. ^{1}H NMR spectrum of synthesized substrate **7**.

1-Allyl-4-(^{t}Bu)benzene (10)

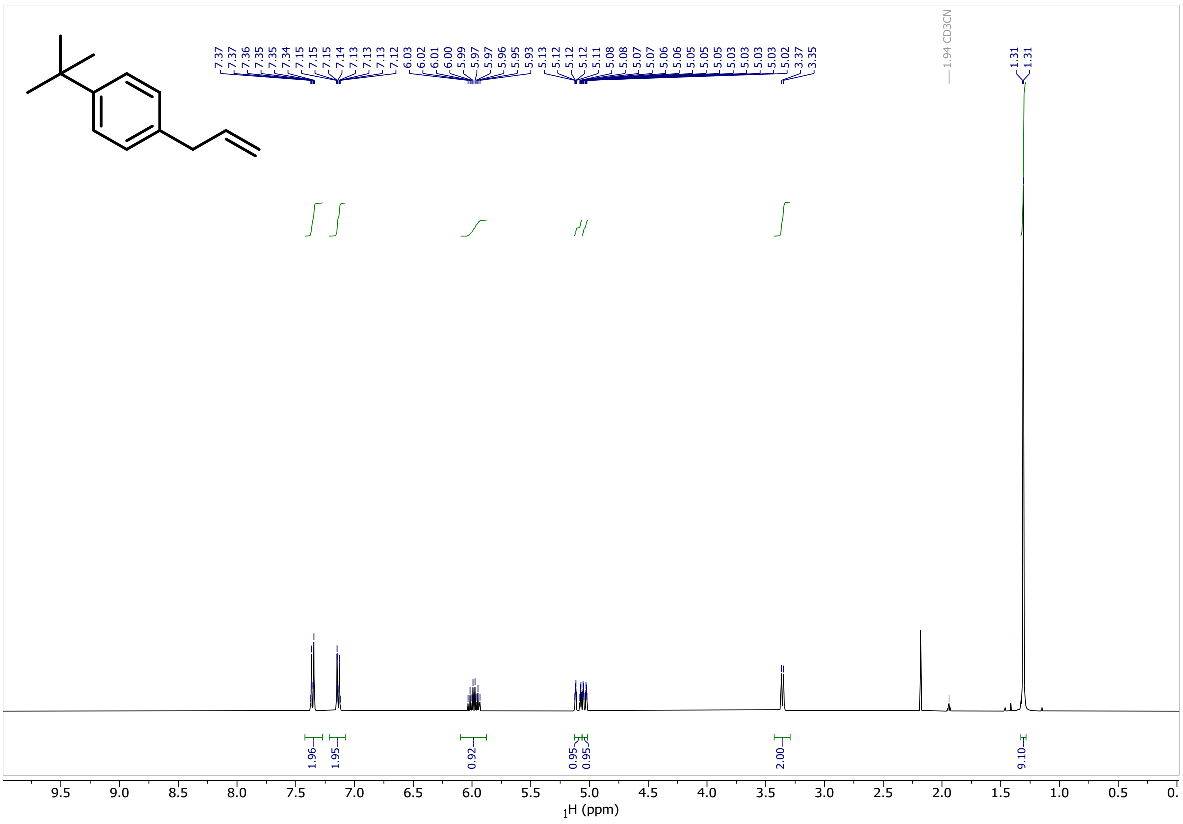

Figure S 31. ^{1}H NMR spectrum of synthesized substrate **10**.

2-Allyl-1,3,5-trimethylbenzene (11)

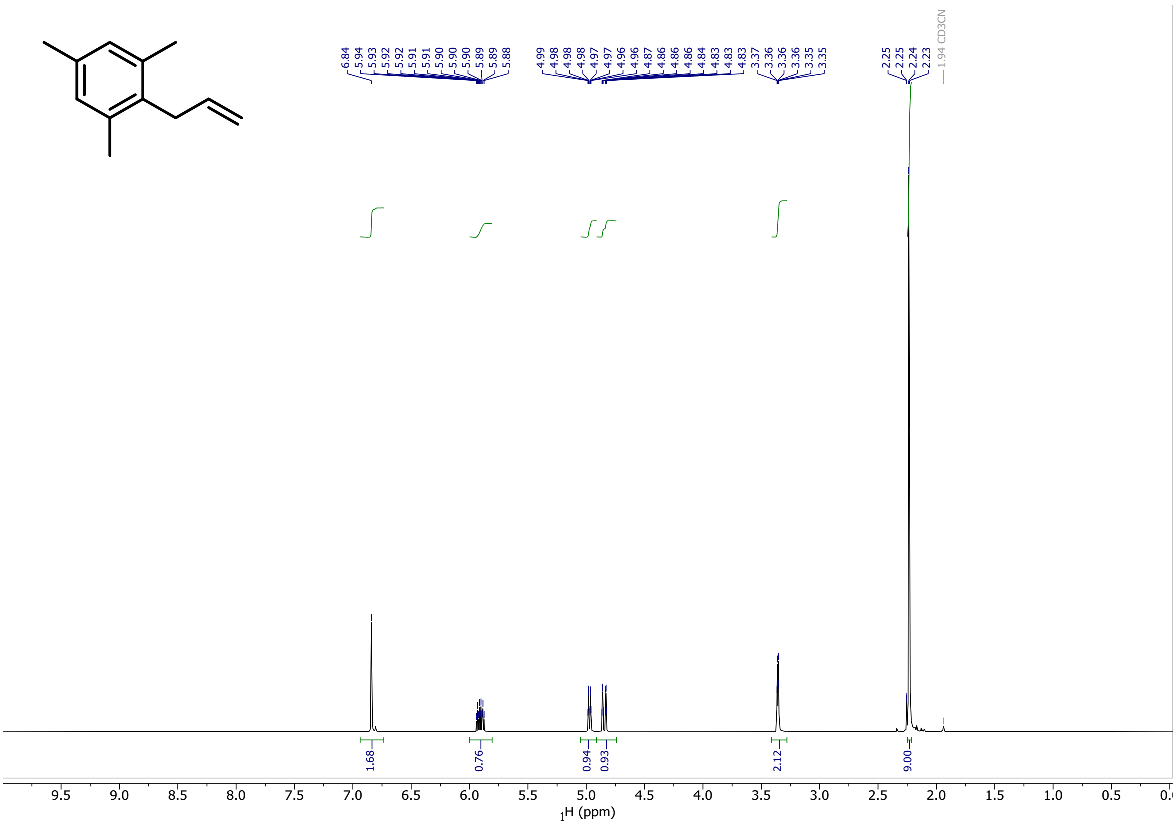

Figure S 32. ^{1}H NMR spectrum of synthesized substrate **11**.

1-Allyladamantane (20)

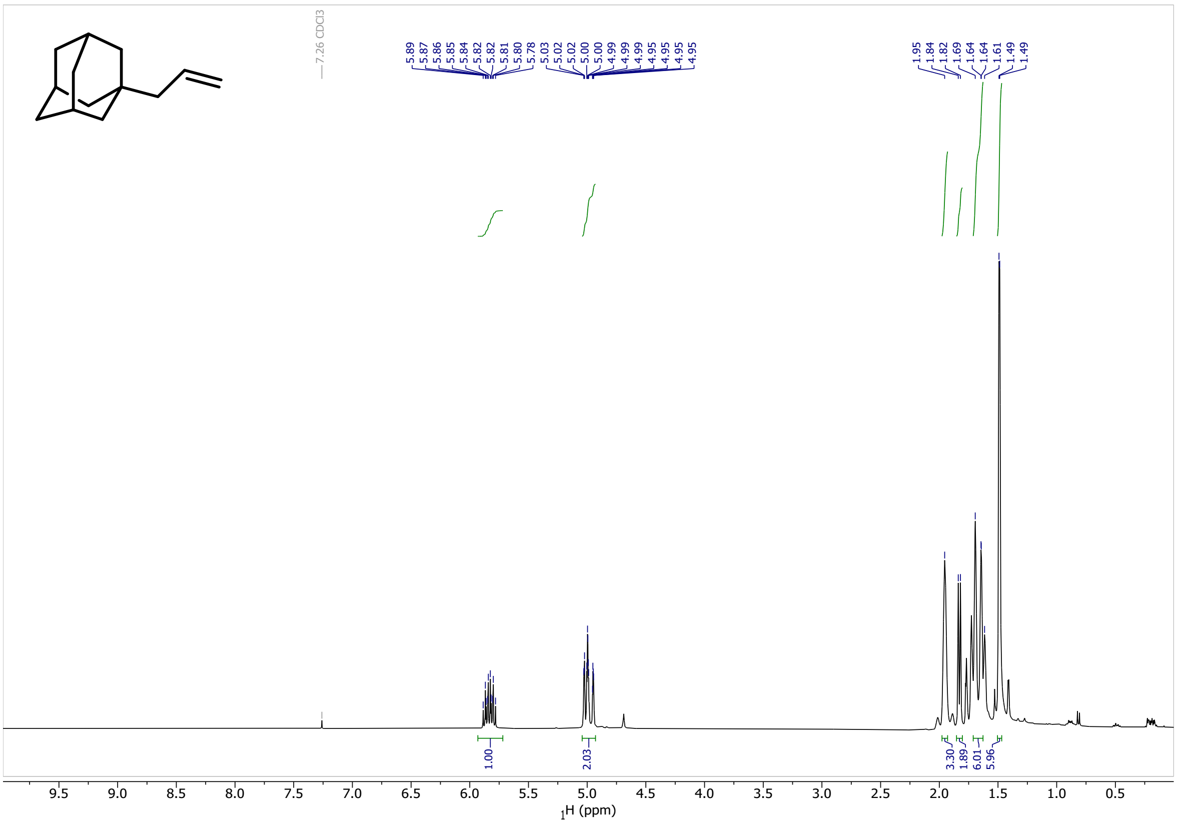

Figure S 33. ^{1}H NMR spectrum of synthesized substrate **20**.

(2-Allylphenoxy)trimethylsilane (3)

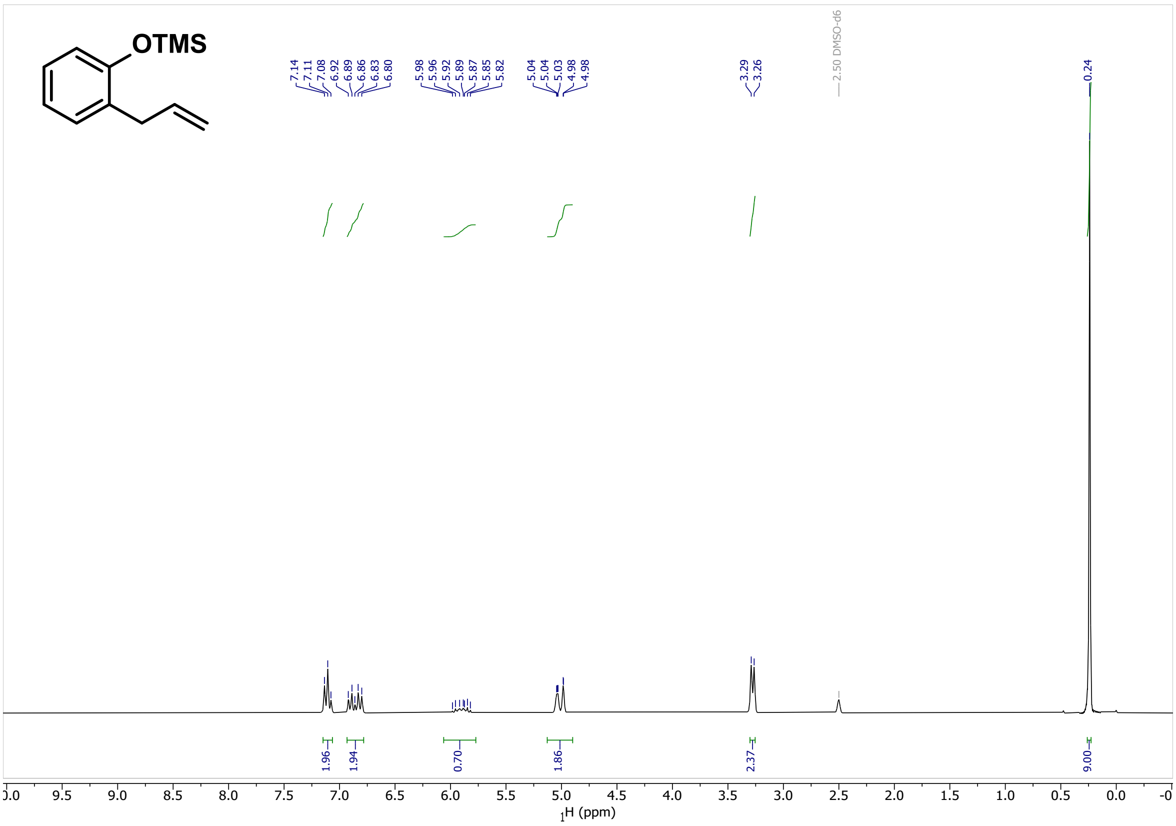

Figure S 34. ^{1}H NMR spectrum of synthesized substrate **3**.

(Hex-5-en-1-yloxy)trimethylsilane (16)

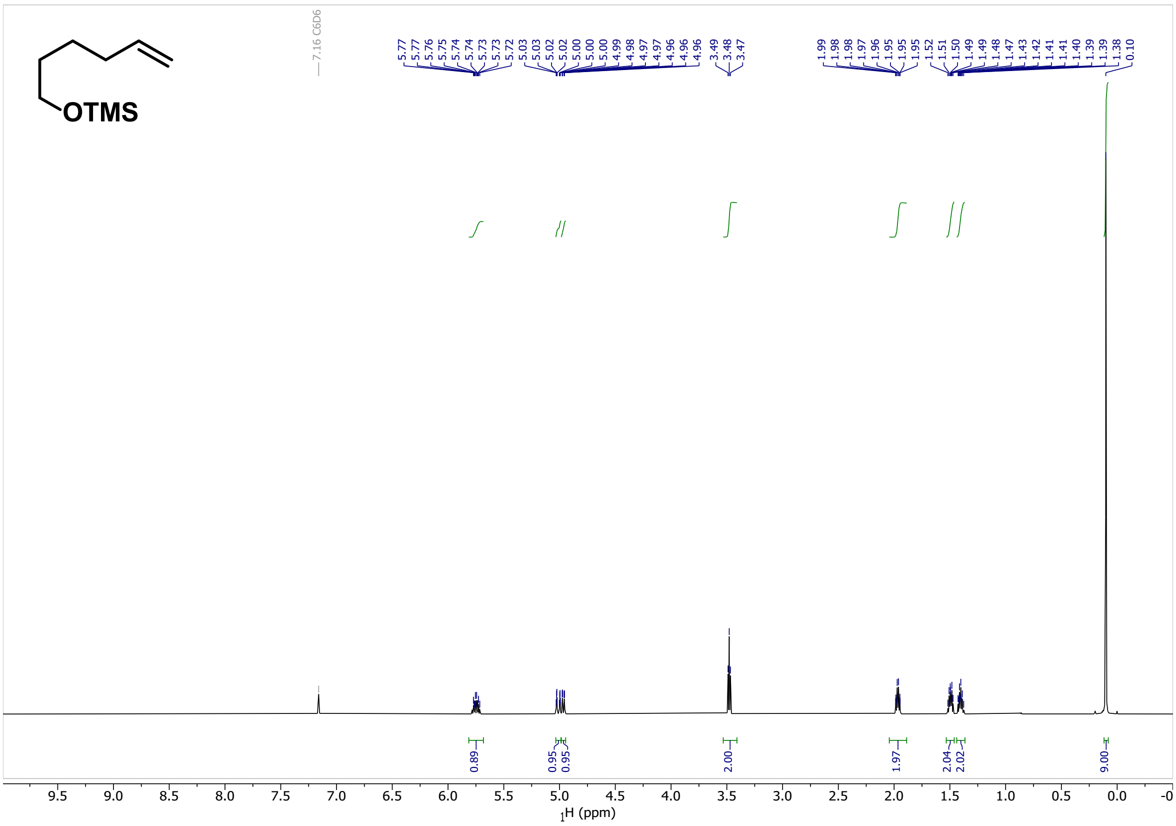

Figure S 35. ^{1}H NMR spectrum of synthesized spectrum **16**.

Trimethyl(pent-1-en-3-yloxy)silane (19)

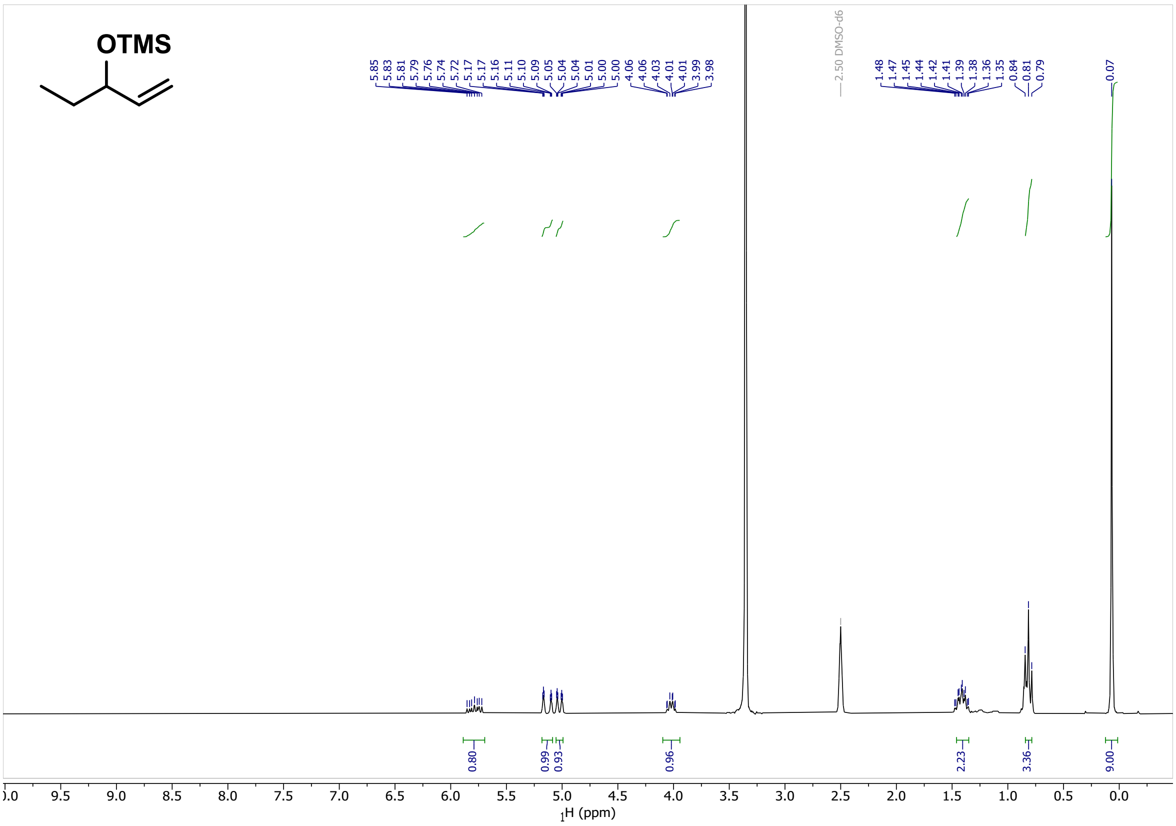

Figure S 36. ^{1}H NMR spectrum of synthesized substrate **19**.

Pent-4-en-2-ylbenzene (13)

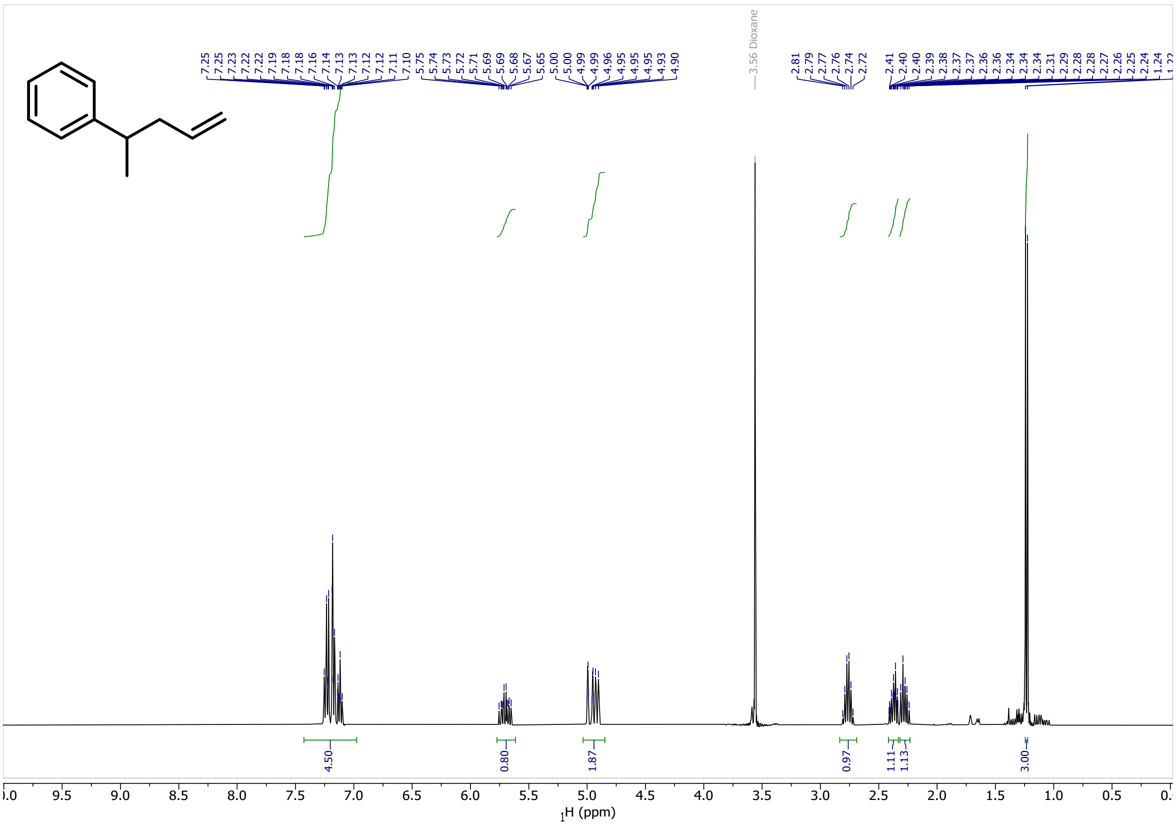

Figure S 37. ^{1}H NMR spectrum of synthesized substrate **13**.

(*Z*)-pent-3-en-1-ylbenzene (24)

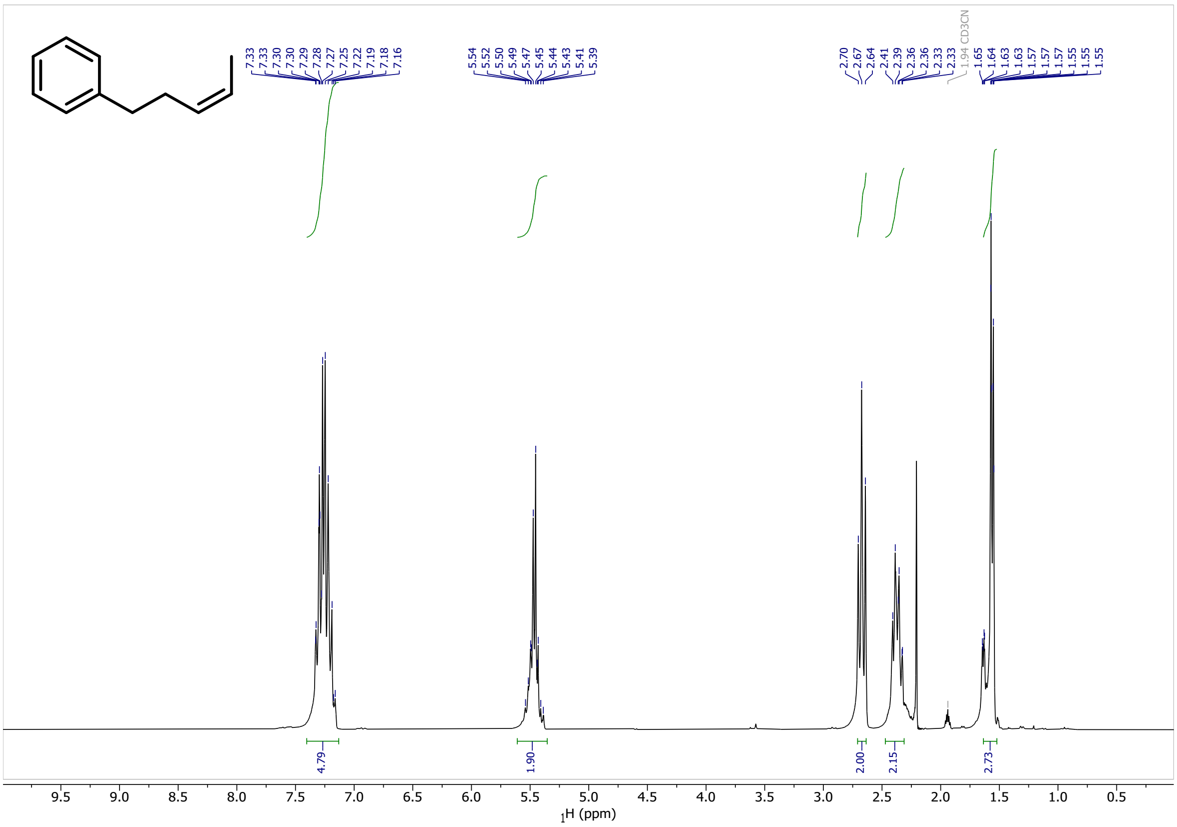

Figure S 38. ^{1}H NMR spectrum of synthesized substrate **24**.

2-Allylfuran (15)

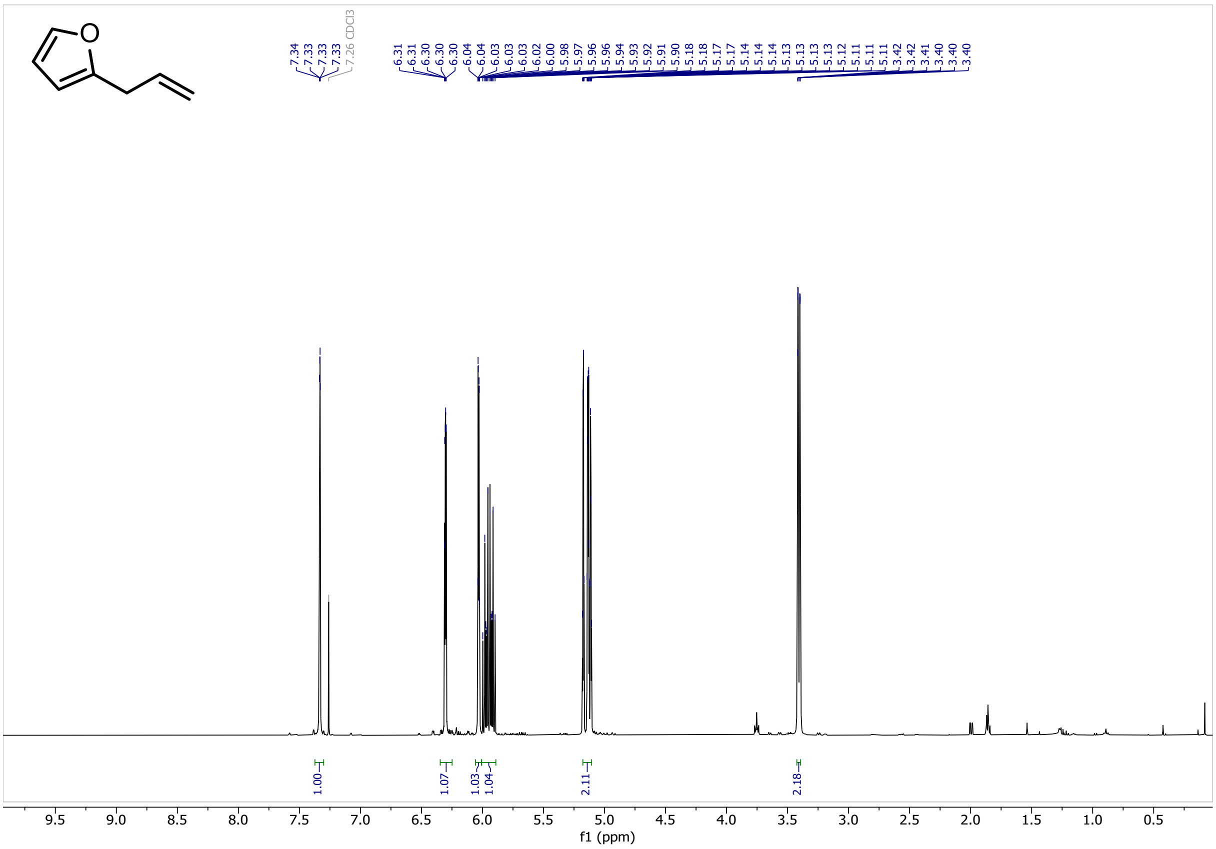

Figure S 39. ^{1}H NMR spectrum of synthesized substrate **14**.

Allylbenzene-2,2-*d₂* (2-*d₂*)

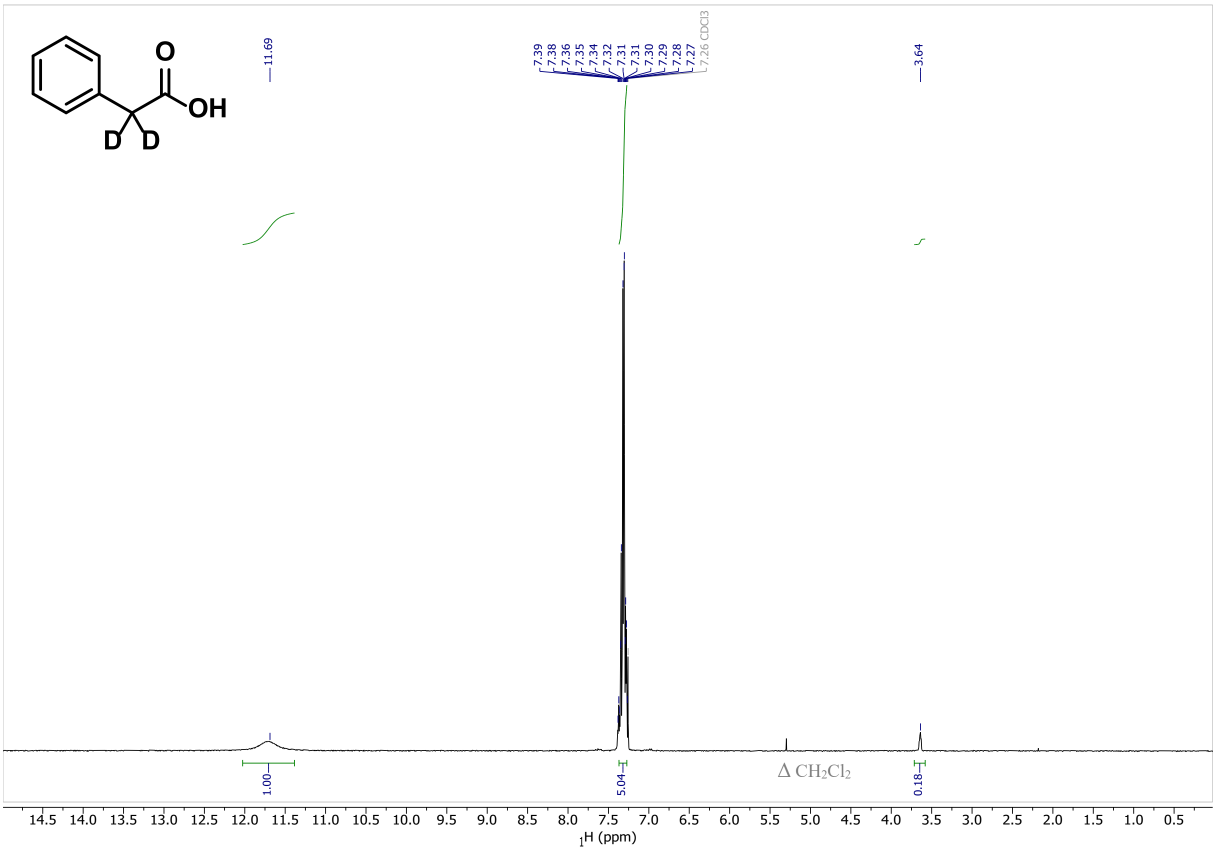

Figure S 40. ^{1}H NMR spectrum of phenylacetic acid-d$_2$.

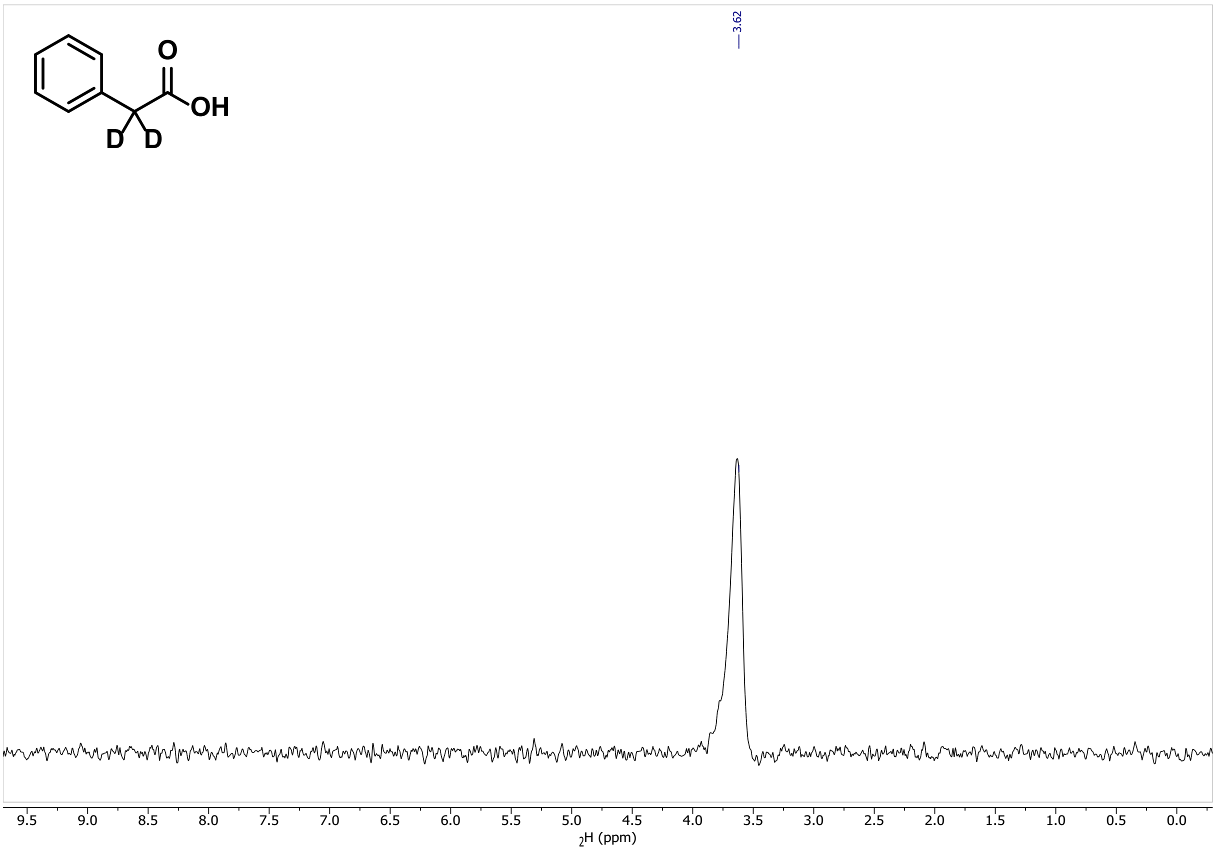

Figure S 41. ^{2}H NMR spectrum of phenylacetic acid-d$_2$.

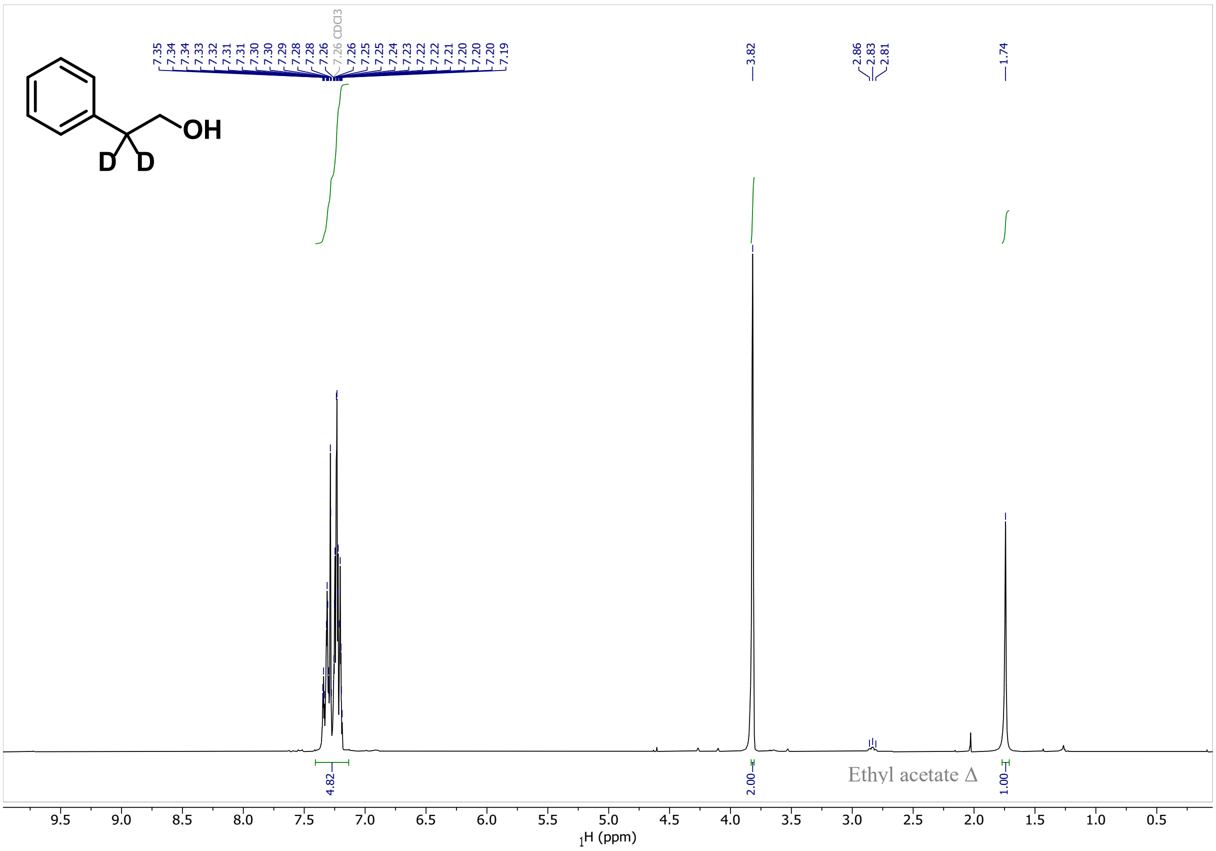

Figure S 42. ^{1}H NMR spectrum of phenylethanol-2,2-d$_2$.

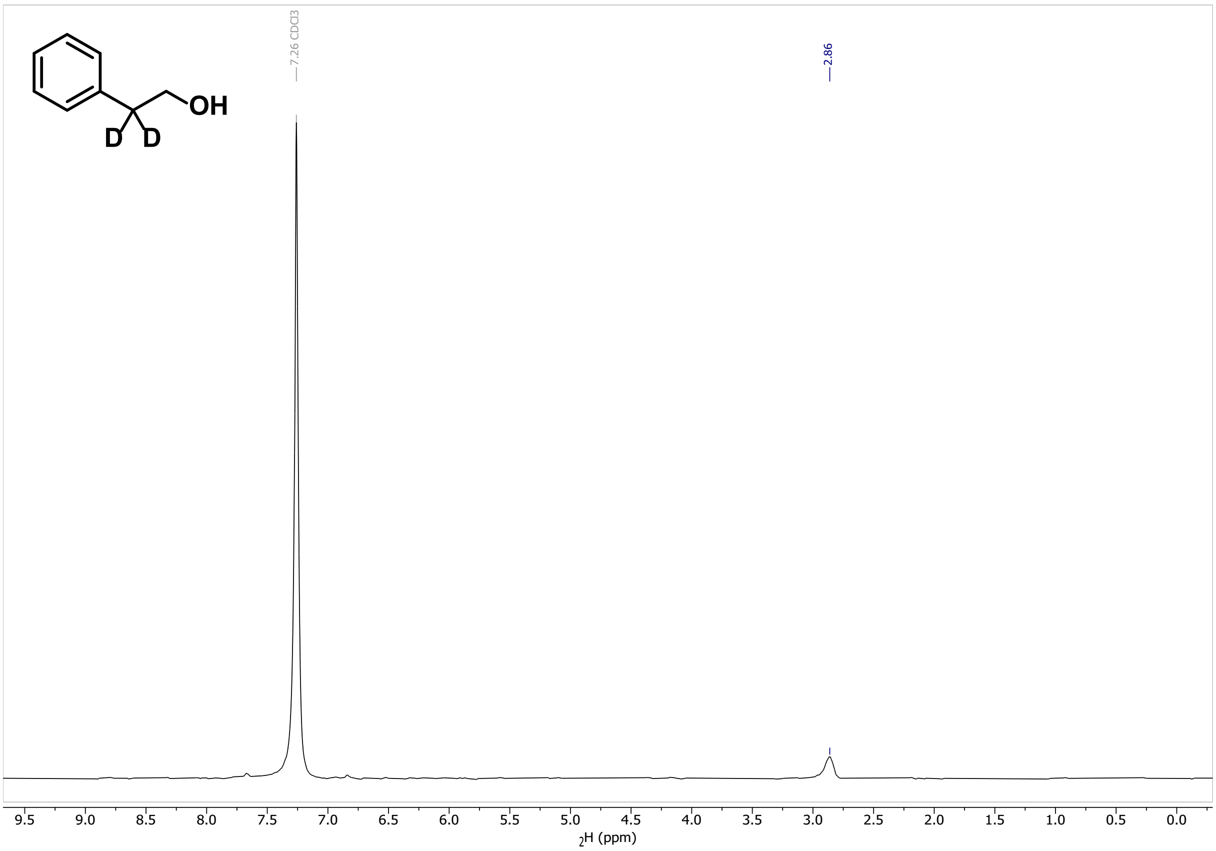

Figure S 43. ^{2}H NMR spectrum of phenylethanol-2,2-d$_2$.

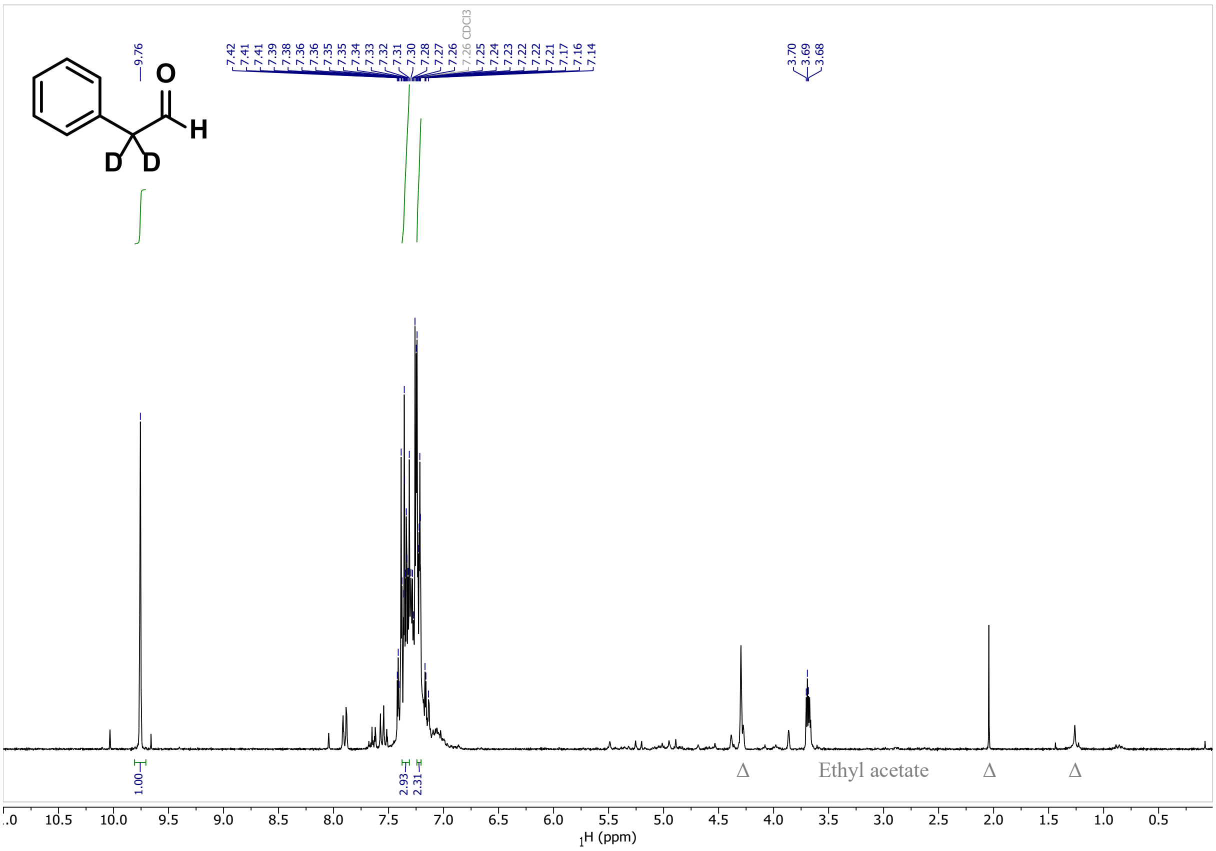

Figure S 44. ^{1}H NMR spectrum of phenylacetaldehyde-2,2-d$_2$.

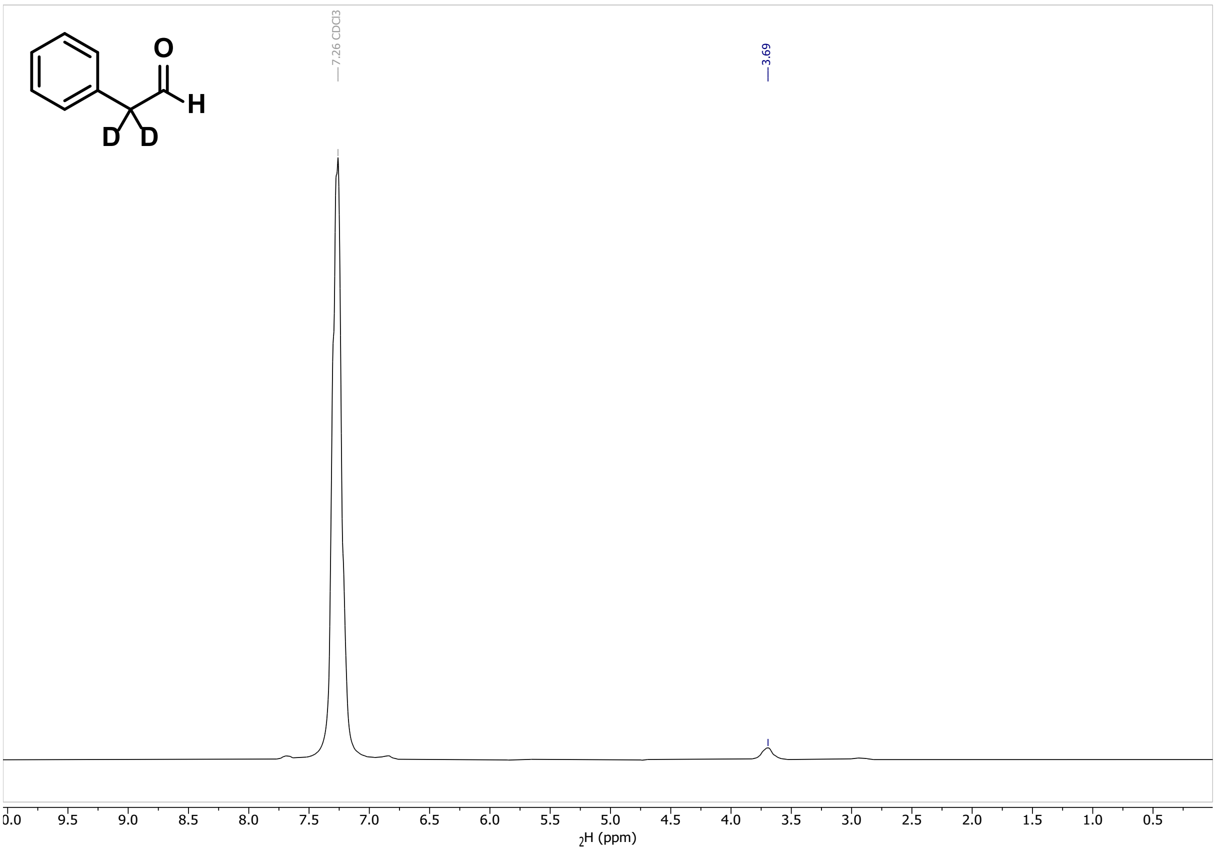

Figure S 45. ^{2}H NMR spectrum of phenylacetaldehyde-2,2-d$_2$.

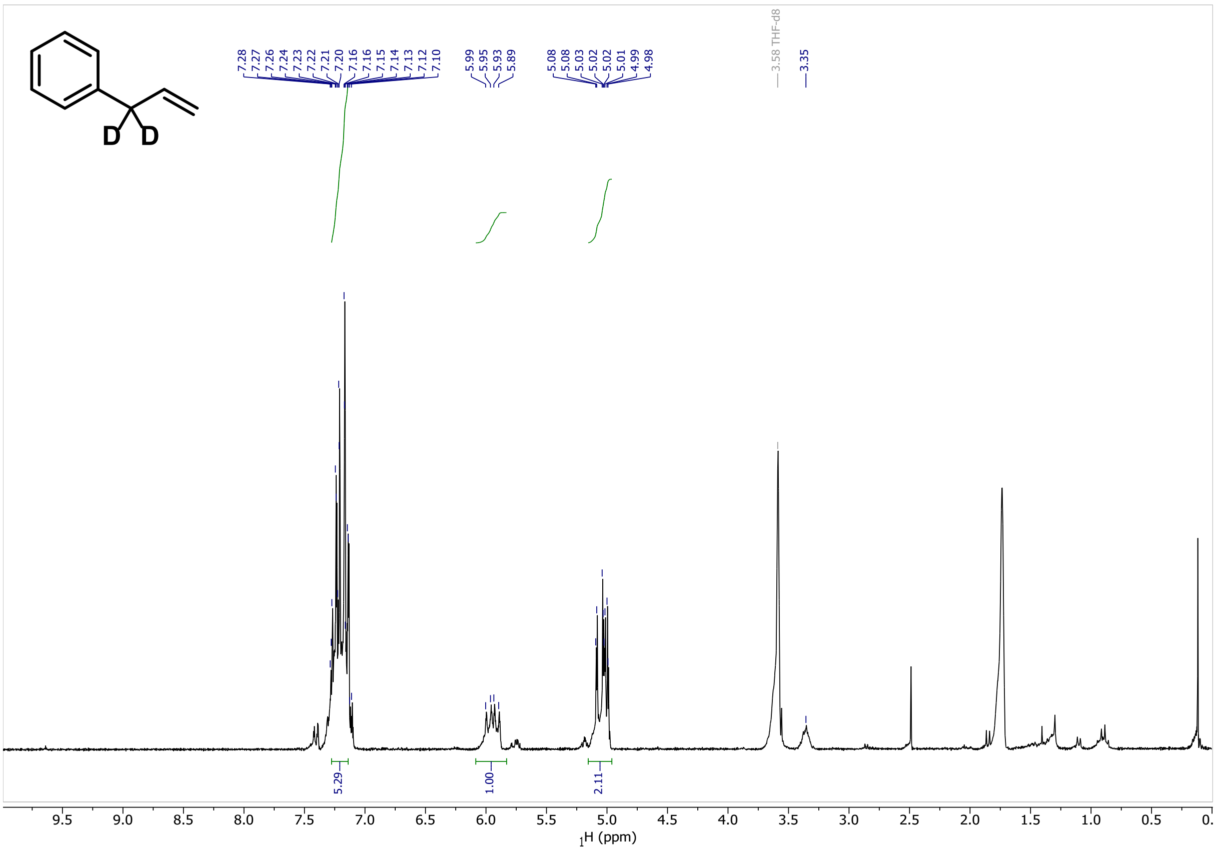

Figure S 46. ^{1}H NMR spectrum of allylbenzene-2,2-d$_2$ (**2-d$_2$**).

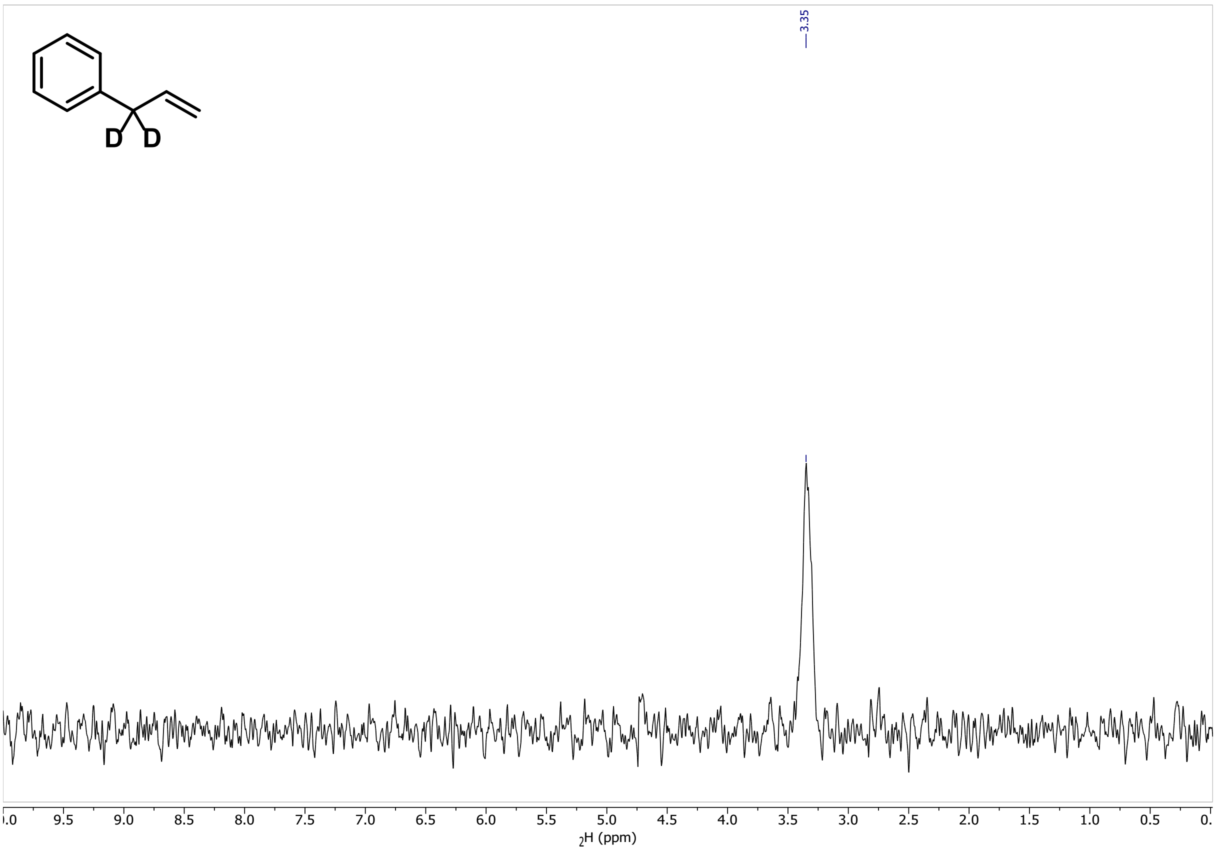

Figure S 47. ^{2}H NMR spectrum of allylbenzene-2,2-d$_2$ (**2-d$_2$**).

5.3 NMR Spectra of One-Bond Isomerization Products

Note: For product mixtures with a significant amount of undesired isomer (≥ 15 %), starting material or chain-walking product, characteristic signals are labelled in the spectrum. NMR spectra recorded from the reaction solution contain 0.013 mmol of 1,4-dioxane (0.25 equiv. in respect to the substrate) as internal standard.

(*E*)-1-Fluoro-4-(prop-1-en-1-yl)benzene (1a)

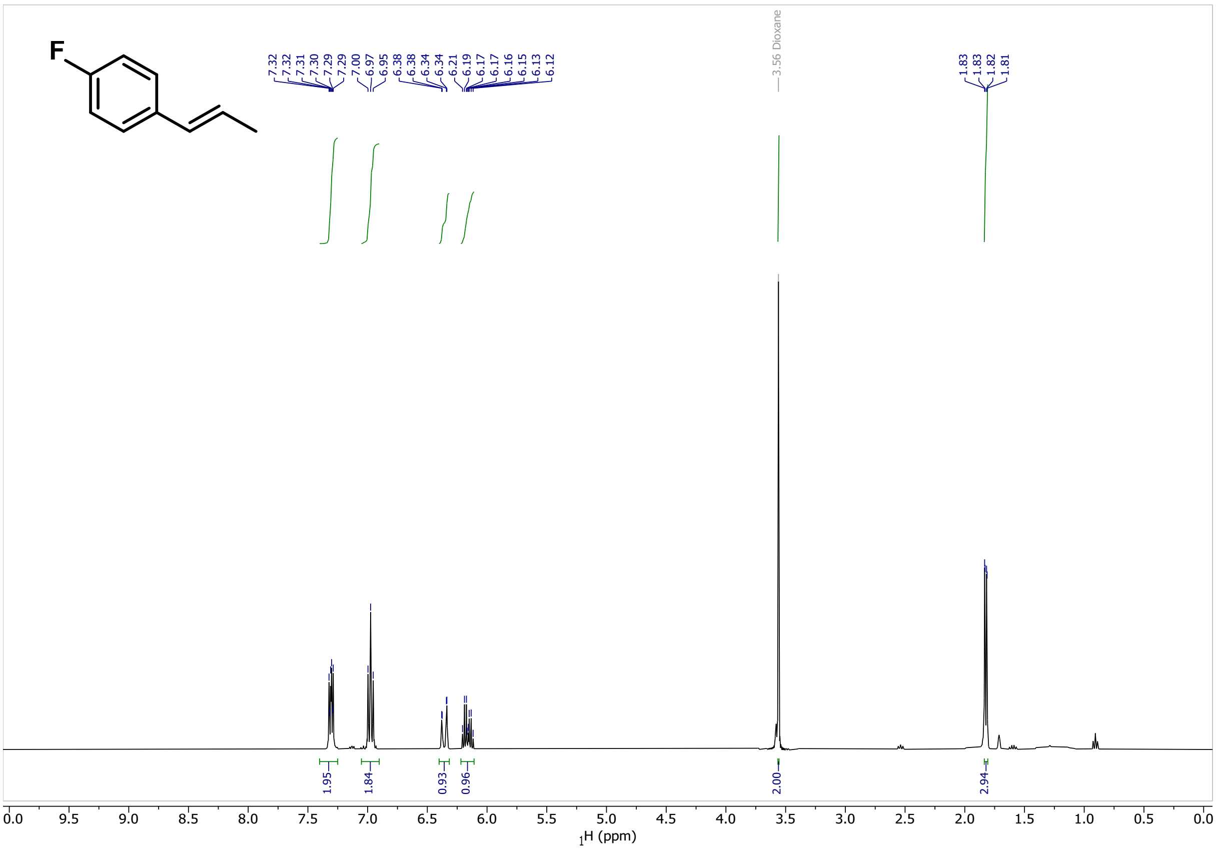

Figure S 48. ^{1}H NMR spectrum of **1a**.

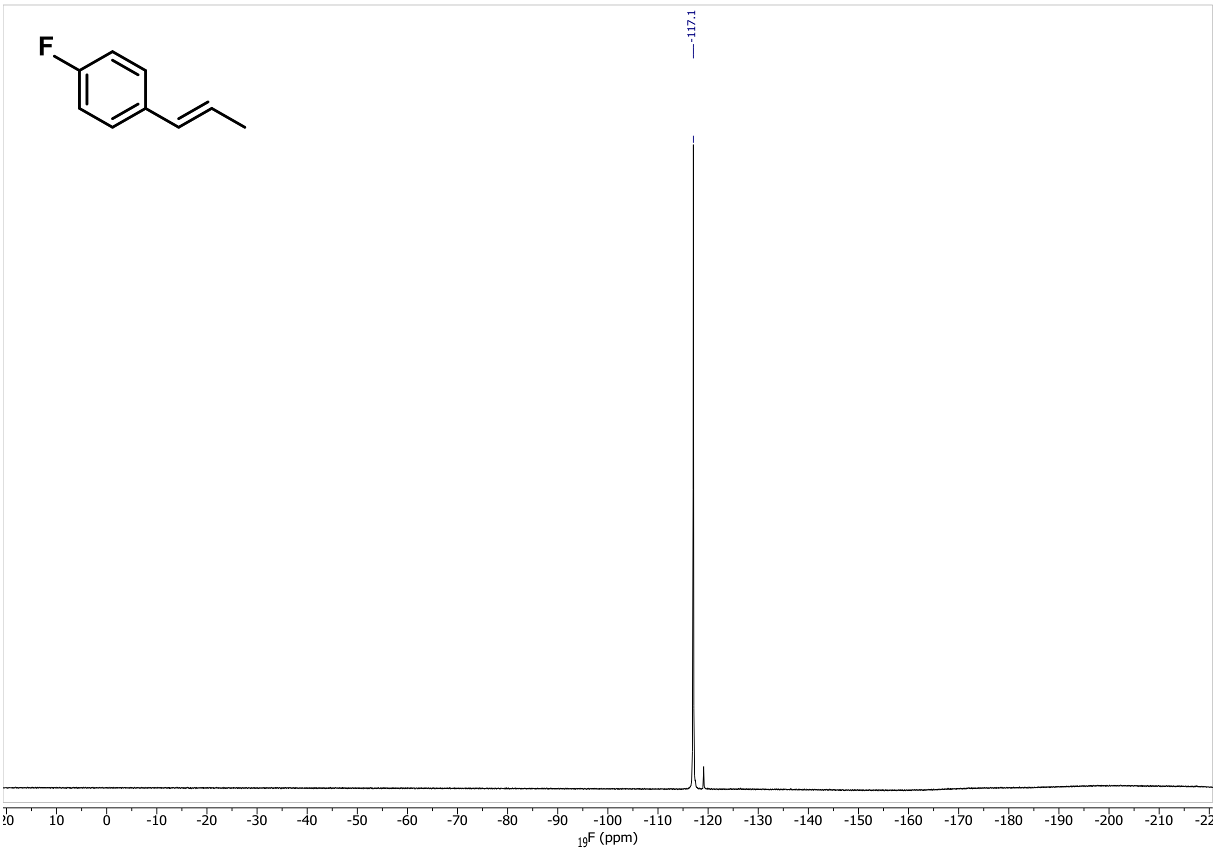

Figure S 49. ^{19}F NMR spectrum of **1a**.

(*E*)-Prop-1-en-1-ylbenzene (2a)

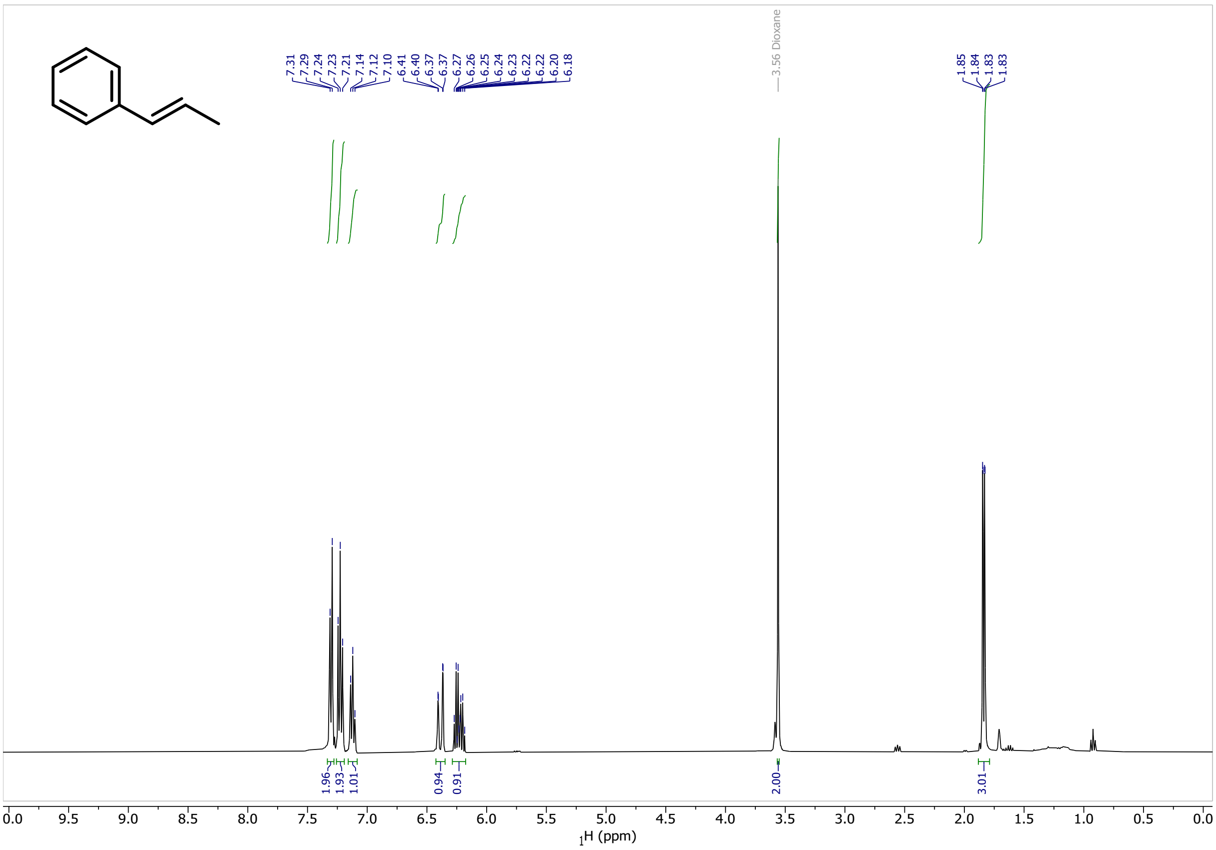

Figure S 50. ^{1}H NMR spectrum of **2a**.

***(E)*-(2-Allylphenoxy)trimethylsilane (3a)**

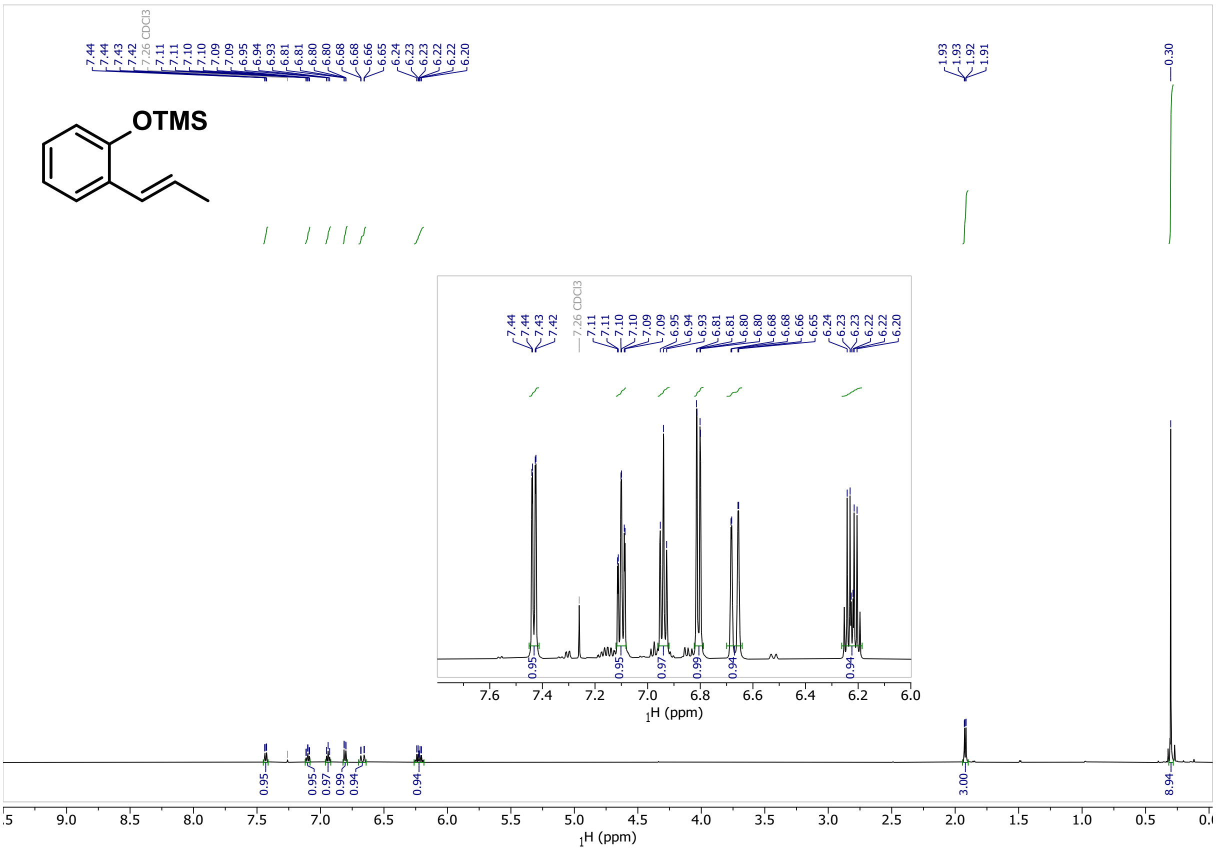

Figure S 51. ^{1}H NMR spectrum of **3a** (detailed view of the double-bond and aromatic region).

***(E)*-Anethole (4a)**

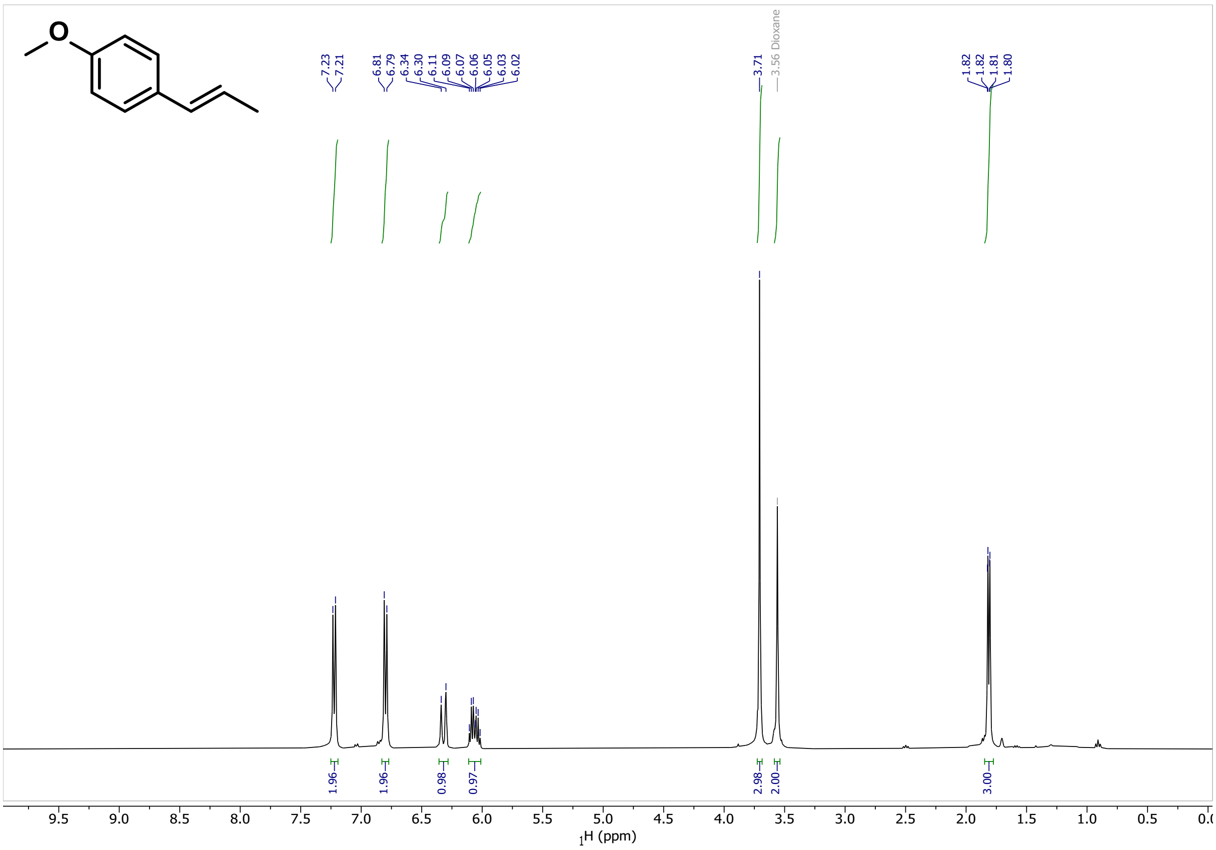

Figure S 52. ^{1}H NMR spectrum of **4a**.

1,2,3,4,5-Pentafluoro-6-(prop-1-en-1-yl)benzene (5a)

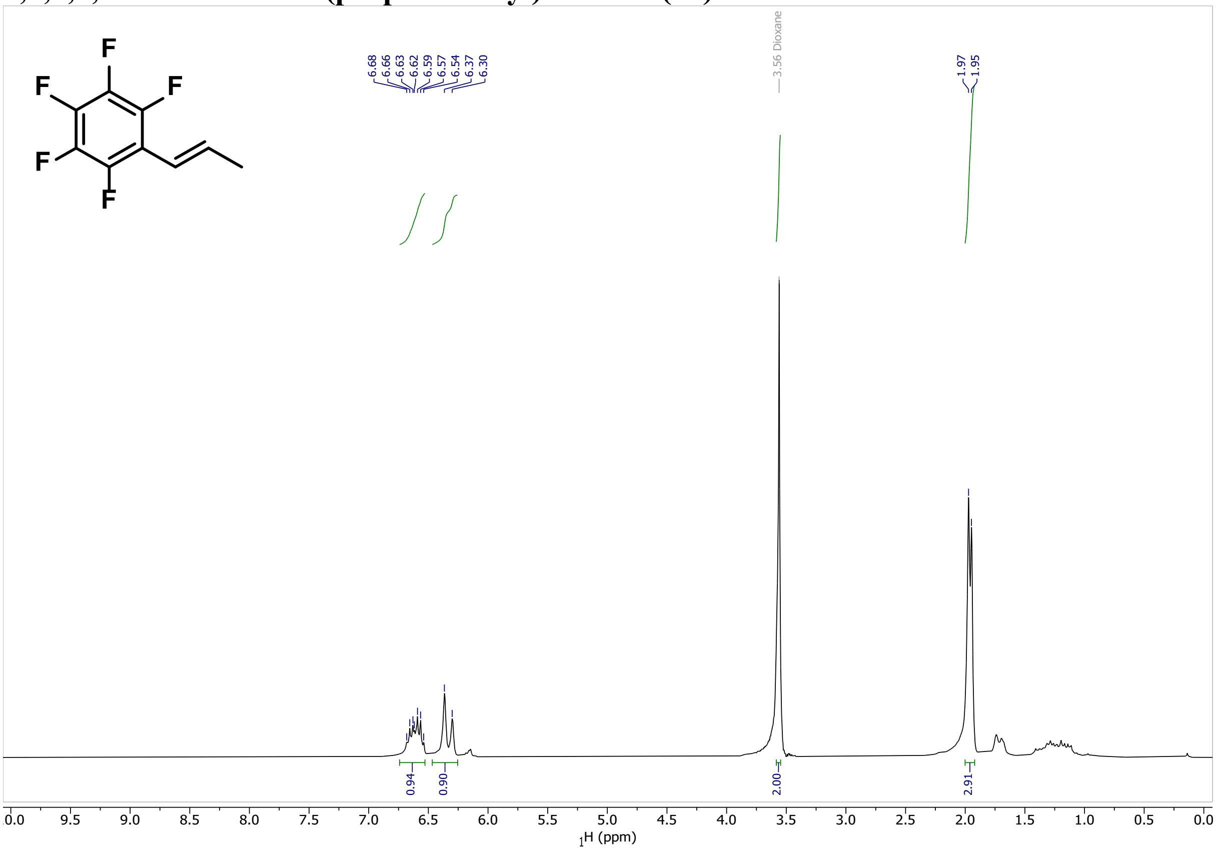

Figure S 53. ^{1}H NMR spectrum of **5a**.

(*E*)-1-(prop-1-en-1-yl)naphthalene (6a)

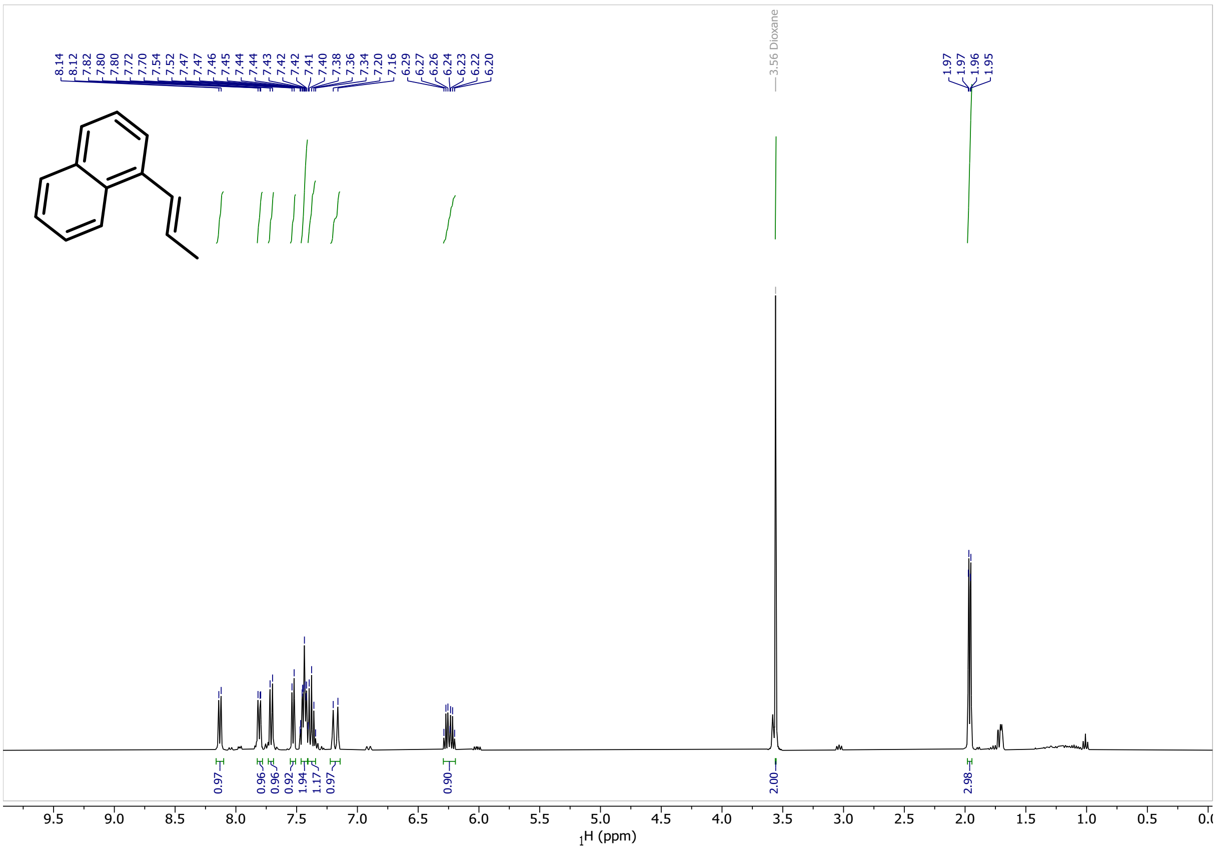

Figure S 54. ^{1}H NMR spectrum of **6a**.

***(E)*-1-Methyl-4-(prop-1-en-1-yl)benzene (7a)**

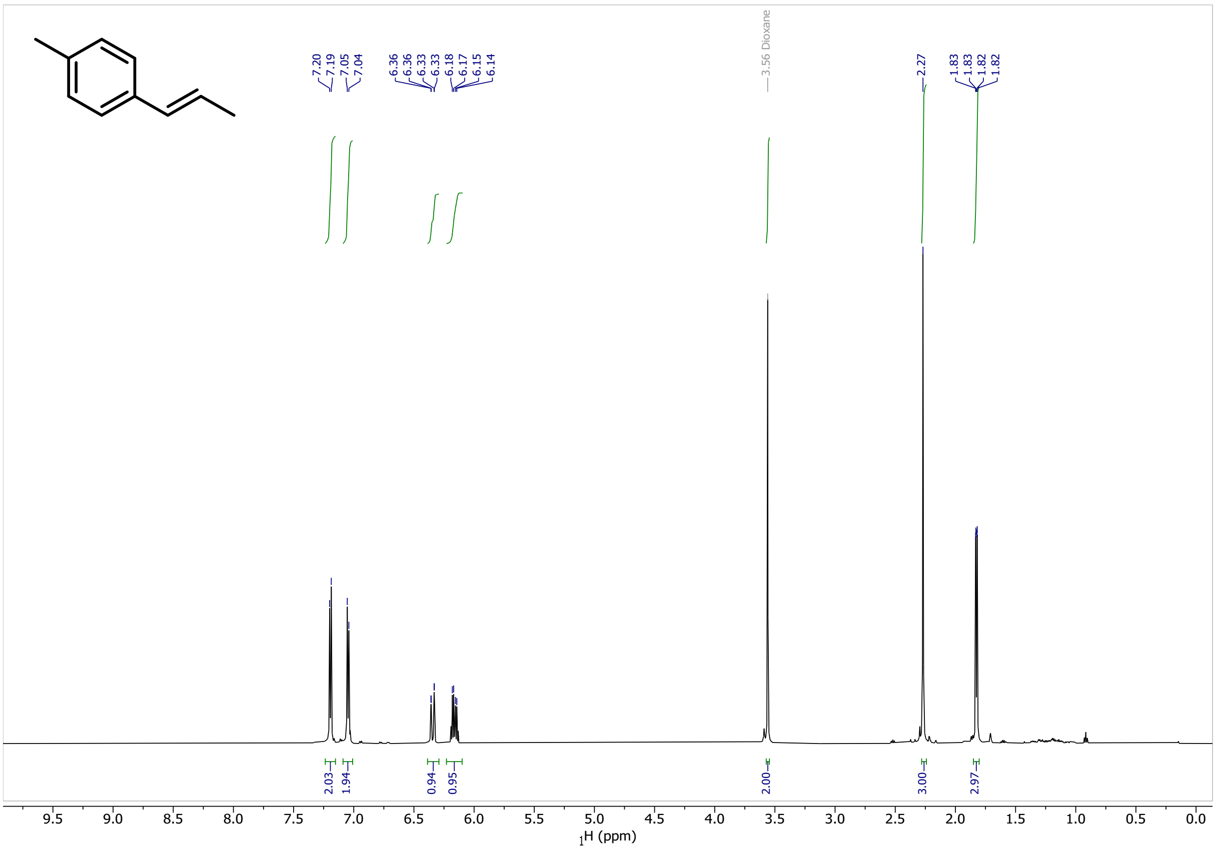

Figure S 55. ^{1}H NMR spectrum of **7a**.

***(E)*-2-Methyl-4-(prop-1-en-1-yl)benzene (8a)**

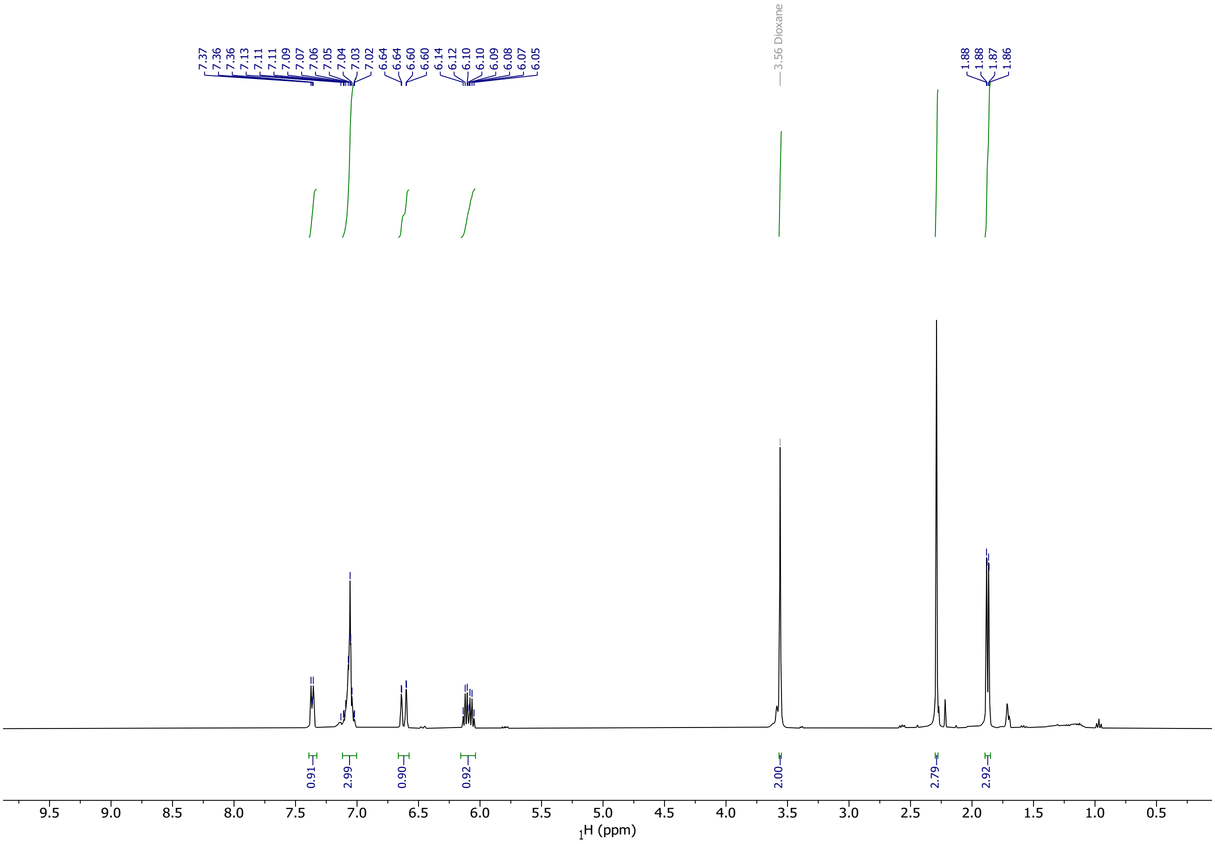

Figure S 56. ^{1}H NMR spectrum of **8a**.

***(E)*-4-(Prop-1-en-1-yl)-1,1'-biphenyl (9a)**

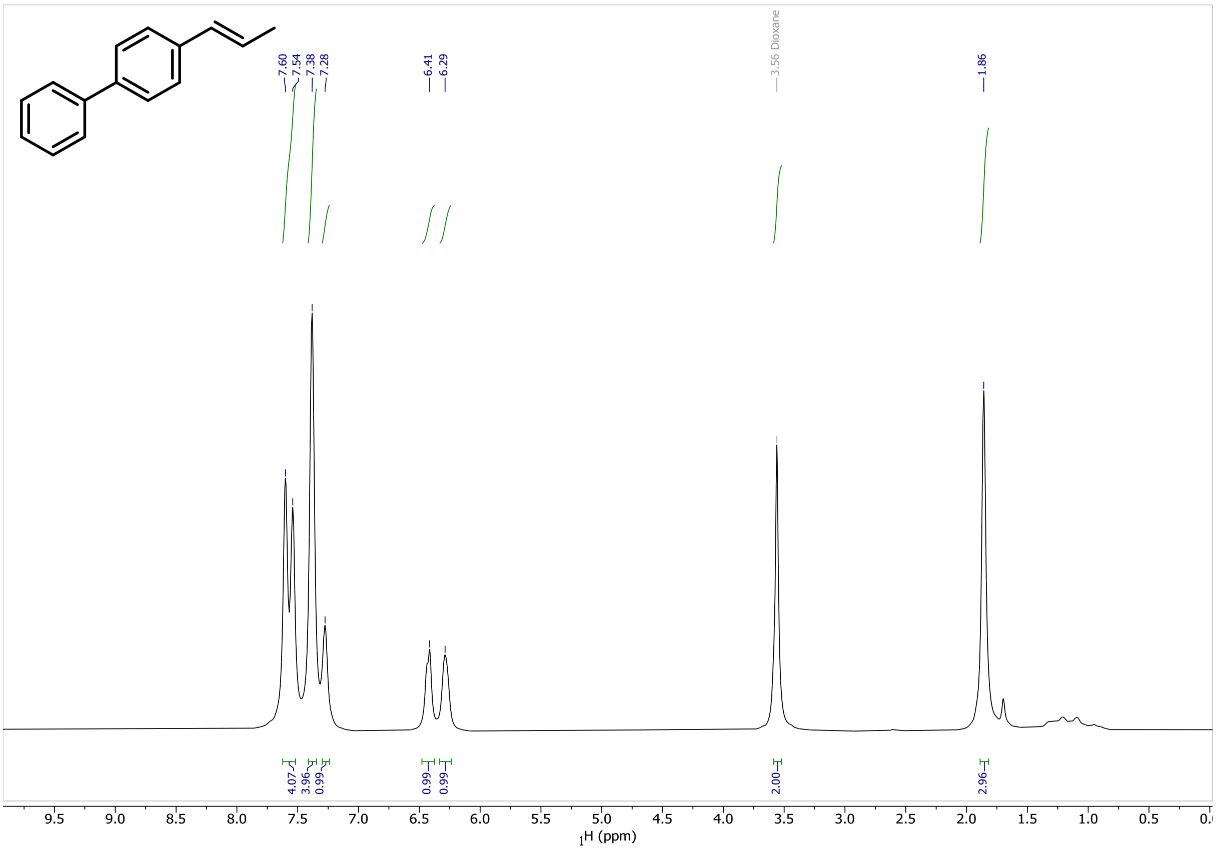

Figure S 57. ^{1}H NMR spectrum of **9a**.

***(E)*-1-(^{t}Bu)-4-(prop-1-en-1-yl)benzene (10a)**

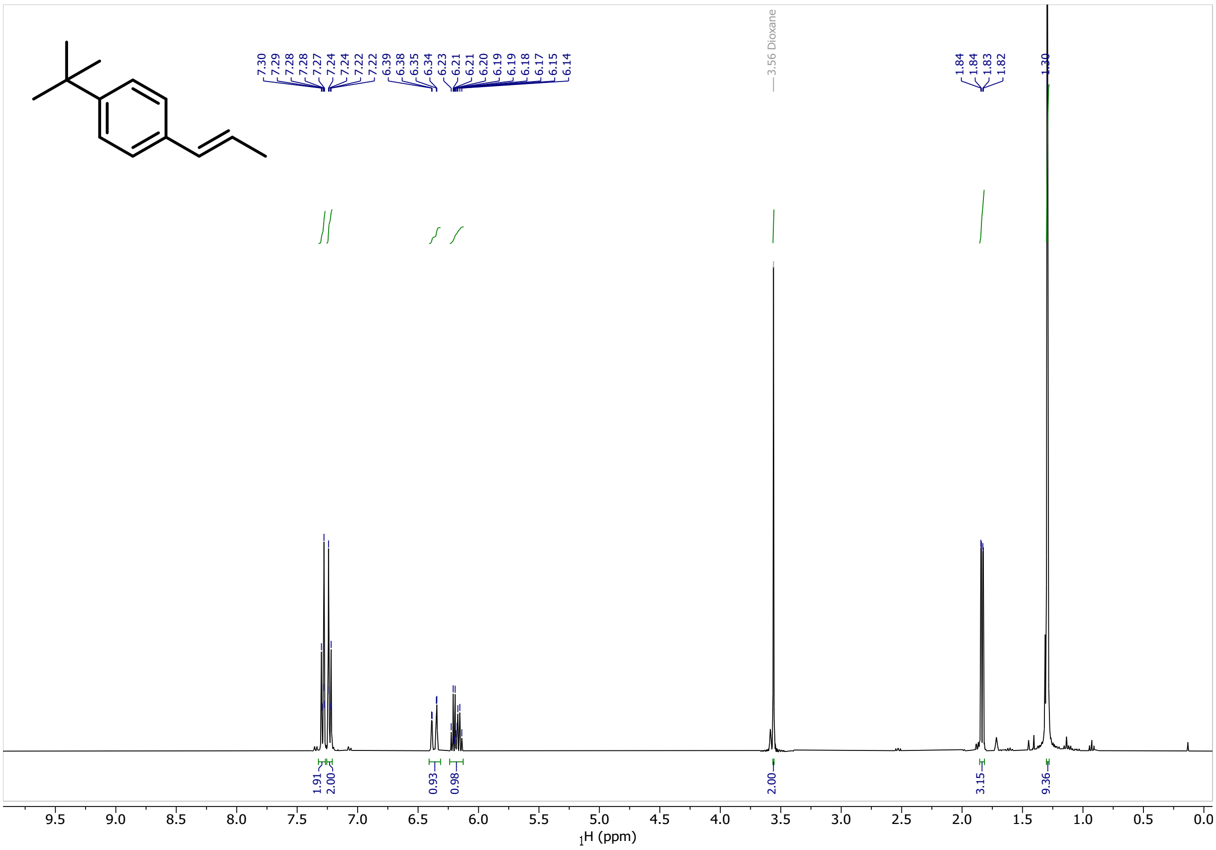

Figure S 58. ^{1}H NMR spectrum of **10a**.

(*E*)-1,3,5-Trimethyl-2-(prop-1-en-1-yl)benzene (11a)

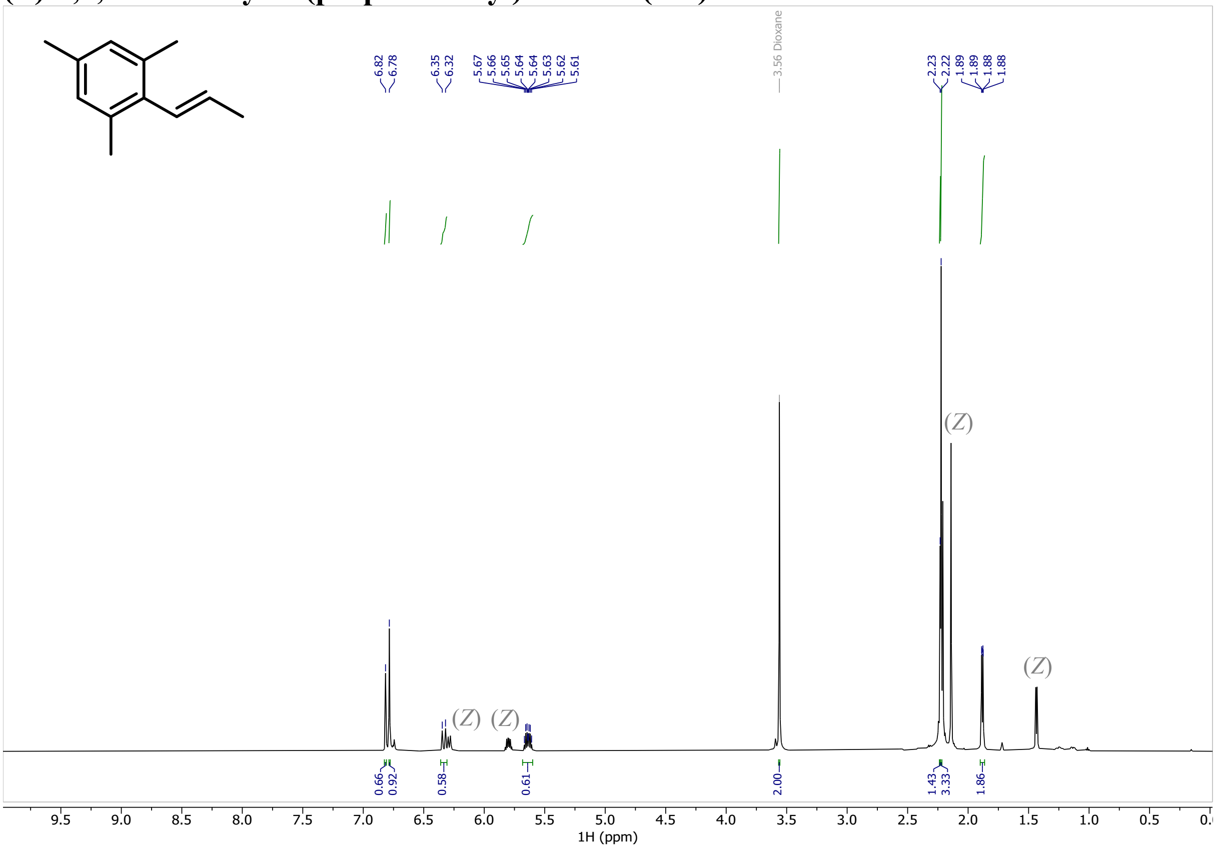

Figure S 59. ^{1}H NMR spectrum of **11a** (Z-isomer annotated).

(*E*)-But-2-en-1-ylbenzene (12a)

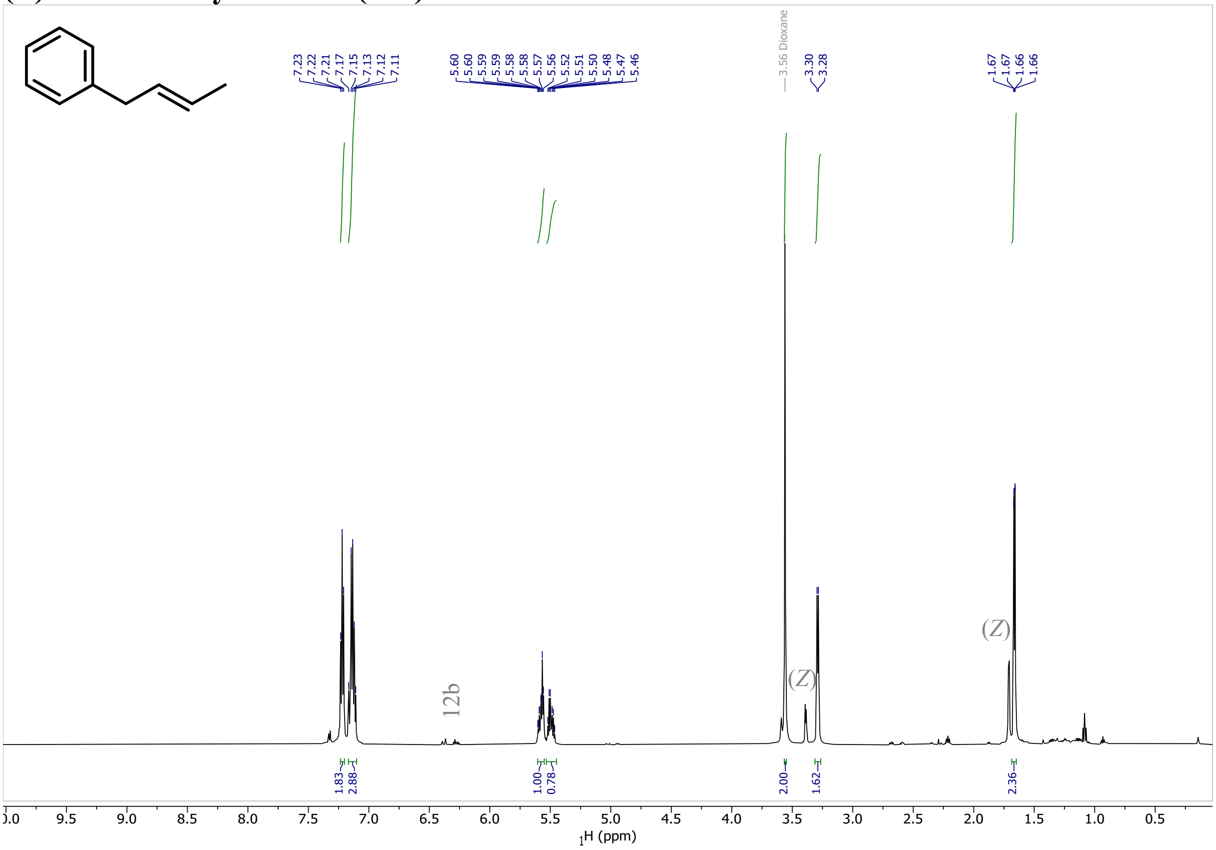

Figure S 60. ^{1}H NMR spectrum of **12a** (Z-isomer and chain-walking isomerization byproduct annotated).

***(E)*-Pent-3-en-2-ylbenzene (13a)**

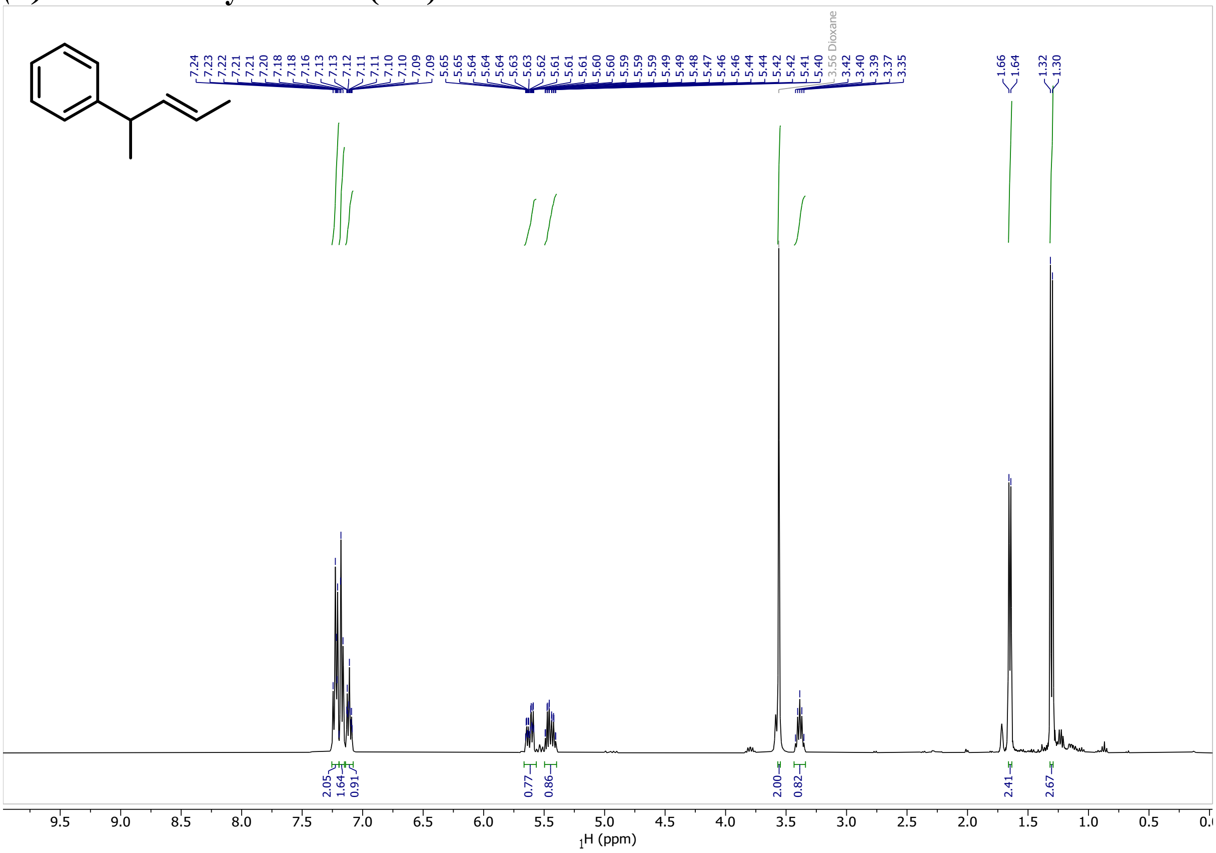

Figure S 61. ^{1}H NMR spectrum of **13a**.

(*E*)-2-(Prop-1-en-1-yl)thiophene (14a)

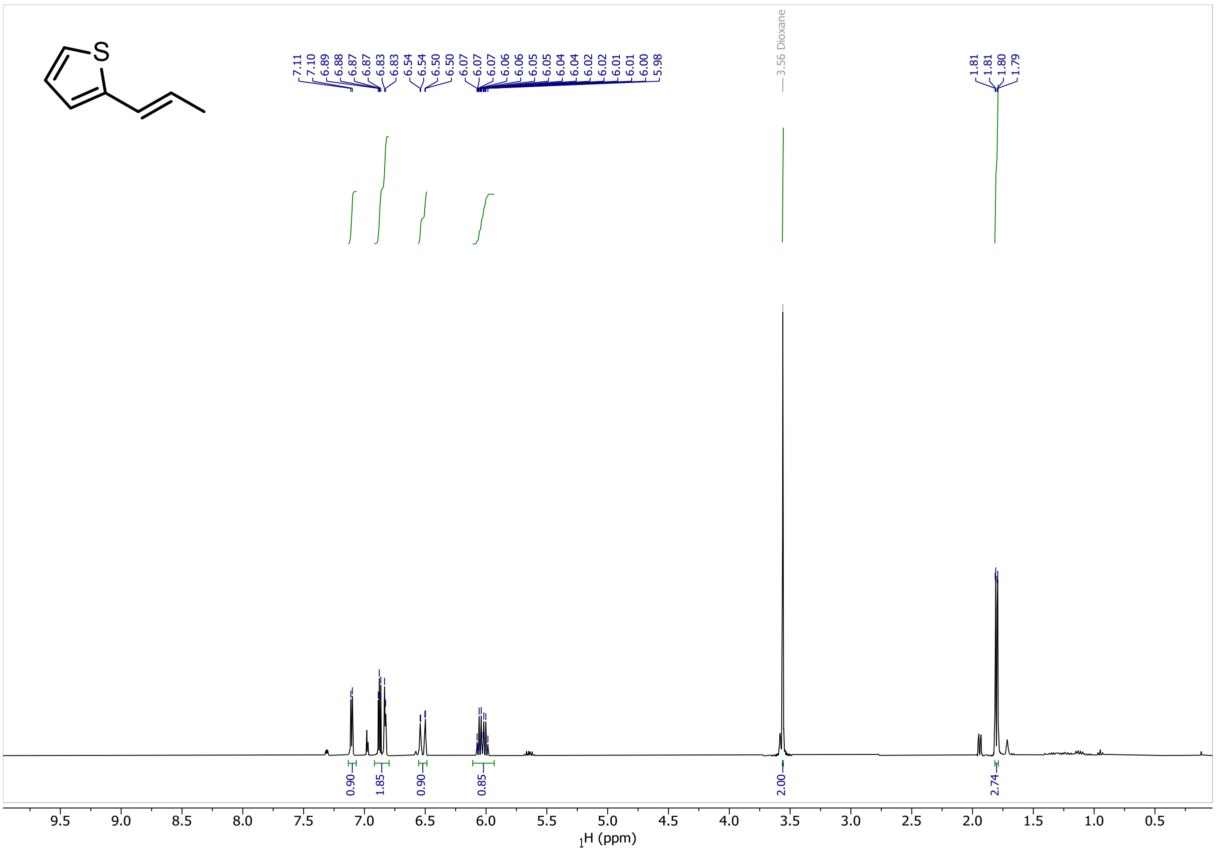

Figure S 62. ^{1}H NMR spectrum of **14a**.

(E)-2-(Prop-1-en-1-yl)furan (15a)

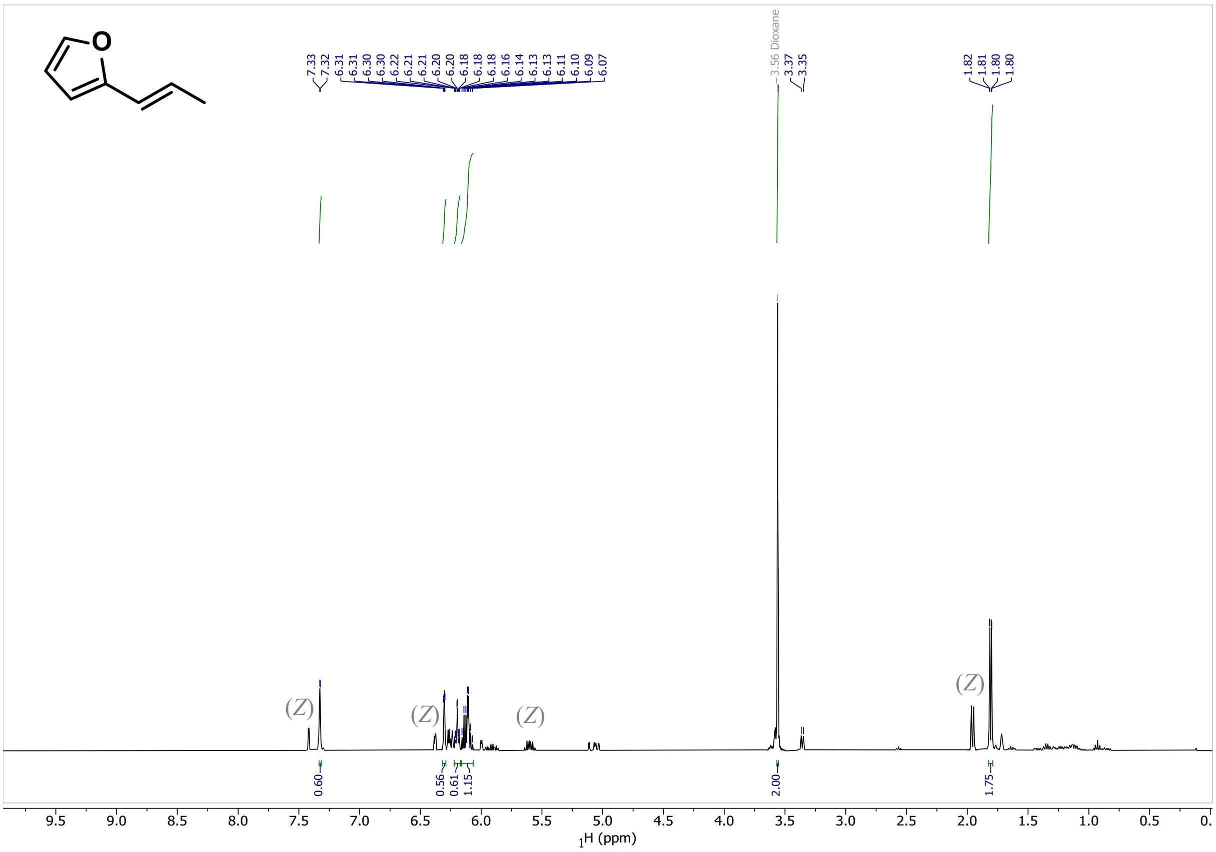

Figure S 63. ¹H NMR spectrum of **15a** (Z-isomer annotated).

(E)-(Hex-4-en-1-yloxy)trimethylsilane (16a)

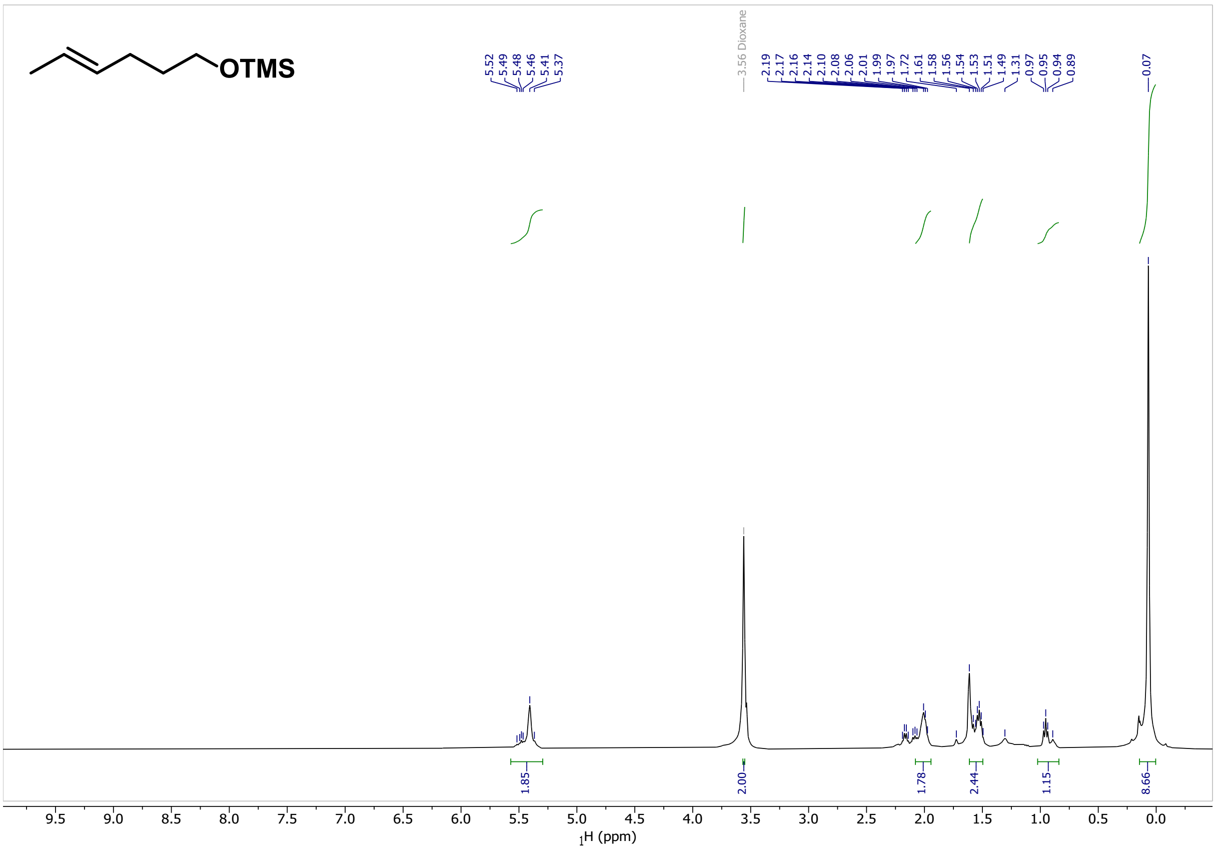

Figure S 64. ¹H NMR spectrum of **16a**.

(*E*)-6-Chlorohex-2-ene (17a)

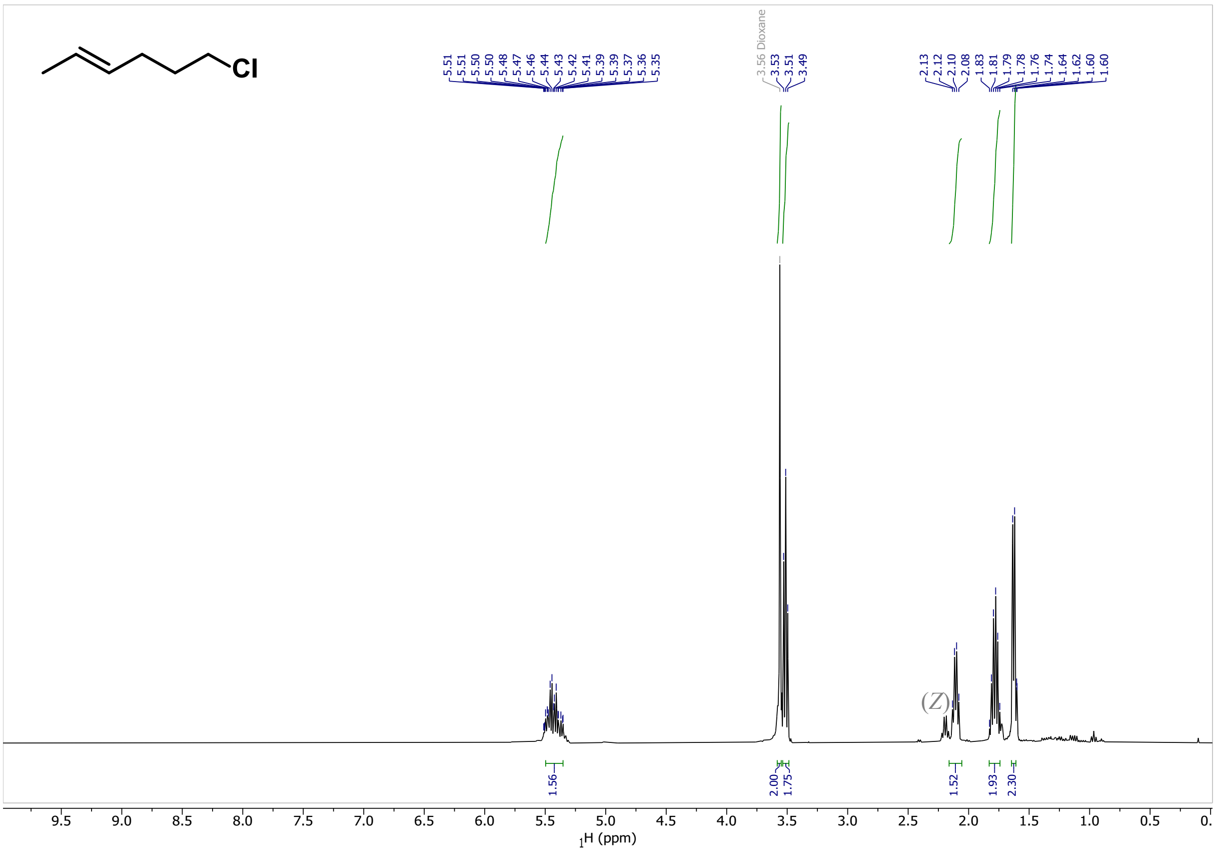

Figure S 65. ^{1}H NMR spectrum of **17a** (Z-isomer annotated).

Ethylidenecyclohexane (18a)

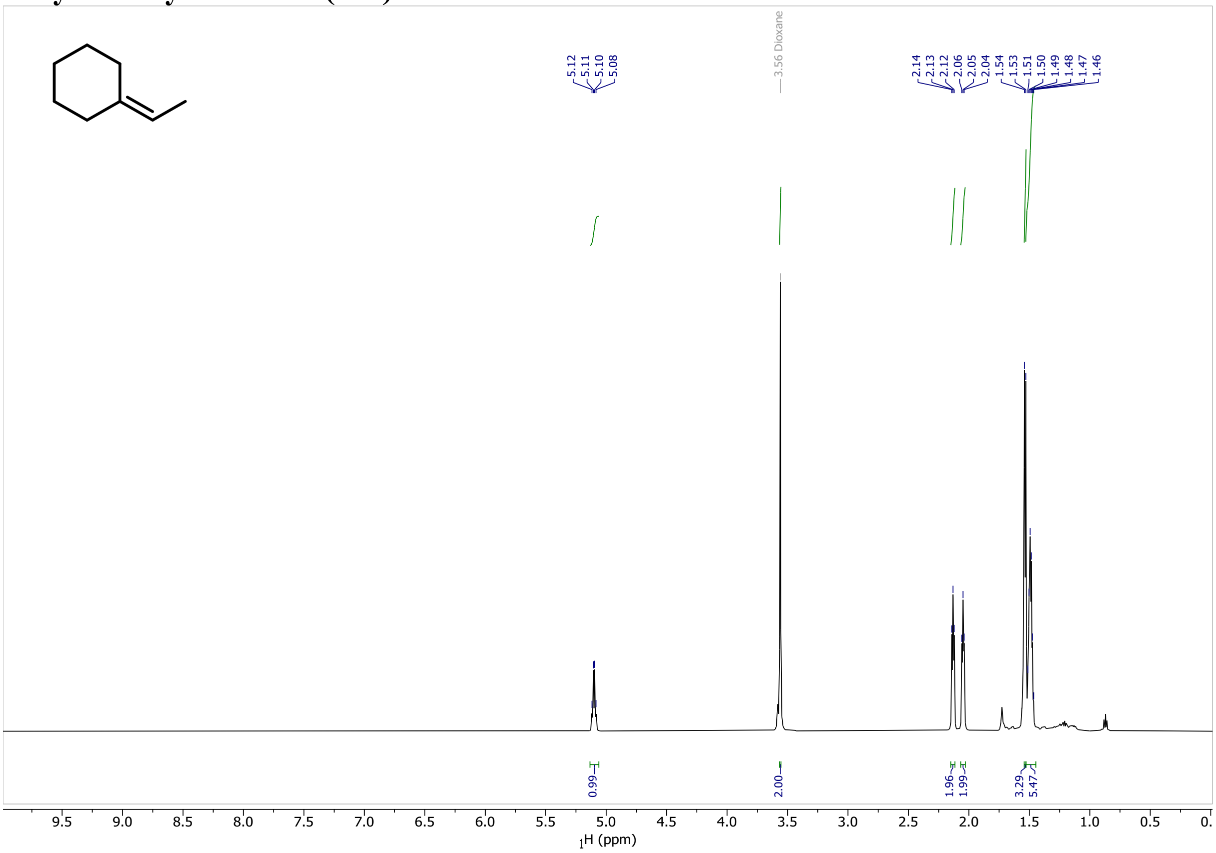

Figure S 66. ^{1}H NMR spectrum of **18a**.

(*Z*)-Trimethyl(pent-2-en-3-yloxy)silane (19a)

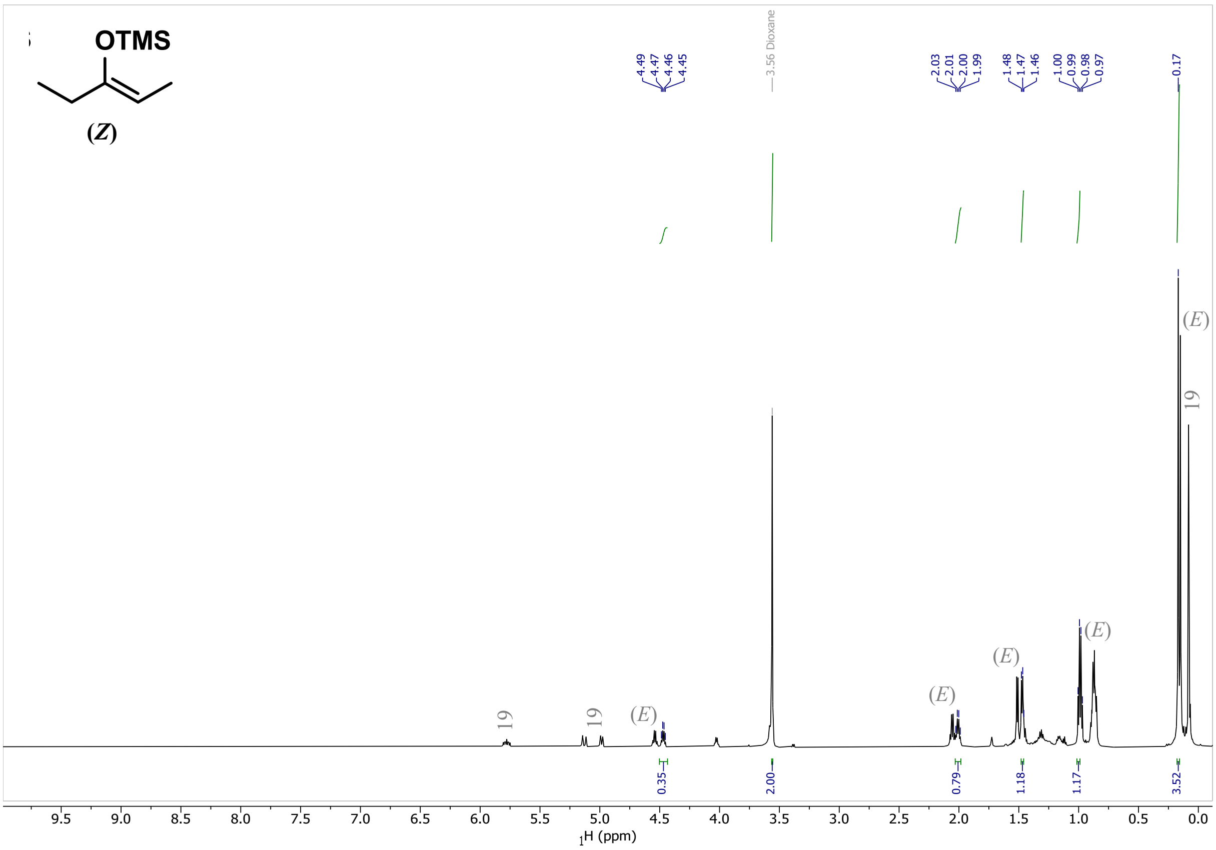

Figure S 67. ^{1}H NMR spectrum of **19a**.

(*E*)-1-(Prop-1-en-1-yl)adamantane (20a)

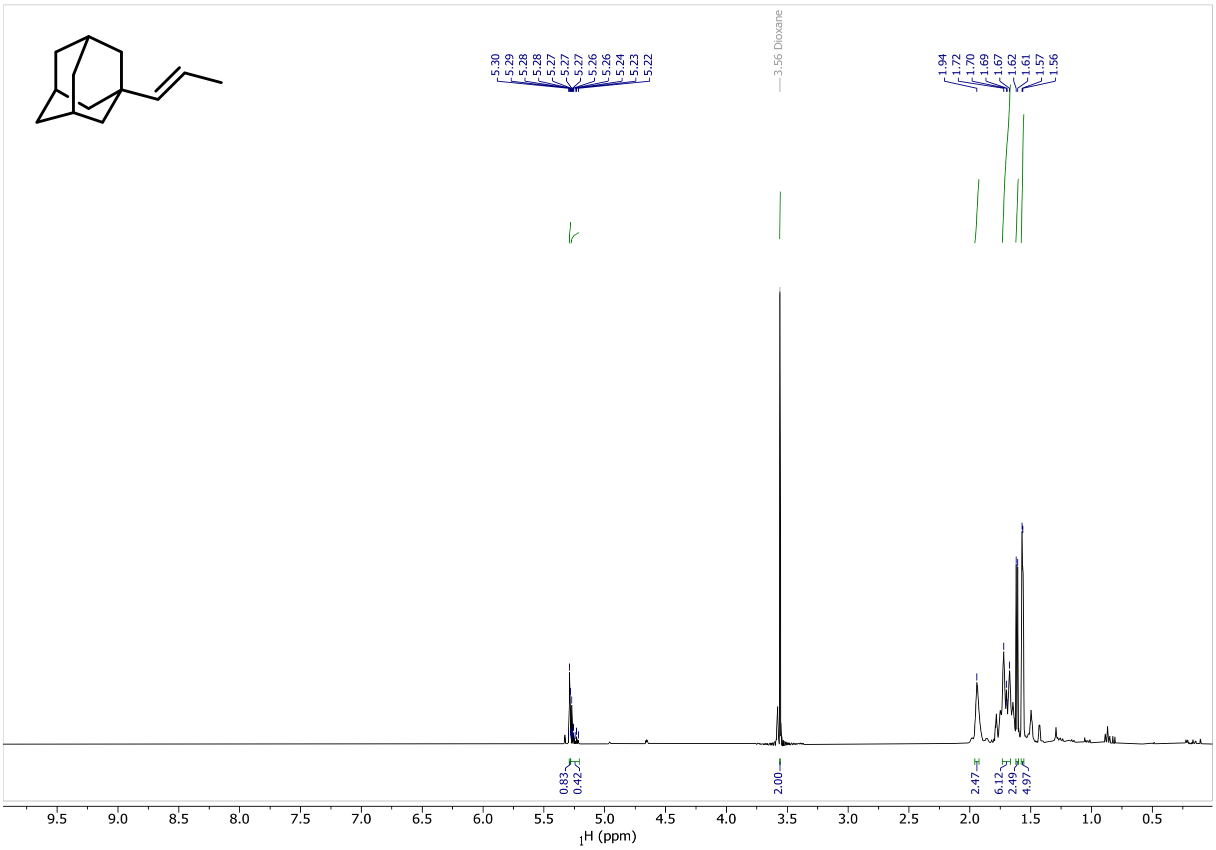

Figure S 68. ^{1}H NMR spectrum of **20a**.

(*E*)-Trimethyl(prop-1-en-1-yl)silane (21a)

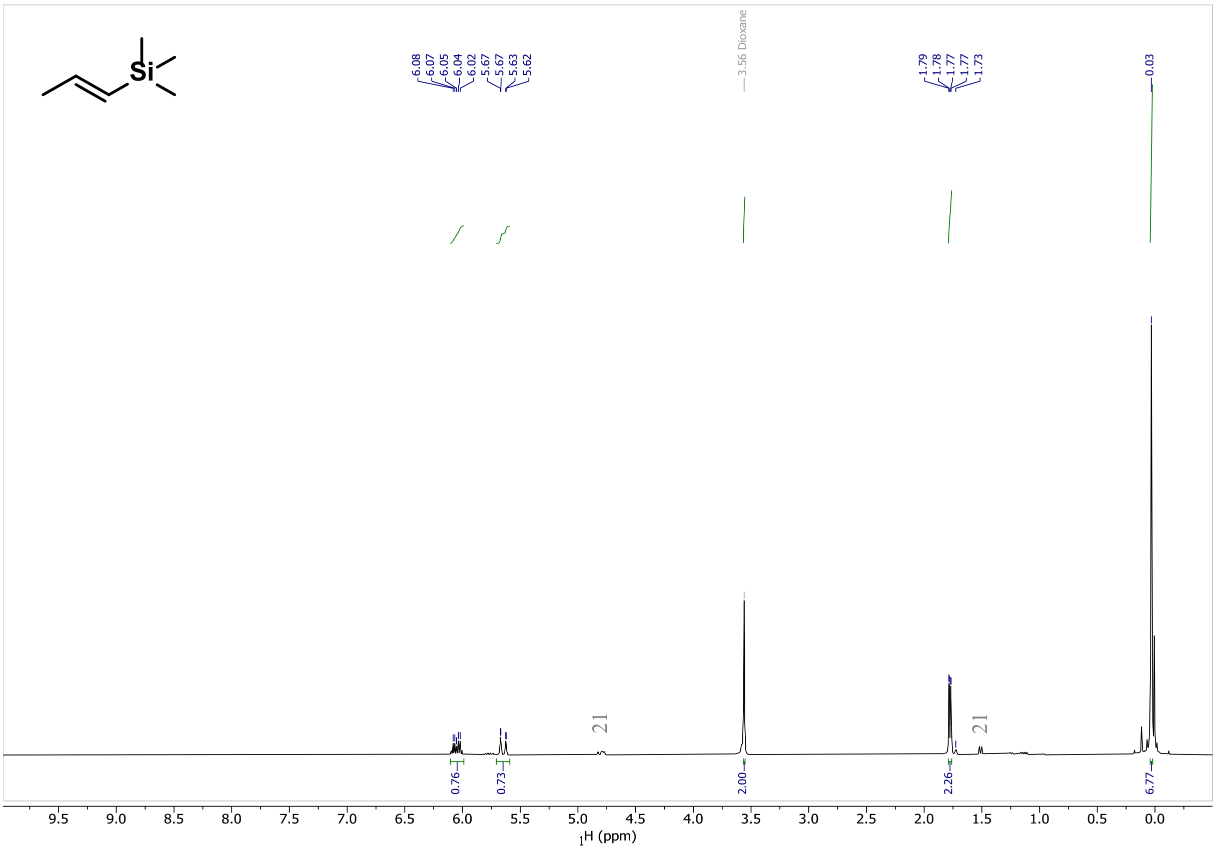

Figure S 69. ^{1}H NMR spectrum of **21a** (residual starting material **21** annotated).

5.4 Spectra of Chain-Walking Isomerization Products

(*E*)-But-1-en-1-yl-benzene (12b)

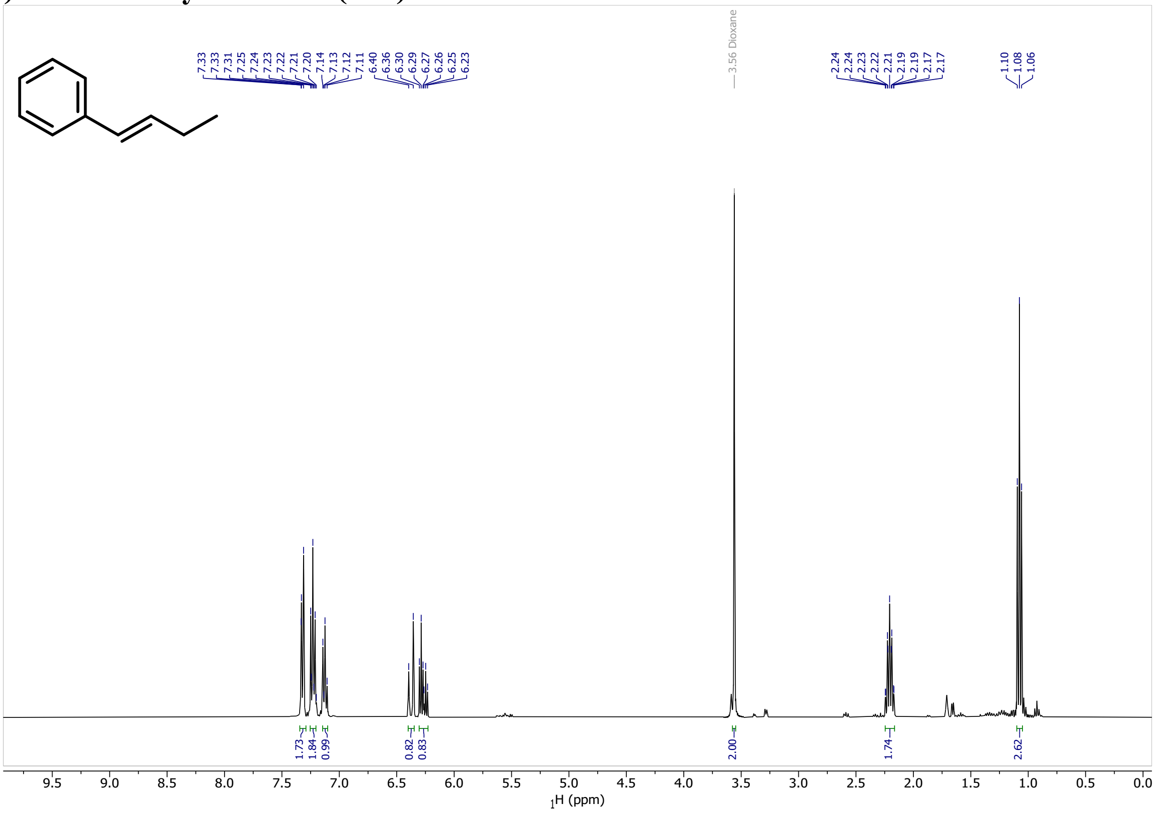

Figure S 70. ^{1}H NMR spectrum of **12b**.

(*E*)-Pent-2-en-2-ylbenzene (13b)

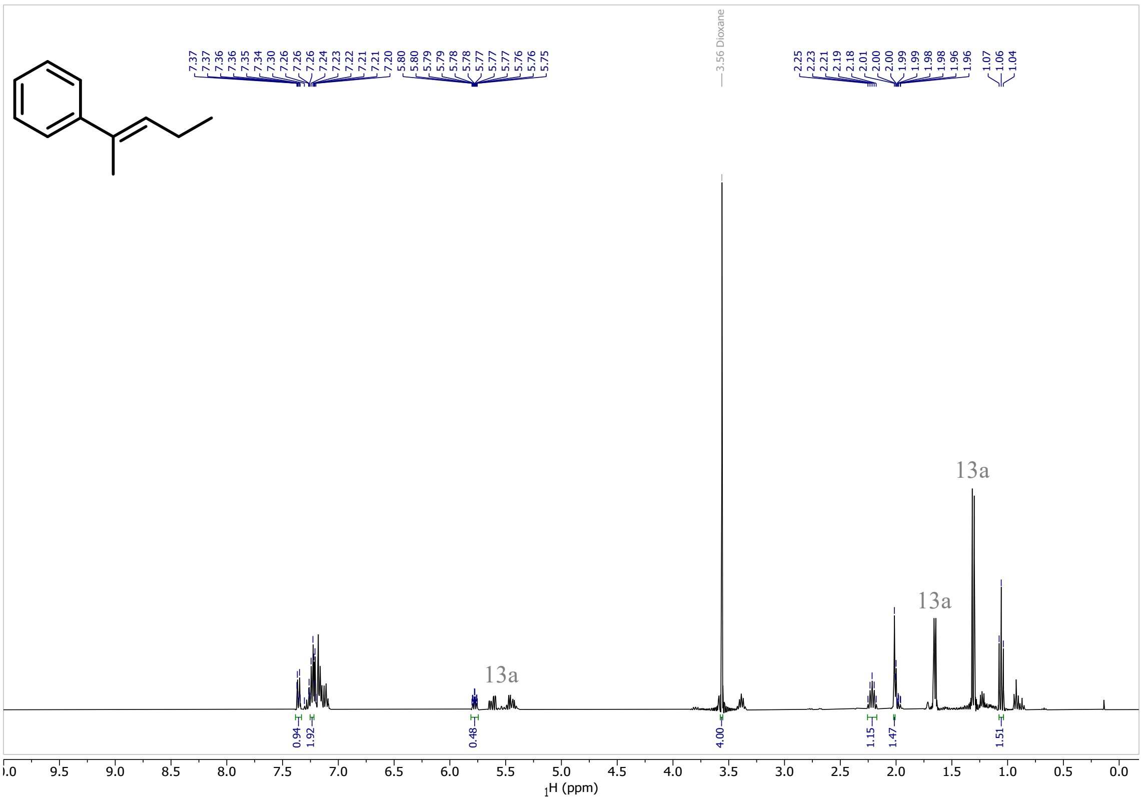

Figure S 71. ^{1}H NMR spectrum of **13b**. NMR spectrum contains 0.026 mmol of 1,4-dioxane (0.5 equiv. in respect to the substrate) as internal standard.

6 References

(1) Weber, S.; Stöger, B.; Veiros, L. F.; Kirchner, K. Rethinking Basic Concepts—Hydrogenation of Alkenes Catalyzed by Bench-Stable Alkyl Mn(I) Complexes. *ACS Catal.* **2019**, *9*, 9715–9720.

(2) Garduño, J. A.; García, J. J. Non-Pincer Mn(I) Organometallics for the Selective Catalytic Hydrogenation of Nitriles to Primary Amines. *ACS Catal.* **2019**, *9*, 392–401.

(3) Weber, S.; Zobernig, D.; Stöger, B.; Veiros, L. F.; Kirchner, K. Hydroboration of Terminal Alkenes and Trans-1,2-Diboration of Terminal Alkynes Catalyzed by a Manganese(I) Alkyl Complex. *Angew. Chem. Int. Ed.* **2021**, *60*, 24488–24492.

(4) Lin, S.; Song, C.-X.; Cai, G.-X.; Wang, W.-H.; Shi, Z.-J. Intra/Intermolecular Direct Allylic Alkylation via Pd(II)-Catalyzed Allylic C−H Activation. *J. Am. Chem. Soc.* **2008**, *130*, 12901–12903.

(5) Sai, M.; Yorimitsu, H.; Oshima, K. Copper-Catalyzed Allylation of Alkyl Halides with Allylic Grignard Reagents. *Bull. Chem. Soc. Jpn.* **2009**, *82*, 1194–1196.

(6) Xiong, T.; Li, Y.; Mao, L.; Zhang, Q. Palladium-Catalyzed Allylic C–H Amination of Alkenes with N-Fluorodibenzenesulfonimide: Water Plays an Important Role. *Chem. Commun.* **2012**, *48*, 2246–2248.

(7) Kawamura, K. E.; Chang, A. S.; Martin, D. J.; Smith, H. M.; Morris, P. T.; Cook, A. K. Modular Ni(0)/Silane Catalytic System for the Isomerization of Alkenes. *Organometallics* **2022**, *41*, 486–496.

(8) Breunig, M.; Yuan, P.; Gaich, T. An Unexpected Transannular [4+2] Cycloaddition during the Total Synthesis of (+)-Norcembrene 5. *Angew. Chem. Int. Ed.* **2020**, *59*, 5521–5525.

(9) Qin, L.; Sharique, M.; Tambar, U. K. Controllable, Sequential, and Stereoselective C–H Allylic Alkylation of Alkenes. *J. Am. Chem. Soc.* **2019**, *141*, 17305–17313.

(10) Kustiana, B. A.; Elsherbeni, S. A.; Linford-Wood, T. G.; Melen, R. L.; Grayson, M. N.; Morrill, L. C. B(C6F5)3-Catalyzed E-Selective Isomerization of Alkenes. *Chem. – Eur. J.* **2022**, *28*, e202202454.

(11) Chitnis, S. S.; LaFortune, J. H. W.; Cummings, H.; Liu, L. L.; Andrews, R.; Stephan, D. W. Phosphorus Coordination Chemistry in Catalysis: Air Stable P(III)-Dications as Lewis Acid Catalysts for the Allylation of C–F Bonds. *Organometallics* **2018**, *37*, 4540–4544.

(12) Jereb, M. Highly Atom Economical Uncatalysed and I2-Catalysed Silylation of Phenols, Alcohols and Carbohydrates, Using HMDS under Solvent-Free Reaction Conditions (SFRC). *Tetrahedron* **2012**, *68*, 3861–3867.

(13) Kuciński, K.; Stachowiak, H.; Hreczycho, G. Silylation of Alcohols, Phenols, and Silanols with Alkynylsilanes – an Efficient Route to Silyl Ethers and Unsymmetrical Siloxanes. *Eur. J. Org. Chem.* **2020**, *2020*, 4042–4049.

(14) Ohmura, T.; Yamamoto, Y.; Miyaura, N. Stereoselective Synthesis of Silyl Enol Ethers via the Iridium-Catalyzed Isomerization of Allyl Silyl Ethers. *Organometallics* **1999**, *18*, 413–416.

(15) Bergamaschi, E.; Beltran, F.; Teskey, C. J. Visible-Light Controlled Divergent Catalysis Using a Bench-Stable Cobalt(I) Hydride Complex. *Chem. – Eur. J.* **2020**, *26*, 5180–5184.

(16) Wu, Q.; Wang, L.; Jin, R.; Kang, C.; Bian, Z.; Du, Z.; Ma, X.; Guo, H.; Gao, L. Nickel-Catalyzed Allylic C(Sp2)–H Activation: Stereoselective Allyl Isomerization and Regiospecific Allyl Arylation of Allylarenes. *Eur. J. Org. Chem.* **2016**, *2016*, 5415–5422.

(17) Su, M.-D.; Liu, Y.-F.; Nie, Z.-W.; Yang, T.-L.; Cao, Z.-Z.; Li, H.; Luo, W.-P.; Liu, Q.; Guo, C.-C. Regioselective Synthetic Approach to Higher Alkenes from Lower Alkenes with Sulfoxides in the Fe3+/H2O2 System via Direct Alkylation or Arylation of the Csp2–H Bond on the C═C Bond of Alkenes. *J. Org. Chem.* **2022**, *87*, 7022–7032.

(18) Fu, S.; Chen, N.-Y.; Liu, X.; Shao, Z.; Luo, S.-P.; Liu, Q. Ligand-Controlled Cobalt-Catalyzed Transfer Hydrogenation of Alkynes: Stereodivergent Synthesis of Z- and E-Alkenes. *J. Am. Chem. Soc.* **2016**, *138*, 8588–8594.

(19) Tian, X.; Karl, T. A.; Reiter, S.; Yakubov, S.; de Vivie-Riedle, R.; König, B.; Barham, J. P. Electro-Mediated PhotoRedox Catalysis for Selective C(Sp3)–O Cleavages of Phosphinated Alcohols to Carbanions. *Angew. Chem. Int. Ed.* **2021**, *60*, 20817–20825.

(20) Lamb, J. R.; Hubbell, A. K.; MacMillan, S. N.; Coates, G. W. Carbonylative, Catalytic Deoxygenation of 2,3-Disubstituted Epoxides with Inversion of Stereochemistry: An Alternative Alkene Isomerization Method. *J. Am. Chem. Soc.* **2020**, *142*, 8029–8035.

(21) Liu, H.; Xu, M.; Cai, C.; Chen, J.; Gu, Y.; Xia, Y. Cobalt-Catalyzed Z to E Isomerization of Alkenes: An Approach to (E)-β-Substituted Styrenes. *Org. Lett.* **2020**, *22*, 1193–1198.

(22) Barbaro, G.; Battaglia, A.; Giorgianni, P. Lanthanide- and DMSO-Induced Ring Opening of 2-Iminooxetanes: Synthesis of .Beta.-Lactams and .Beta.-Keto Amides. *J. Org. Chem.* **1995**, *60*, 1020–1025.

(23) Shaffer, S. A.; Sadílek, M.; Tureček, F.; Hop, C. E. C. A. Protonation of Isomeric Methoxyhexenes. Effects of Double bond···H+···OCH3 Interaction. *Int. J. Mass Spectrom. Ion Process.* **1997**, *160*, 137–155.

(24) Tricoire, M.; Wang, D.; Rajeshkumar, T.; Maron, L.; Danoun, G.; Nocton, G. Electron Shuttle in N-Heteroaromatic Ni Catalysts for Alkene Isomerization. *JACS Au* **2022**, *2*, 1881–1888.

(25) Cambie, R. C.; Coulson, S. A.; Mackay, L. G.; Janssen, S. J.; Rutledge, P. S.; Woodgate, P. D. Recations of Cationic (H6-Chloroarene) Complexes of Iron and Ruthenium with Some O-Silyl and C-Silyl Compounds. *J. Organomet. Chem.* **1991**, *409*, 385–409.

(26) Thoma, G.; Curran, D. P.; Geib, S. V.; Giese, B.; Damm, W.; Wetterich, F. 1,2-Asymmetric Induction in Reactions of Nonconjugated Acyclic Radicals: A New Model for Highly Selective Atom-Transfer Reactions of Alkyl-Substituted Radicals. *J. Am. Chem. Soc.* **1993**, *115*, 8585–8591.

(27) Chen, C.; Dugan, T. R.; Brennessel, W. W.; Weix, D. J.; Holland, P. L. Z-Selective Alkene Isomerization by High-Spin Cobalt(II) Complexes. *J. Am. Chem. Soc.* **2014**, *136*, 945–955.

(28) Lu, Y.-C.; West, J. G. Chemoselective Decarboxylative Protonation Enabled by Cooperative Earth-Abundant Element Catalysis. *Angew. Chem. Int. Ed.* **2023**, *62*, e202213055.

(29) Gaussian 09, Revision A.02, Frisch, M. J.; Trucks, G. W.; Schlegel, H. B.; Scuseria, G. E.; Robb, M. A.; Cheeseman, J. R.; Scalmani, G.; Barone, V.; Mennucci, B.; Petersson, G. A.; Nakatsuji, H.; Caricato, M.; Li, X.; Hratchian, H. P.; Izmaylov, A. F.; Bloino, J.; Zheng, G.; Sonnenberg, J. L.; Hada, M.; Ehara, M.; Toyota, K.; Fukuda, R.; Hasegawa, J.; Ishida, M.; Nakajima, T.; Honda, Y.; Kitao, O.; Nakai, H.; Vreven, T.; Montgomery, Jr., J. A.; Peralta, J. E.; Ogliaro, F.; Bearpark, M.; Heyd, J. J.; Brothers, E.; Kudin, K. N.; Staroverov, V. N.; Kobayashi, R.; Normand, J.; Raghavachari, K.; Rendell, A.; Burant, J. C.; Iyengar, S. S.; Tomasi, J.; Cossi, M.; Rega, N.; Millam, J. M.; Klene, M.; Knox, J. E.; Cross, J. B.; Bakken, V.; Adamo, C.; Jaramillo, J.; Gomperts, R.; Stratmann, R. E.; Yazyev, O.; Austin,

A. J.; Cammi, R.; Pomelli, C.; Ochterski, J. W.; Martin, R. L.; Morokuma, K.; Zakrzewski, V. G.; Voth, G. A.; Salvador, P.; Dannenberg, J. J.; Dapprich, S.; Daniels, A. D.; Farkas, Ö.; Foresman, J. B.; Ortiz, J. V.; Cioslowski, J.; Fox, D. J. Gaussian, Inc., Wallingford CT, **2009**.

(30) Hehre, W. J.; Radom, L.; Schleyer, P. v.R. & Pople, J. A. *Ab Initio Molecular Orbital Theory*, John Wiley & Sons, NY, (1986).

(31) Parr, R. G. & Yang, W. in *Density Functional Theory of Atoms and Molecules*; Oxford University Press: New York, (1989).

(32) (a) Perdew, J. P.; Burke, K.; Ernzerhof, M. Generalized Gradient Approximation Made Simple *Phys. Rev. Lett.* **1996**, *77*, 3865-3868; (b) Perdew, J. P.; Burke, K.; Ernzerhof, M. Generalized Gradient Approximation Made Simple *Phys. Rev. Lett.* **1997**, *78*, 1396-1396. (c) Perdew, J. P. Density-functional approximation for the correlation energy of the inhomogeneous electron gas. *Phys. Rev. B* **1986**, *33*, 8822-8824.

(33) (a) Haeusermann, U.; Dolg, M.; Stoll, H.; Preuss, H.; Schwerdtfeger, P.; Pitzer, R. M. Accuracy of energy-adjusted quasirelativistic ab initio pseudopotentials *Mol. Phys.* **1993**, *78*, 1211-1224. (b) Kuechle, W.; Dolg, M.; Stoll, H.; Preuss, H. Energy-adjusted pseudopotentials for the actinides. Parameter sets and test calculations for thorium and thorium monoxide *J. Chem. Phys.* **1994**, *100*, 7535-7542. (c) Leininger, T.; Nicklass, A.; Stoll, H.; Dolg, M.; Schwerdtfeger, P. The accuracy of the pseudopotential approximation. II. A comparison of various core sizes for indium pseudopotentials in calculations for spectroscopic constants of InH, InF, and InCl *J. Chem. Phys.* **1996**, *105*, 1052-1059.

(34) (a) Ditchfield, R.; Hehre, W. J.; Pople, J. A. Self-Consistent Molecular-Orbital Methods. IX. An Extended Gaussian-Type Basis for Molecular-Orbital Studies of Organic Molecules *J. Chem. Phys.* **1971**, *54*, 724-728. (b) Hehre, W. J.; Ditchfield, R.; Pople, J. A. Self-Consistent Molecular Orbital Methods. 12. Further extensions of Gaussian-type basis sets for use in molecular-orbital studies of organic-molecules *J. Chem. Phys.* **1972**, *56*, 2257-2261. (c) Hariharan, P. C.; Pople, J. A. Accuracy of AH equilibrium geometries by single determinant molecular-orbital theory *Mol. Phys.* **1974**, *27*, 209-214. (d) Gordon, M. S. The isomers of silacyclopropane *Chem. Phys. Lett.* **1980**, *76*, 163-168. (e) Hariharan, P. C.; Pople, J. A. Influence of polarization functions on molecular-orbital hydrogenation energies *Theor. Chim. Acta* **1973**, *28*, 213-222.

(35) (a) Peng, C.; Ayala, P. Y.; Schlegel, H. B.; Frisch, M. J. Using redundant internal coordinates to optimize equilibrium geometries and transition states *J. Comp. Chem.* **1996**, *17*, 49-56. (b) Peng, C.; Schlegel, H. B. Combining Synchronous Transit and Quasi-Newton Methods for Finding Transition States *Israel J. Chem.* **1993**, *33*, 449-454.

(36) (a) McClean, A. D.; Chandler, G. S. Contracted Gaussian basis sets for molecular calculations. I. Second row atoms, Z=11-18 *J. Chem. Phys.* **1980**, *72*, 5639-5648. (b) Krishnan, R.; Binkley, J. S.; Seeger, R.; Pople, J. A. Self-consistent molecular orbital methods. XX. A basis set for correlated wave functions *J. Chem. Phys.* **1980**, *72*, 650-654. (c) Wachters, A. J. H. Gaussian Basis Set for Molecular Wavefunctions Containing Third-Row Atoms *J. Chem. Phys.* **1970**, *52*, 1033-1036. (d) Hay, P. J. Gaussian basis sets for molecular calculations - representation of 3D orbitals in transition-metal atoms *J. Chem. Phys.* **1977**, *66*, 4377-4384. (e) Raghavachari, K.; Trucks, G. W. Highly correlated systems: Excitation energies of first row transition metals Sc-Cu *J. Chem. Phys.* **1989**, *91*, 1062-1065. (f) Binning Jr., R. C.; Curtiss, L. A. Compact contracted basis-sets for 3rd-row atoms

- Ga-Kr *J. Comp. Chem.* **1990**, *11*, 1206-1216. (g) McGrath, M. P.; Radom, L. Extension of Gaussian-1 (G1) theory to bromine-containing molecules *J. Chem. Phys.* **1991**, *94*, 511-516. (h) Curtiss, L. A.; McGrath, M. P.; Blaudeau, J.-P.; Davis, N. E.; Binning Jr., R. C.; Radom, L. Extension of Gaussian-2 theory to molecules containing third-row atoms Ga-Kr *J. Chem. Phys.*, **1995**, *103*, 6104-6113. (i) Clark, T.; Chandrasekhar, J.; Spitznagel, G. W.; Schleyer, P. v. R. Efficient diffuse function-augmented basis-sets for anion calculations. 3. The 3-21+G basis set for 1st-row elements, Li-F *J. Comp. Chem.* **1983**, *4*, 294-301. (j) Frisch, M. J.; Pople, J. A.; Binkley, J. S. Self-Consistent Molecular Orbital Methods. 25. Supplementary Functions for Gaussian Basis Sets *J. Chem. Phys.* **1984**, *80*, 3265-3269.

(37) Zhao, Y.; Truhlar, D. G. The M06 suite of density functionals for main group thermochemistry, thermochemical kinetics, noncovalent interactions, excited states, and transition elements: two new functionals and systematic testing of four M06-class functionals and 12 other functionals *Theor. Chem. Acc.*, **2008**, *120*, 215-241.

(38) (a) Zhao, Y.; Truhlar, D. G. Density Functionals with Broad Applicability in Chemistry *Acc. Chem. Res.* **2008**, *41*, 157-167. (b) Zhao, Y.; Truhlar, D. G. Applications and validations of the Minnesota density functionals *Chem. Phys. Lett.* **2011**, *502*, 1-13.

(39) (a) Cancès, M. T.; Mennucci, B.; Tomasi, J. A new integral equation formalism for the polarizable continuum model: Theoretical background and applications to isotropic and anistropic dielectrics *J. Chem. Phys.* **1997**, *107*, 3032-3041. (b) Cossi, M.; Barone, V.; Mennucci, B.; Tomasi, J. Ab initio study of ionic solutions by a polarizable continuum dielectric model *Chem. Phys. Lett.* **1998**, *286*, 253-260. (c) Mennucci, B.; Tomasi, J. Continuum solvation models: A new approach to the problem of solute's charge distribution and cavity boundaries *J. Chem. Phys.* **1997**, *106*, 5151-5158. (d) Tomasi, J.; Mennucci, B.; Cammi, R. Quantum mechanical continuum solvation models *Chem. Rev.* **2005**, *105*, 2999-3094.

(40) Marenich, A. V.; Cramer, C. J.; Truhlar, D. G. Universal solvation model based on solute electron density and a continuum model of the solvent defined by the bulk dielectric constant and atomic surface tensions *J. Phys. Chem. B*, **2009**, *113*, 6378-6396.
